# *ARID1A* deficiency reprograms the tumor secretome, enhancing microenvironmental remodeling and metastatic dissemination in endometrial carcinoma

**DOI:** 10.1038/s41419-026-08723-z

**Published:** 2026-04-10

**Authors:** Cristina Megino-Luque, Manel Albertí-Valls, Sara Olave, Pol Sisó, Núria Bonifaci, Anna Macià, Xavier Matias-Guiu, Sònia Gatius, David Llobet-Navas, Núria Eritja

**Affiliations:** 1 Oncologic Pathology Group, Department of Medicine and Surgery, Biomedical Research Institute of Lleida (IRBLleida), University of Lleida, Lleida, Spain; 2 Department of Medicine, Division of Hematology and Oncology, The Tisch Cancer Institute, Icahn School of Medicine at Mount Sinai, New York, NY USA; 3 Centro de Investigación Biomédica en Red de Cáncer (CIBERONC), Madrid, Spain; 4 Oncologic Pathology Group, Department of Experimental Medicine, Biomedical Research Institute of Lleida (IRBLleida), University of Lleida, Lleida, Spain; 5 Molecular Mechanisms and Experimental Therapy in Oncology-Oncobell Program, Bellvitge Biomedical Research Institute (IDIBELL), Gran via De l’Hospitalet 199, Barcelona, Spain; 6 Department of Pathology, Hospital Universitari Arnau de Vilanova, Lleida, Spain; 7 Oncologic Pathology Group, Department of Basic Medical Sciences, Biomedical Research Institute of Lleida (IRBLleida), University of Lleida, Lleida, Spain

**Keywords:** Endometrial cancer, Gynaecological cancer

## Abstract

Loss-of-function mutations in *AT-rich interactive domain-containing protein 1* *A* (ARID1A), accompanied by reduced protein expression, are common in endometrial carcinoma (EC) and correlate with shorter progression-free survival. Alterations in the EC cells secretome play a critical role in shaping the tumor microenvironment (TME), thereby promoting disease progression, metastasis, and therapeutic resistance. Here, we demonstrate that ARID1A-deficient EC cells display a reprogrammed soluble secretome that alters tumor-stromal communication. Among the secreted factors, CXCL16 emerges as the predominant chemokine, promoting epithelial-to-mesenchymal transition and enhancing tumor cell invasiveness. Mechanistically, CXCL16 activates MAPK and Paxillin/FAK pathways, driving YAP/TAZ signaling and reinforcing pro-tumorigenic features. Elevated CXCL16 levels within the tumor niche also promote the conversion of stromal cells into cancer-associated fibroblasts (CAFs) in both preclinical models and patient samples of ARID1A-deficient EC. Importantly, genetic or pharmacological inhibition of CXCL16 or its receptor CXCR6 disrupts these pathogenic interactions, impairing EC cell migration and reducing metastatic burden. These findings identify the secretome of ARID1A-deficient EC cells as a key driver of tumor progression and underscore the CXCL16-CXCR6 axis as a promising therapeutic target in EC.

## Introduction

Worldwide, EC is the most prevalent invasive gynecologic malignancy, and its incidence has increased over the past few decades [1]. Although early diagnosis, surgery, and chemotherapy have improved EC outcomes, many patients still ultimately succumb to the disease. The mechanisms underlying the acquisition of aggressive transformation in EC tumor cells remain poorly understood. Molecular cues derived from EC malignant cells can reshape the TME, playing a pivotal role in the transition from an indolent tumor to a malignant state [2]. Tumor cells actively remodel the TME by recruiting and activating non-transformed cells, including mesenchymal stromal cells, fibroblasts, endothelial, adipocytes, smooth muscle cells and immune cells [3]. Besides non-malignant cells, the TME also includes the extracellular matrix (ECM), which is rich in collagens and other components that facilitate tumor progression [4]. Collectively, these changes favor disease dissemination and modulate biological processes that contribute to altered therapeutic responses and the development of chemoresistance [3].

Among all the cellular components, fibroblasts represent the predominant cell type within the uterine stromal compartment. During carcinogenesis, fibroblasts migrate to the neoplastic lesion, where they proliferate, increase collagen production and express alpha-smooth muscle actin (α-SMA) [5]. These changes constitute the desmoplastic response, a hallmark of CAFs. CAFs are a heterogeneous population characterized by various markers, whose activation (typically driven by neoplastic or immune cell-secreted factors), alters their morphology and function [6].

The term “secretome” refers to the entire set of proteins secreted or released into the extracellular space by a cell, tissue, or organism at any specific time [7]. It includes diverse bioactive molecules, mainly soluble factors (cytokines and growth factors), as well as extracellular vesicles, such as exosomes, which modulate interactions between cells and the ECM [8]. These factors can act in an autocrine or paracrine manner to influence cell survival, proliferation, and differentiation, playing essential roles in both normal physiology and disease pathogenesis, including cancer [9]. During tumorigenesis, the secretome composition changes due to both genetic mutations and non-mutational alterations that influence gene expression. For instance, a recent study revealed that undifferentiated uterine sarcomas harboring SWI/SNF complex mutations, particularly SMARCA4-deficient tumors, show increased levels of CXCL19 and CXCL12 in their secretome, which play a crucial role in modulating the TME [10].

ARID1A (AT-rich interactive domain) encodes a DNA-binding domain of the SWI/SNF chromatin remodeling complex [11]. SWI/SNF complex mutations are found in over 20% of human cancers, with ARID1A being the most frequently mutated, typically leading to loss of function. Notably, ARID1A loss-of-function mutations are highly prevalent in gynecological cancers, with a mutational frequency of 46,7% in low-grade EC and up to 60% in high-grade EC [12]. Although the precise role of ARID1A in EC remains unclear, emerging evidence suggests it is more closely associated with tumor progression rather than initiation, as observed in other tumor types [13, 14]. Additionally, some studies propose that ARID1A loss expression in EC promotes metastasis via activation of the epithelial-mesenchymal transition (EMT) pathway, which is associated with myometrial invasion and correlates with disease recurrence and distant metastasis [13, 15, 16]. These observations support a broader concept in which EMT, the tumor secretome, and TME remodeling converge to establish pre-metastatic niches even before metastatic seeding occurs [17].

Here, we show that the loss of ARID1A expression in endometrial carcinogenesis modifies the tumor cell secretome, enhancing cellular plasticity not only in adjacent ARID1A-wildtype tumor cells but also promoting CAF activation, thereby remodeling the TME and facilitating EC progression and dissemination. This study integrates TCGA datasets and human EC biopsies with functional assays in cell lines, patient-derived samples, and genetically engineered mouse models to elucidate the role of the ARID1A-deficient secretome in driving TME remodeling and metastatic progression.

## Material And Methods

Detailed materials and methods are provided in the Supplementary Information.

## Results

### ARID1A-deficient EC cells promote more aggressive phenotypes in surrounding ARID1A-wild-type EC cells

Given the key role of secreted non-vesicular soluble proteins in promoting tumor aggressiveness, migration and invasion [18], we hypothesized that the soluble-factor secreted from ARID1A-deficient EC cells may modulate the behavior of adjacent ARID1A-wild-type EC cells.

**Fig. 1:**
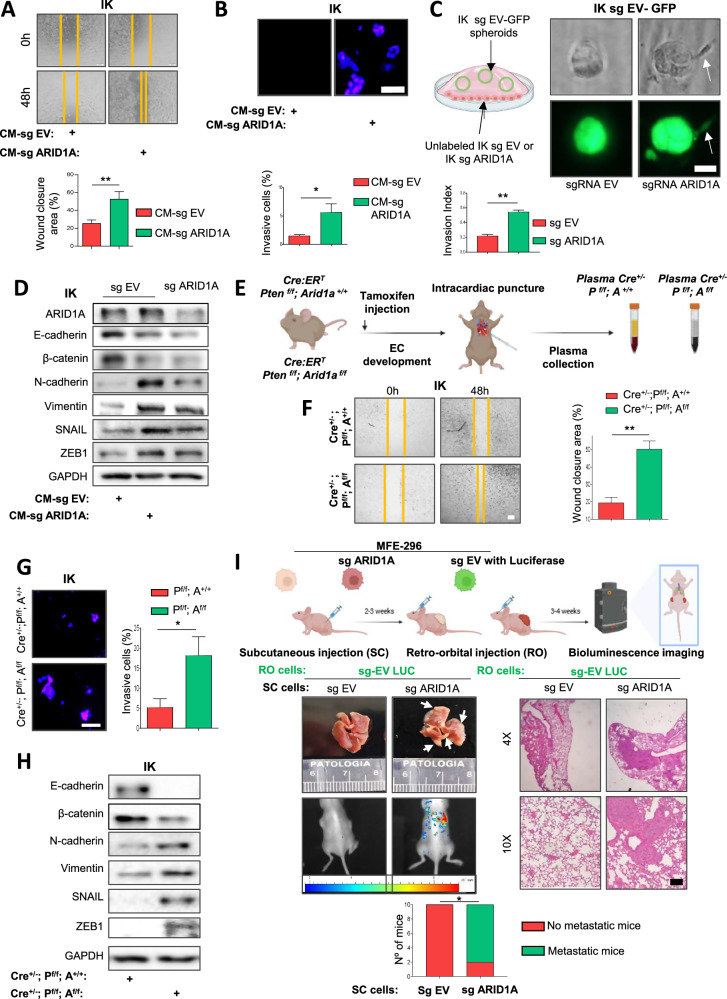
ARID1A-deficient EC cells promote more aggressive phenotypes in surrounding ARID1A-wild-type EC cells. **A** Representative images at time 0 and 48 h after scratch in wound-healing assay performed in IK cells treated with conditioned media collected from IK cells infected with sgARID1A (CM-sg ARID1A) or empty vector (CM-sg EV). Bottom plot shows quantification of wound closure. Scale bars: 200 μm. **B** Hoechst-stained transwell invasion assay of IK control cells chemoattracted by CM-sg EV or CM-sg ARID1A IK for 48 h (upper panel), and quantification of Matrigel®-invading cells (bottom plot). Scale bars: 50 μm. **C** Schematic (left) and phase-contrast/immunofluorescence images (GFP) of spheroids from GFP-labeled IK cells co-cultured with unlabeled IK cells infected with sgRNA EV or sgRNA ARID1A (right). The bottom plot shows quantification of the invasion index. Scale bars: 25 μm. **D** Western blot analysis of ARID1A, E-cadherin, β-catenin, N-cadherin, vimentin, SNAIL and ZEB n in IK cells treated with CM-sg EV or CM-sg ARID1A for 48 h. GAPDH was used as a loading control. **E** Schematic of plasma collection. *Cre:ER*^*T*^*; Pten*^*f/f*^*; Arid1a*^*+/+*^ or *Cre:ER*^*T*^*; Pten*^*f/f*^*; Arid1a*^*f/f*^ mice were weaned at 3 weeks and received tamoxifen at 8 weeks to induce epithelial-specific deletion. Plasma was collected 8 weeks later, when tumors had developed [13]. **F** Representative images at time 0 and 48 h after scratch in wound-healing assay of IK treated with *Cre*^*+/-*^*; Pten*^*f/f*^*; Arid1a*^*+/*^ or *Cre*^*+/-*^*; Pten*^*f/f*^*; Arid1a*^*f/f*^ plasmas. Scale bars: 200 μm. **G** Hoechst-stained transwell invasion assay of IK cells pretreated for 48 h with plasma from *Cre*^*+/-*^*;Pten*^*f/f*^*; Arid1a*^*+/*^ or *Cre*^*+/-*^*;Pten*^*f/f*^*; Arid1a*^*f/f*^ mice (left), and quantification of Matrigel®-invading cells (right). Scale bars: 50 μm. **H**) Western blot of E-cadherin, β-catenin, N-cadherin, vimentin, SNAIL and ZEB in IK cells treated for 48 h with plasma from the indicated mice. GAPDH was used as a loading control. **I** Schematic (top) of in vivo experimental design. 1.5 × 104 unlabeled MFE-296 sgRNA EV or sgARID1A (*n* = 10 each) were injected subcutaneously (SC) and allowed to grow for 3 weeks. Then, 5 × 10^5^ GFP-luciferase-labeled MFE-296 sgRNA EV cells were injected retro-orbitally (RO) into all mice. Tumor dissemination was assessed by bioluminescence, lung images, H&E sections, and quantification of lung metastases. For the *i*n vivo experiment (*n* = 10 per group), statistical significance was assessed using the Chi-square test All graphs represent mean ± S.E.M. Statistical significance in in vitro experiments involving endometrial cancer cell lines and treatment conditions was performed using unpaired two-tailed Student’s t-test. Statistical significance is indicated as *p < 0.05; **p < 0.01. Results are representative of three independent experiments with three technical replicates per experiment.

To test this, we first collected conditioned media (CM) from IK and MFE-296 EC cell lines infected with lentiviruses carrying either an sgRNA empty vector (CM-sg EV) or sgRNA targeting ARID1A (CM-sg ARID1A). As shown in Fig. 1A and Supplementary Fig. 1A, treatment with CM-sg ARID1A significantly enhanced the migratory capacity of ARID1A-wildtype cells compared with CM-sg EV-treated cells. Supporting these results, in transwell and three-dimensional (3D) co-culture assays, CM-sg ARID1A also promoted the invasive behavior of ARID1A-wild-type cells (Fig. 1B, C and Supplementary Fig. 1B). Next, we analyzed the EMT phenotype in these cells. We observed that CM-sg ARID1A, but not CM-sg EV, induced a downregulation of several epithelial markers (such as E-cadherin and β-catenin) and an upregulation of mesenchymal markers (such as N-cadherin and Vimentin), together with pro-EMT transcription factors (such as SNAIL1 and ZEB1) in ARID1A-wild-type EC cells (Fig. 1D and Supplementary Fig. 1C).

As soluble factors from primary tumor cells play a pivotal role in both early and advanced metastatic stages, promoting intravasation, circulation, extravasation, and premetastatic niche formation [19], we hypothesized that pro-tumorogenic signals from EC cells with altered ARID1A expression might reach the circulation and promote tumor aggressiveness. To test this, *Cre:ER*^*T+/-*^*; Pten*^*f/f*^*; Arid1a*^*+/+*^ (Cre^+/-^; P^f/f^; A^+/+^) or *Cre:ER*^*T*^*; Pten*^*f/f*^*; Arid1a*^*f/f*^ (Cre^+/-^; P^f/f^; A^f/f^) mice were administrated a single tamoxifen dose. After 8 weeks, when all mice developed endometrial tumors [13], plasmas were collected (Fig. 1E). ARID1A loss in the uterus was confirmed by immunohistochemistry (Supplementary Fig. 1D). IK and MFE 296 ARID1A wild-type EC cells were treated with the collected plasmas, and we observed that plasmas from Cre^+/-^; P^f/f^; A^f/f^ mice significantly increased the migratory and invasive capacities of these cells compared to plasmas from Cre^+/-^; P^f/f^; A^+/+^ mice (Fig. 1F, G and Supplementary Fig. 1E, F). Additionally, treatment with plasmas from Cre^+/-^; P^f/f^; A^f/f^ mice also promoted the acquisition of EMT markers in IK or MFE 296 ARID1A wild-type cells (Fig. 1H and Supplementary Fig. 1G).

Next, we tested whether these results were recapitulated in vivo. For this purpose, non-labeled MFE-296 sg-EV or sg-ARID1A cells were injected subcutaneously into SCID mice. Once the subcutaneous tumors were established, EGFP/luciferase-tagged MFE-296 sg-EV cells were injected retro-orbitally into all mice (Fig. 1I). After two to three weeks, lung metastatic foci derived from EGFP/luciferase-tagged MFE-296 sg-EV cells were detected exclusively in those mice bearing subcutaneous MFE-296 sg-ARID1A tumors (Fig. 1I). These findings indicate that the secretome of ARID1A-deficient EC cells releases pro-tumorigenic signals into the bloodstream, thereby promoting metastatic colonization.

### ARID1A loss alters the soluble secretome composition in EC cells

**Fig. 2:**
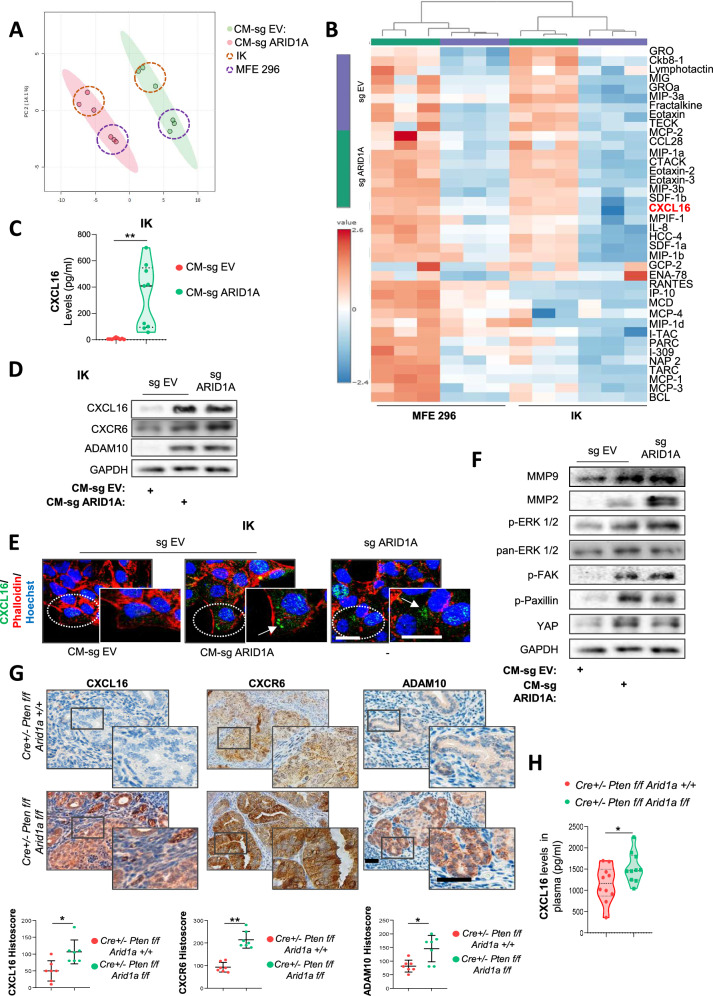
ARID1A loss modifies the soluble secretome composition in EC cells. **A** Principal component analysis (PCA) of chemokine array results showing change in the secreted profile of conditioned media (CM) collected after 48 h of IK and MFE-296 cells infected with lentivirus carrying sgRNA against ARID1A (lentiCRISPRv2-ARID1A; CM-sg ARID1A) or empty vector (lentiCRISPRv2; CM-sg EV). **B** Heatmap showing hierarchical clustering of normalized chemokine array signals from CM-sg EV and CM-sg ARID1A of IK and MFE-296 cells. **C** ELISA assay for CXCL16 in conditioned media collected from IK cells with either wild-type or altered ARID1A expression. Statistical significance was calculated using an unpaired two-tailed Student’s t-test. *N* = 9 per group. **D** Representative immunoblotting images of CXCL16, CXCR6 and ADAM10 in IK sgRNA EV or IK sgRNA ARID1A treated with CM-sg EV or CM-sg ARID1A for 48 h. GAPDH was used as a loading control. **E** Representative images of immunofluorescence against CXCL16 in IK sgRNA EV or IK sgRNA ARID1A cells treated with CM-sg EV or CM-sg ARID1A for 48 h. Scale bars: 25 μm. **F** Representative immunoblotting images of MMP9, MMP2, p-ERK 1/2, pan-ERK 1/2, p-FAK, p-Paxillin and YAP in IK sgRNA EV or IK sgRNA ARID1A treated with CM-sg EV or CM-sg ARID1A for 48 h. GAPDH was used as a loading control. **G** Representative images (top) and quantification (bottom) of CXCL16, CXCR6, and ADAM10 immunohistochemistry in consecutive sections of EC uteri from *Cre*^*+/-*^*; Pten*^*f/f*^*; Arid1a*^*+/*^ and *Cre*^*+/-*^*; Pten*^*f/f*^*; Arid1a*^*f/f*^ mice. Scale bars: 250μm. Statistical analysis was performed using an unpaired two-tailed Student’s t-test for each comparison (*n* = 7 per group). **H** ELISA assay for CXCL16 on *Cre*^*+/-*^*; Pten*^*f/f*^*; Arid1a*^*+/*^ and *Cre*^*+/-*^*; Pten*^*f/f*^*; Arid1a*^*f/f*^ plasma mice. *N* = 10 per group. Statistical significance was assessed by the Wilcoxon rank-sum test. All graphs represent mean ± S.E.M., and statistical significance is indicated as Student’s t-test *p < 0.05; **p < 0.01. Results are representative of three independent experiments with three technical replicates per experiment.

Given the impact of ARID1A-deficient CM on endometrial tumor cell behavior and aggressiveness, we analyzed the chemokine secretome prolife of ARID1A-deficient IK and MFE-296 cells. To this end, we conducted a chemokine array on CM collected from ARID1A-deficient and wild-type IK and MFE-296 cells. Analysis showed that ARID1A status modulates the overall chemokine secretion profile (Fig. 2A), with ARID1A-deficient cells displaying moderately increased levels of most chemokines compared with wild-type cells (Fig. 2B). Notably, CXC chemokine ligand 16 (CXCL16) levels were consistently elevated in ARID1A-altered EC cells compared with wild-type cells (Supplementary Fig. 2A). ELISA assay performed on CM from wild-type and ARID1A-downregulated cells further confirmed significantly increased CXCL16 secretion upon ARID1A loss (Fig. 2C and Supplementary Fig. 2B).

Overexpression of CXCL16 has been reported in various tumors, where it contributes to TME modulation and promotes angiogenesis, tumor progression, and metastasis [20, 21]. Its transmembrane form is cleaved by ADAM10, generating a soluble form that binds CXCR6 to mediate its biological functions [22]. To investigate the involvement of the CXCL16/CXCR6 axis in ARID1A-deficient and wild-type cells, we analyzed its expression and observed that both ARID1A-deficient IK and MFE-296 cells, as well as wild-type cells treated with CM-sgARID1A, exhibited increased expression of CXCL16, CXCR6, and ADAM10 at both protein and mRNA levels compared to their respective controls (Fig. 2D, E and Supplementary Fig. 2C).

Activation of the CXCL16/CXCR6 axis via MAPK pathway promotes stress fiber formation, actin-myosin assembly, and focal adhesion stabilization, thereby enhancing p-Paxillin/FAK signaling and inducing YAP/TAZ activation and nuclear translocation. This cascade drives the overexpression of MMP2, MMP9, ADAM10, CXCR6, and CXCL16, establishing a positive feedback loop [22–24]. To assess the involvement of this pathway, we examined p-Paxillin/FAK and YAP/TAZ expression in IK wild-type cells treated with CM-sg EV or CM-sgARID1A, and in ARID1A-deficient cells. Cells exposed to CM-sgARID1A or lacking ARID1A showed increased levels of pathway-related proteins (including MMP9, MMP2, p-ERK, p-FAK, YAP, and p-Paxillin) compared to wild-type cells treated with CM-sgEV (Fig. 2F). Similar results were observed in wild-type IK cells treated with plasmas from Cre^+/-^; P^f/f^; A^f/f^ mice (Supplementary Fig. 2D), supporting a potential role for CXCL16 secreted by ARID1A-deficient EC cells in mediating these phenotype changes.

Moreover, immunofluorescence analysis confirmed both the activation and nuclear translocation of YAP, accompanied by increased expression of p-Paxillin, which promote the formation of focal adhesions at the plasma membrane (Supplementary Fig. 2E). Additionally, we observed enhanced association of p-MLY2 with actin stress fibers in ARID1A wild-type cells treated with CM-sg ARID1A, as well as in ARID1A-deficient cells, compared to their respective controls (Supplementary Fig. 2E).

To validate the activation of the CXCL16/CXCR6 pathway in vivo, we assessed the expression levels of CXCL16, CXCR6, and ADAM10 in our *Cre:ER*^*T*^*; Pten*^*f/f*^*; Arid1a*^*f/f*^ mice model. As expected, *Cre:ER*^*T*^*; Pten*^*f/f*^*; Arid1a*^*f/f*^ mice exhibited increased expression of CXCL16, CXCR6, and ADAM10 (Fig. 2G). Furthermore, ELISA analysis confirmed significantly elevated CXCL16 levels in plasma samples collected from *Cre:ER*^*T*^*; Pten*^*f/f*^*; Arid1a*^*f/f*^ mice compared with those collected from *Cre:ER*^*T*^*; Pten*^*f/f*^*; Arid1a*^*+/+*^ mice (Fig. 2H).

### CXCL16 promotes a more aggressive phenotype in EC cells

**Fig. 3:**
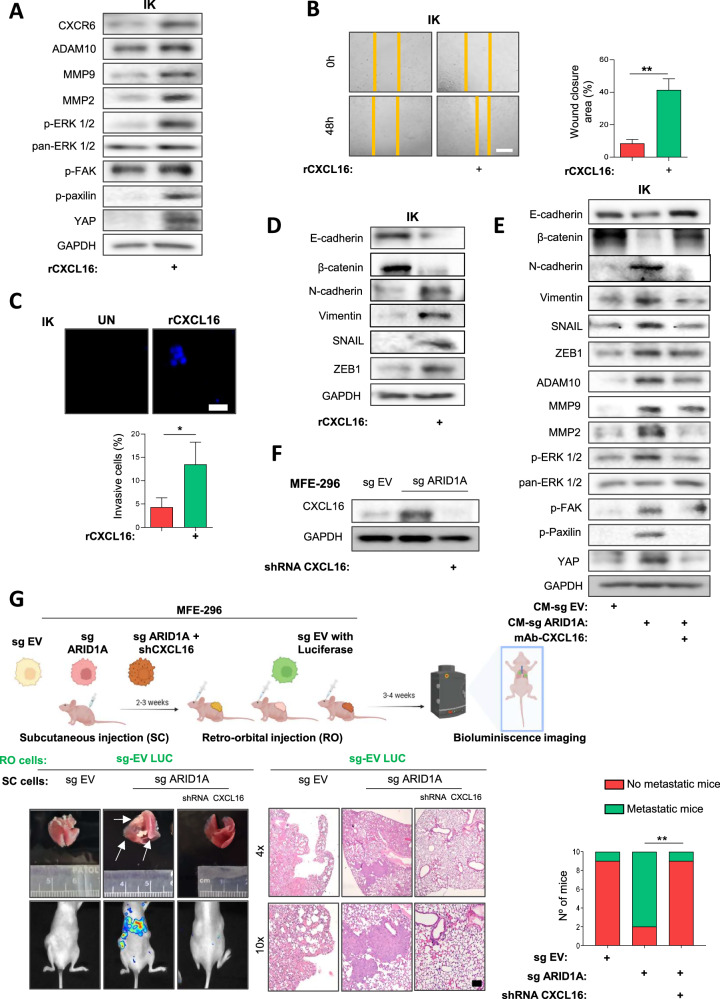
CXCL16 enhances aggressiveness in EC cells. **A** Representative immunoblotting image showing activation of the CXCL16/CXCR6 axis and downstream signaling pathways in IK cells treated for 48 h with recombinant CXCL16 (rCXCL16) (100 ng/ml). Immunoblots include CXCR6, ADAM10, MMP9, MMP2, p-ERK1/2, pan-ERK1/2, p-FAK, p-Paxillin, and YAP. GAPDH was used as a loading control. **B** Representative images at time 0 and 48 h after scratch of wound-healing assay performed in IK cells treated with rCXCL16 (100 ng/ml). The right panel shows quantification of wound closure. Scale bars: 200 μm. Statistical analysis was performed using an unpaired two-tailed Student’s t-test. **C** Representative images of Hoechst-stained transwell invasion assays of IK cells treated with rCXCL16 (100 ng/ml) for 48 h (top), and quantification of Matrigel®-invading cells (bottom). Scale bars: 50μm. Statistical analysis was performed using an unpaired two-tailed Student’s t-test. **D** Western blot analysis of epithelial (E-cadherin, β-catenin) and mesenchymal (N-cadherin, Vimentin) markers, EMT-associated transcription factors (SNAIL, ZEB1) in IK cells treated with rCXCL16 (100 ng/ml) for 48 h. GAPDH was used as a loading control. **E** Western blot analysis of epithelial (E-cadherin, β-catenin) and mesenchymal (N-cadherin, Vimentin) markers, EMT-associated transcription factors (SNAIL, ZEB1) and key effectors of the CXCL16/CXCR6 axis and downstream signaling pathways (ADAM10, MMP9, MMP2, p-ERK1/2, p-FAK, p-Paxillin, YAP) in IK cells treated with CM-sg ARID1A in the presence or absence of a neutralizing antibody against CXCL16 (mAb-CXCL16). Treatment with mAb-CXCL16 (8 μg/ml) reverted EMT and reduced pathway activation induced by rCXCL16 (100 ng/ml). GAPDH was used as loading control. **F** Western blot analysis confirming CXCL16 silencing in ARID1A-deficient MFE-296 cells infected with lentiviral shRNA vectors targeting CXCL16 (shCXCL16). GAPDH was used as loading control. **G** Schematic representation of the in vivo experimental design (top). Subcutaneous tumors (SC) were generated using ARID1A-deficient MFE-296 cells with or without stable CXCL16 silencing (shCXCL16) co-infection. After 3-4 weeks, luciferase-labeled wild-type MFE-296 cells were injected retro-orbital (RO) into mice. Tumor dissemination was monitored 3 weeks later. Representative bioluminescence imaging, macroscopic lungs, and H&E-stained lung sections showing metastatic foci. Bottom right panel shows quantification of the number of mice presenting lung metastases in each group. Scale bars: 250 μm. Statistical analysis was performed using the Chi-square test for overall group comparison, followed by pairwise comparisons with Bonferroni correction, *n* = 10 per group. All graphs represent mean ± S.E.M., and statistical significance is indicated as Student’s t-test *p < 0.05; **p < 0.01. Results are representative of three independent experiments with three technical replicates per experiment.

To confirm that CXCL16 was responsible for the increased migratory and invasive capacities, as well as the EMT transformation in ARID1A-deficient EC cells, we treated wild-type ARID1A EC cells with recombinant CXCL16 protein (rCXCL16). First, we confirmed that the addition of the rCXCL16 activated the CXCL16/CXCR6 axis, leading to enhanced activation of the MAPK and p-Paxillin/FAK signaling pathways (Fig. 3A). Next, we assessed the invasive and migratory capabilities of wild-type ARID1A EC cells treated with rCXCL16. As shown in Fig. 3B, C, rCXCL16 treatment significantly increased the migration and invasion abilities of the EC cell lines. Finally, we corroborated that treatment with rCXCL16 resulted in reduced expression of epithelial markers and increased expression of mesenchymal markers, and upregulation of several pro-EMT transcription factors (Fig. 3D).

To further investigate the involvement of CXCL16 in activating the CXCL16/CXCR6 axis, its downstream signaling pathways, and EMT-related phenotypes, we treated ARID1A-wildtype EC cells with CM-sg ARID1A, either alone or in combination with CXCL16 neutralizing antibody (mAb-CXCL16) or the selective CXCR6 antagonist ML-339. Migration and invasion assays showed that both mAb-CXCL16 (Supplementary Fig. 3A, B) and ML-339 (Supplementary Fig. 3C, D) treatments effectively abrogated the effects observed with CM-sg ARID1A treatment, thereby blocking activation of the CXCL16/CXCR6 axis (Fig. 3E and Supplementary Fig. 3E).

To evaluate the role of CXCL16 in vivo, we silenced its expression in ARID1A-deficient EC cells using lentiviral shRNA vectors (Fig. 3F), and their migratory capacity was also evaluated in vitro (Supplementary Fig. 3F). Subcutaneous tumors were generated from wild-type, ARID1A-deficient, and ARID1A-deficient MFE-296 cells co-infected with shCXCL16. Once tumors were established, MFE-296 wild-type ARID1A cells expressing the luciferase reporter gene (MFE-296 EV-LUC) were injected retro-orbitally. Two to three weeks later, mice bearing ARID1A-deficient tumors developed lung metastases, whereas co-infection with shCXCL16 significantly reduced metastatic foci (Fig. 3G). To further validate these findings, we reproduced the in vivo experiment following the same protocol described for Fig. 3G but replaced CXCL16 silencing with pharmacological inhibition of CXCR6 using the selective antagonist ML339. This treatment significantly reduced the incidence of lung metastatic foci, recapitulating the effects observed upon CXCL16 knockdown (Supplementary Fig. 3G).

Collectively, these findings highlight the critical role of CXCL16, secreted by ARID1A-deficient EC cells, in promoting tumor dissemination in endometrial cancer.

### ARID1A loss induces activation of CXCL16/CXCR6 axis in human EC

**Fig. 4:**
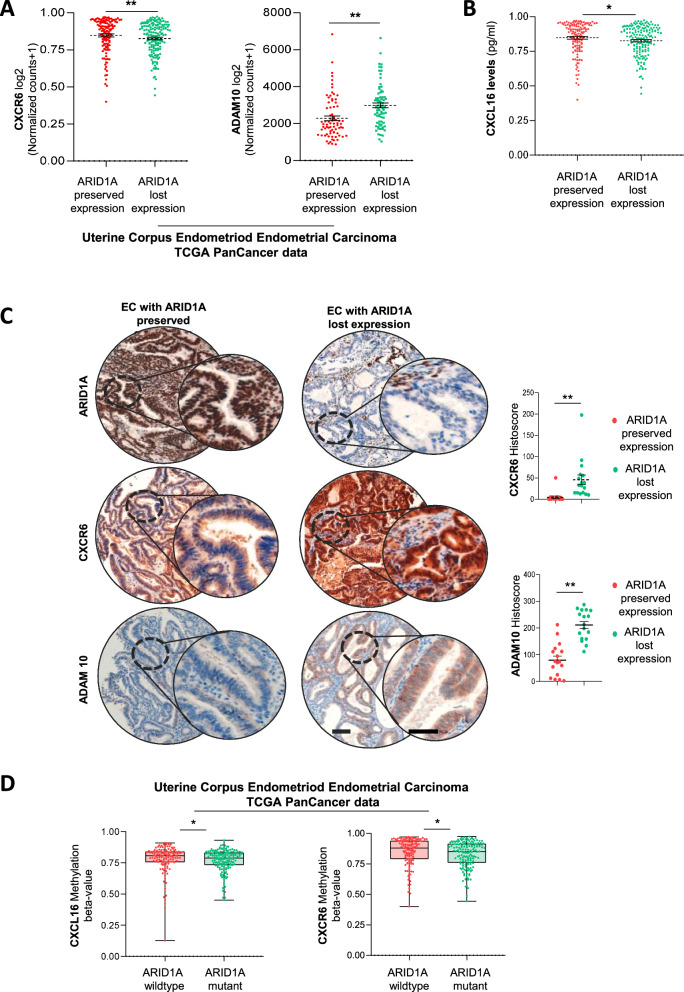
CXCL16/CXCR6 axis is upregulated in ARID1A-deficient human endometrial tumors. **A** mRNA expression levels of CXCR6 and ADAM10 in ARID1A-preserved (*n* = 76) versus ARID1A-loss (*n* = 77) tumors from the TCGA_UCEC dataset [25]. Statistical significance was assessed by the Wilcoxon rank-sum test. **B** CXCL16 protein levels were quantified by ELISA in uterine aspirates collected from patients with endometrioid endometrial cancer, stratified according to ARID1A expression status (ARID1A-preserved, *n* = 17; ARID1A-loss, *n* = 15). Statistical significance was assessed by the Wilcoxon rank-sum test. **C** Representative images and quantification of ARID1A, CXCR6 and ADAM10 immunohistochemistry in primary human EC tissue samples with preserved (*n* = 17) or lost (*n* = 17) ARID1A expression. Scale bars: 250 μm. Statistical significance was assessed by the Wilcoxon rank-sum test. **D** CXCL16 and CXCR6 DNA methylation levels (β-values) in ARID1A-wildtype (*n* = 131) and ARID1A-mutant (*n* = 162) endometrioid EC from TCGA_UCEC dataset [25]. ARID1A mutations were associated with significantly lower methylation levels in both genes. Statistical significance was assessed by the Wilcoxon rank-sum test. All graphs represent mean ± S.E.M *p < 0.05; **p < 0.01.

Based on our findings, we next analyzed the expression of key components of the CXCL16/CXCR6 axis in human EC samples. We first examined the mRNA abundance of the *CXCR6* receptor and *ADAM10* in the public TCGA_uterine corpus endometrial cancer (UCEC) dataset [25], comparing endometrioid tumors with preserved ARID1A expression (*n* = 76) to those with loss of ARID1A expression (*n* = 77). The analysis revealed that tumors with ARID1A loss exhibited significantly higher levels of *CXCR6* and *ADAM10* expression (Fig. 4A). Next, we assessed CXCL16 levels in a prospective cohort of uterine aspirates from endometrioid EC patients, stratified by ARID1A expression status. Notably, aspirates from patients with ARID1A loss (*n* = 15) exhibited significantly higher CXCL16 levels compared to those with preserved ARID1A expression (*n* = 17) (Fig. 4B). Additionally, we performed immunostaining for CXCR6 and ADAM10 in primary EC tumors. Consistently, samples with ARID1A loss demonstrated significantly increased levels of CXCR6 and ADAM10 expression compared to samples with preserved ARID1A (Fig. 4C). Clinicopathological characteristics of the cohort are summarized in Table 1. Alterations in gene expression are often influenced by epigenetic mechanisms, particularly DNA methylation, which can be affected by chromatin remodeling defects. In this context, previous studies have reported that ARID1A loss is associated with differential methylation patterns across multiple genes in gynecological cancers [26, 27]. We therefore explored whether such epigenetic regulation might contribute to the observed upregulation of CXCL16 and CXCR6 in ARID1A-mutant EC. To this end, we analyzed the DNA methylation status of these genes in endometrioid tumors from the TCGA-UCEC cohort [25]. Strikingly, tumors harboring ARID1A mutations exhibited significantly lower methylation levels in CpG islands associated with both CXCL16 and CXCR6, indicating that ARID1A loss may contribute to CXCL16 and CXCR6 upregulation through promoter hypomethylation (Fig. 4D).

**Table 1:** Clinicopathological characteristics of endometrioid EC patients stratified by ARID1A expression status.

	ARID1A Conserved Expression	ARID1A Loss Expression
	**(n** = **17)**	**(n** = **17)**
Age, Mean (±sd)	61.1 (±13.9)	58 (±10.98)
**Grade – n (%)**		
1	8 (47%)	8 (47%)
2	6 (35,3%)	7 (41,2%)
3	3 (17,7%)	2 (11,8%)
**Figo – n (%**)		
IA	10 (58.8%)	7 (41.2%)
IB	4 (23.5%)	5 (29.4%)
II	2 (11.8%)	2 (11.8%)
III	1 (5.9%)	3 (17.6%)

Summary of age, tumor grade, and FIGO stage in endometrioid EC patients with preserved (ARID1A-wild-type, *n* = 17) or lost ARID1A expression (ARID1A-deficient, *n* = 17). Age is reported as mean ± standard deviation. Grade and FIGO stage are expressed as number of cases and corresponding percentages (%).

### Conversion of stromal endometrial cells to CAFs driven by ARID1A-deficient secretome

The cancer secretome recruits and activates stromal cells and fibroblasts, forming heterogeneous, tumor-promoting CAF populations [28]. Moreover, the expression of CXCL16 within the tumor niche has been reported to further promote the conversion of stromal cells into CAFs [29].

**Fig. 5:**
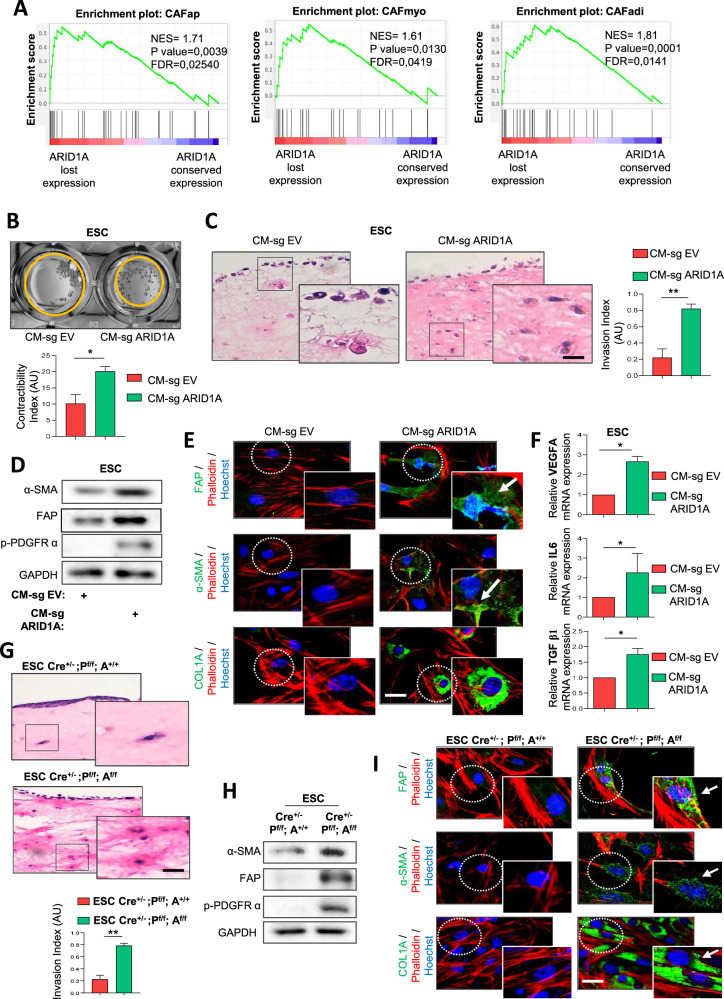
ARID1A-deficient EC cells induce activation of endometrial stromal cells and promote CAF-like phenotypes. **A** Gene set enrichment analysis was used to uncover signatures associated to CAF subtype gene signatures defined using the top 30 genes for each subtype described by [30] in EC from the TCGA_UCEC cohort [25], stratified by ARID1A expression. **B** Representative images and quantification of collagen gel contraction assay performed over 6–7 days on mouse endometrial stromal cells (ESCs) pre-treated for 48 h with conditioned media from control IK cells (CM-sg EV) or ARID1A-deficient IK cells (CM-sg ARID1A). **C** Representative images and quantification of organotypic culture assay performed over 6–7 days, showing tumor cell invasion into collagen matrices previously remodeled by ESCs pre-treated for 48 h with conditioned media from control IK cells (CM-sg EV) or ARID1A-deficient IK cells (CM-sg ARID1A). Scale bars: 50 μm. **D** Western blot analysis of CAF activation markers FAP, α-SMA and p-PDGFRα in mouse ESCs treated for 48 h with conditioned media from control IK cells (CM-sg EV) or ARID1A-deficient IK cells (CM-sg ARID1A). GAPDH was used as loading control. **E** Representative images of immunofluorescence against α-SMA, FAP and COL1A in mouse ESCs treated with CM-sg EV or CM-sg ARID1A for 48 h. Scale bars: 25 μm. **F** RT-qPCR analysis of VEGFA, IL6 and TGF-β1 mRNA expression in mouse ESCs following 48 h treatment with conditioned media derived from control or ARID1A-deficient IK cells (CM-sg EV and CM-sg ARID1A). **G** Representative images and quantification of organotypic culture assay performed over 6–7 days, showing tumor cell invasion into collagen matrices previously remodeled by ESCs isolated from *Cre*^*+/-*^*; Pten*^*f/f*^*; Arid1a*^*+/*^ and *Cre*^*+/-*^*; Pten*^*f/f*^*; Arid1a*^*f/f*^ mice. Scale bars: 50 μm. **H** Representative immunoblots of CAF activation markers α-SMA, FAP and p-PDGFRα in endometrial stromal cells isolated from *Cre*^*+/-*^*;Pten*^*f/f*^*; Arid1a*^*+/*^ and *Cre*^*+/-*^*;Pten*^*f/f*^*; Arid1a*^*f/f*^ mice. GAPDH was used as loading control. **I** Immunofluorescence analysis of α-SMA, FAP and COL1A in ESC derived from *Cre*^*+/-*^*;Pten*^*f/f*^*; Arid1a*^*+/*^ and *Cre*^*+/-*^*;Pten*^*f/f*^*; Arid1a*^*f/f*^ mice. Representative images shown. Scale bars: 25μm. N = 9 per group. Statistical significance in in vitro experiments involving ESC cells under the indicated treatment conditions was assessed using an unpaired two-tailed Student’s t-test. All graphs represent mean ± S.E.M., Student’s t-test and statistical significance is indicated as *p < 0.05; **p < 0.01. Results are representative of three independent experiments with three technical replicates per experiment.

To gain insight into CAF activation in ARID1A-deficient EC, we focused on the molecular classification of CAF subtypes: myofibroblastic (CAFmyo), adipogenic (CAFadi), inflammatory (CAFinfla), endothelial-to-mesenchymal (CAFendMT), peripheral nerve-like (CAFpn), and antigen-presenting (CAFap) [6, 30]. We applied this classification to bulk RNA-seq data from TCGA_UCEC EEC tumors [25], using the top 30 genes defining each CAF subtype [30]. The analysis revealed that EEC with ARID1A low expression were positively correlated with the CAFap, CAFmyo and CAFadi signatures, which are indicative of CAF activation (Fig. 5A).

To assess whether the secretome of ARID1A-deficient cells induces activation of endometrial stromal cells (ESCs), we isolated ESCs from wild-type mouse uteri (Supplementary Fig. 4A) and treated them with CM from ARID1A-deficient EC cells. ESCs exposed to CM-sg ARID1A adopted an elongated morphology compared to those treated with CM-sg EV (Supplementary Fig. 4B). Furthermore, collagen gel contraction assays demonstrated that ESCs treated with CM-sg ARID1A displayed a significantly higher contractility index, enabling them to contract collagen gels more effectively than their counterparts treated with CM-sg EV (Fig. 5B). Similarly, organotypic culture assays showed that matrices generated by ESCs previously treated with CM-sg ARID1A were more permissive to tumor cell invasion than those formed by ESCs treated with CM-sg EV (Fig. 5C).

Based on these results, we next aimed to evaluate the expression of several key markers linked to stromal cell activation and CAF phenotypes [31]. We observed that ESCs treated with CM-sg ARID1A showed significantly increased protein expression levels of α-SMA, fibroblast activation protein alpha (FAP), phospho-platelet-derived growth factor receptor alpha (p-PDGFRα), and collagen type I alpha chain (COL1A) (Fig. 5D, E). Beyond these markers, CAFs are also characterized by their production of vascular endothelial growth factor (VEGF) and cytokines, such as IL-6 and TGF-β [32]. Consequently, we assessed the transcriptional expression levels of these proteins and found that ESCs treated with CM-sg ARID1A exhibited significantly higher expression levels of these factors compared to those treated with CM-sg EV (Fig. 5F).

To validate these findings in an independent ARID1A-deficient EC model, we examined stromal cell activation in *Cre:ER*^*T*^*; Pten*^*f/f*^*; Arid1a*^*f/f*^ model. ESCs were isolated from *Cre:ER*^*T*^*; Pten*^*f/f*^*; Arid1a*^*f/f*^ (Cre^+/-^; P^f/f^; A^f/f^), and *Cre:ER*^*T*^*; Pten*^*f/f*^*; Arid1a*^*+/+*^ (Cre^+/-^; P^f/f^; A^+/+^) uteri. Organotypic assays performed to assess collagen matrix remodeling (Supplementary Fig. 4C) and tumor cell invasiveness within ESC-remodeled matrices (Fig. 5G) showed that matrices from ESC Cre^+/-^; P^f/f^; A^f/f^ had greater contractility and tumor cell permeability, with significantly higher invasion indices than those from controls.

In addition, we analyzed the protein expression levels of several key markers of CAFs, including α-SMA, FAP, p- PDGFRα, and COL1A (Fig. 5H, I), as well as the transcriptional levels of VEGF, IL-6, and TGF-β (Supplementary Fig. 4D). This comprehensive analysis revealed a significant increase in both protein and transcriptional expression of these markers, indicating robust stromal cell activation and a pronounced CAF phenotype in the *Cre:ER*^*T*^*; Pten*^*f/f*^*; Arid1a*^*f/f*^ stromal cells.

### ARID1A deficiency induces CAF activation in vivo

**Fig. 6:**
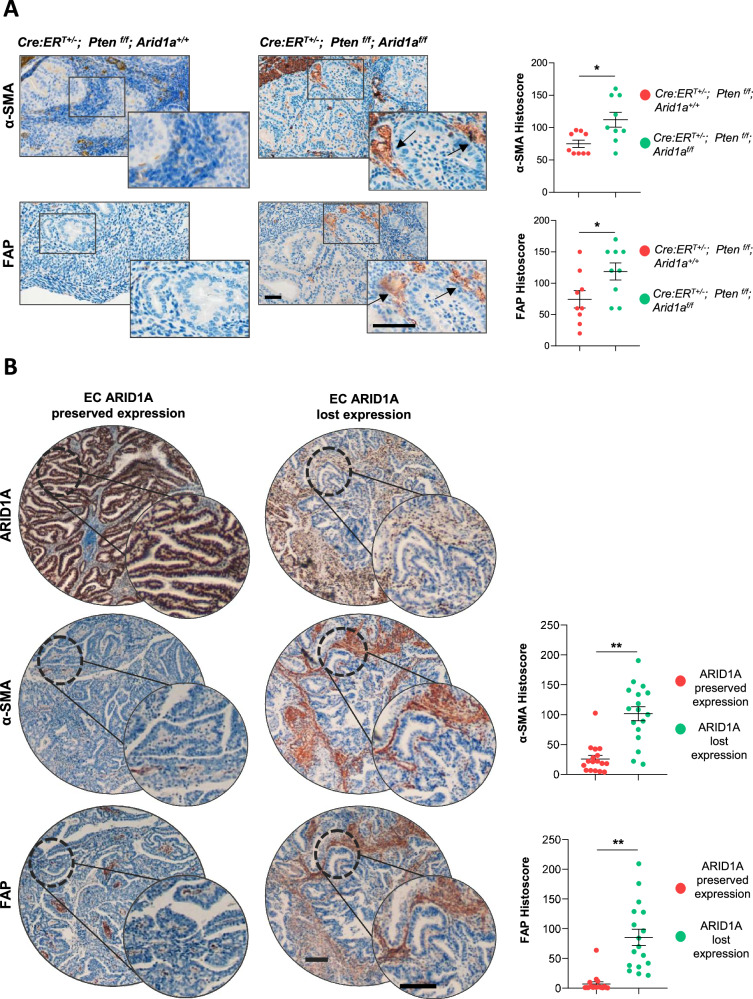
ARID1A loss promotes CAF activation in murine and human endometrial tumors. **A** Representative images and quantification of α-SMA and FAP immunohistochemistry in endometrial tumors from female *Cre*^*+/-*^*; Pten*^*f/f*^*; Arid1a*^*+/*^ (*n* = 9) and *Cre*^*+/-*^*; Pten*^*f/f*^*; Arid1a*^*f/f*^ (*n* = 9) mice. Scale bars: 250 μm. Statistical significance was assessed by the Wilcoxon rank-sum test. **B** Representative images and quantification of ARID1A, α-SMA and FAP immunohistochemistry in human endometrial tumors stratified by ARID1A expression status (ARID1A-preserved, *n* = 17; ARID1A-loss, *n* = 17). Scale bars: 250 μm. Statistical significance was assessed by the Wilcoxon rank-sum test. All graphs represent mean ± S.E.M., and statistical significance is indicated as Student’s t-test *p < 0.05; **p < 0.01.

To determine whether ARID1A loss in EC promotes stromal reprogramming and CAF activation, we performed immunohistochemical staining for FAP and α-SMA in the *Cre:ER*^*T*^*; Pten*^*f/f*^*; Arid1a*^*f/f*^ (Cre^+/-^; P^f/f^; A^f/f^) murine model and its *Cre:ER*^*T*^*; Pten*^*f/f*^*; Arid1a*^*+/+*^ (Cre^+/-^; P^f/f^; A^+/+^) control. The results demonstrated that uteri from ARID1A-deficient animals exhibited significantly higher α-SMA and FAP expression in the stromal compartment compared with controls (Fig. 6A). To determine whether these changes also occur in human endometrial carcinoma, we analyzed α-SMA and FAP expression in patient samples stratified by ARID1A status. Consistent with our murine model, ARID1A-deficient tumors displayed increased expression of both markers (Fig. 6B). These findings provide compelling evidence that ARID1A loss remodels the TME, driving CAF activation in EC.

### Chemokine CXCL16 induces CAF activation in EC

**Fig. 7:**
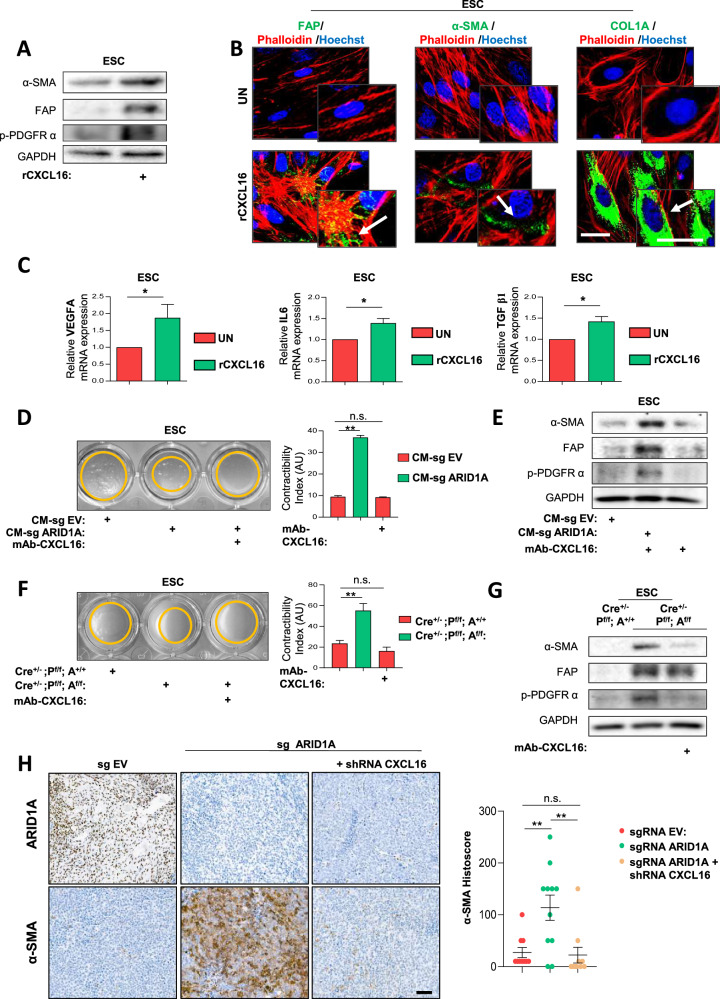
CXCL16 drives CAF activation in endometrial cancer. **A** Western blot analysis of CAF activation markers α-SMA, FAP, and p-PDGFRα in mouse ESCs treated for 48 h with recombinant rCXCL16 (100 ng/ml). GAPDH was used as loading control. **B** Representative immunofluorescence images of FAP, α-SMA, and COL-1A in mouse ESCs treated for 48 h with rCXCL16 (100 ng/ml). Arrows indicate areas of marker overexpression. Scale bar: 50 μm. **C** Transcriptional levels of VEGFA, IL6 and TGFβ1 measured by RT-qPCR in ESCs following exposure to rCXCL16 (100 ng/ml) for 48 h. Statistical significance was calculated using an unpaired two-tailed Student’s t-test. **D** Collagen gel contraction assay performed over 6-7 days on ESCs exposed to conditioned media from control IK cells (CM-sg EV) or ARID1A-deficient IK cells (CM-sg ARID1A) in the presence or absence of the mAb-CXCL16 (8 μg/ml) performed over 6–7 days. Representative images and quantification are shown. Statistical significance was assessed using one-way ANOVA followed by Bonferroni post-hoc test. **E** Western blot analysis of α-SMA, FAP, and p-PDGFRα protein expression in ESCs treated with conditioned media from control or ARID1A-deficient IK cells (CM-sg EV and CM-sg ARID1A), with or without mAb-CXCL16 (8 μg/ml). GAPDH was used as loading control. **F** Representative images and quantification of collagen gel contraction assay using ESCs pre-treated with plasma from *Cre*^*+/-*^*; Pten*^*f/f*^*; Arid1a*^*++/*^ or *Cre*^*+/-*^*; Pten*^*f/f*^*; Arid1a*^*f/f*^ mice, with or without mAb-CXCL16 (8 μg/ml). Collagen remodeling was monitored over a 6–7 day period. Statistical significance was assessed using one-way ANOVA followed by Bonferroni post-hoc test. **G** Protein expression analysis of α-SMA, FAP, and p-PDGFRα in ESCs treated with plasma from *Cre*^*+/-*^*; Pten*^*f/f*^*; Arid1a*^*++/*^ or *Cre*^*+/-*^*; Pten*^*f/f*^*; Arid1a*^*f/f*^ mice, in the presence or absence of the CXCL16-neutralizing antibody (mAb-CXCL16- 8 μg/ml), as assessed by western blot. GAPDH was used as a loading control. **H** Representative images and quantification of ARID1A and α-SMA immunohistochemistry in subcutaneous tumors generated from MFE-296 cells with ARID1A-wild-type, ARID1A-deficient, or ARID1A-deficient background combined with stable shRNA-CXCL16 knockdown (shCXCL16). Scale bars: 250μm. Statistical analysis was performed using the Kruskal-Wallis test followed by Dunn’s multiple comparisons post-hoc test (*n* = 10 per group). All graphs represent mean ± S.E.M., and statistical significance is indicated as Student’s t-test *p < 0.05; **p < 0.01; n.s. non-significant. Results are representative of three independent experiments with three technical replicates per experiment.

To determine whether CAF activation was directly mediated by CXCL16, ESCs isolated from wild-type mouse uteri were treated with rCXCL16. After 48 hours, treated cells exhibited increased contractility index and upregulated expression of α-SMA, FAP, p-PDGFRα, and COL1A (Fig. 7A, B and Supplementary Fig. 5A). RT-PCR analysis confirmed a significant transcriptional upregulation of VEGF, IL-6, and TGF-β, further supporting the role of CXCL16 in driving CAF activation (Fig. 7C).

To validate that CAF activation induced by CM-sg ARID1A or by media from Cre^+/-^; P^f/f^; A^f/f^ epithelial cells was CXCL16-dependent, ESCs were treated with these media in the presence of mAb-CXCL16. CXCL16 blockade inhibited ESC phenotypic transformation, reducing collagen gel contraction and suppressing CAF marker expression (Fig. 7D, G). Similar results were obtained when using the selective CXCR6 antagonist, ML-339 (Supplementary Fig. 5B–E).

Finally, α-SMA expression was analyzed in subcutaneous tumors derived from wild-type, ARID1A-mutant and ARID1A-mutant cells co-infected with shRNA-CXCL16. Tumors co-expressing sg ARID1A and shRNA-CXCL16 exhibited reduced α-SMA levels compared to those with sg- ARID1A and wild-type CXCL16 expression (Fig. 7H).

Collectively, these findings position CXCL16 as a key driver of CAF activation in EC, highlighting the CXCL16/CXCR6 axis as a potential therapeutic target for modulating stromal reprogramming.

## Discussion

The incorporation of genomic characterization into clinical practice has significantly advanced early risk stratification in EC [25]. However, despite these advances, therapeutic recommendations remain undefined for more than 50% of newly diagnosed EC cases [33].

ARID1A alterations are present in approximately one-third of EC cases across various molecular subtypes [33–35]. As a core component of the SWI/SNF chromatin remodeling complex, ARID1A plays a critical role in maintaining endometrial epithelial identity by repressing mesenchymal differentiation. Its loss causes epigenetic dysregulation, including DNA methylation changes, leading to dedifferentiation and tumor progression [13, 15, 26]. Furthermore, several studies have independently correlated ARID1A mutations with increased recurrence and poorer progression-free survival in EC patients [36], positioning its genomic inactivation as a molecular driver of dedifferentiation and EC aggressiveness [37].

In the current study, we demonstrate that ARID1A-deficient EC cells secrete soluble factors, notably CXCL16, into the local microenvironment and the bloodstream. This secretion enhances the aggressiveness of adjacent ARID1A-wild-type EC cells and drives CAF activation in stromal cells, collectively shaping a pro-metastatic TME.

CXCL16, a chemotactic α-chemokine regulated by ARID family members (as shown for CXCL14 [38]), is a plasma membrane-associated molecule composed of a chemokine domain, a glycosylated mucin-like stalk, a single transmembrane helix, and a short cytoplasmic tail [39]. Its soluble form, generated through ADAM10-mediated ectodomain shedding [40], promotes the migration of CXCR6-expressing cells, while the transmembrane isoform acts as a scavenger receptor for oxidized low-density lipoproteins and facilitates adhesion to CXCR6-positive cells [41].

CXCL16 has been implicated in cancer progression and a variety of non-neoplastic diseases, including renal fibrosis or non-alcoholic fatty liver disease [42]. In gastric cancer, its overexpression enhances invasiveness via ADAM10-dependent cleavage, activating downstream Akt and MAPK signaling pathways [43]. Similarly, CXCL16/CXCR6 axis activation drives EMT and metastatic dissemination in breast, pancreatic, lung and nasopharyngeal cancers [44–46].

In gynecologic cancers, while the role of some chemokine networks, such as CXCL12-CXCR4 axis has been extensively studied and related to poor prognosis [47–49], the role of the CXCL16/CXCR6 axis remains unexplored. Notably, this axis has been implicated in endometriosis through TNF-α induction in ectopic ESCs, promoting epithelial cell migration and invasion. In the EC context, CXCL16 dysregulation correlates with poor overall survival (OS) [50]. While the upstream regulatory mechanisms remain incompletely defined, our data suggest that reduced promoter methylation may contribute to the increased CXCL16 expression observed in ARID1A-deficient tumors. In our study, we further found that CXCL16 upregulates CXCR6 and ADAM10 in both ARID1A-deficient and neighboring ARID1A-wild-type EC cells. This signaling cascade triggers MAPK and Paxillin/FAK activation, promotes an EMT-like phenotype and enhances migration and invasion [51]. In this context, ADAM10 upregulation emerges as a downstream event in CXCL16 signaling, reinforcing its pro-invasive effects and aligning with its previously established role in pancreatic cancer invasion [52].

Our findings suggest that similar mechanisms may contribute to CXCL16-driven tumor progression in ARID1A-deficient EC. Cell polarization via actin polymerization initiates migration and metastasis [53]. FAK regulates focal adhesion dynamics and activates MAPK/ERK and YAP/TAZ signaling [54], and motility-related proteins, such as p130Cas and Paxillin [55]. Accordingly, our results indicate that CXCL16-CXCR6 activation enhances EC cell migration via the Paxillin/FAK signaling pathway, leading to YAP/TAZ activation. Comparable mechanisms have been described in prostate and breast cancer, where CXCL16 promotes cell migration via αVβ3 integrin in an ERK- and FAK-dependent manner [44, 56, 57], suggesting a conserved role for CXCL16 in tumor motility. Beyond its direct effects on tumor cells, we demonstrated that CXCL16 also contributes to TME remodeling, which is critical for tumor progression in ARID1A-deficient EC. The crosstalk between tumor-derived soluble factors and reactive stromal cells reprograms the stroma, shifting the TME from a tumor-restrictive to a tumor-supportive state that facilitates dissemination. Among these changes, the conversion of stromal endometrial cells into CAFs is particularly relevant [2]. Tumor-derived factors, including chemokines, promote CAF activation, as evidenced by upregulation of p-PDGFRα, FAP, α-SMA and COL1A [58–60].

CAFs not only play a central role in EC progression and dissemination [2] by secreting high levels of collagen I and III, contributing to desmoplasia [61], but they also secrete cytokines, such as IL-6, IL-8, monocyte chemotactic protein-1 (MCP-1), chemokine ligand 5 (RANTES), and vascular endothelial growth factor (VEGF) [62], which further facilitate tumor cell migration and invasion [2].

**Fig. 8:**
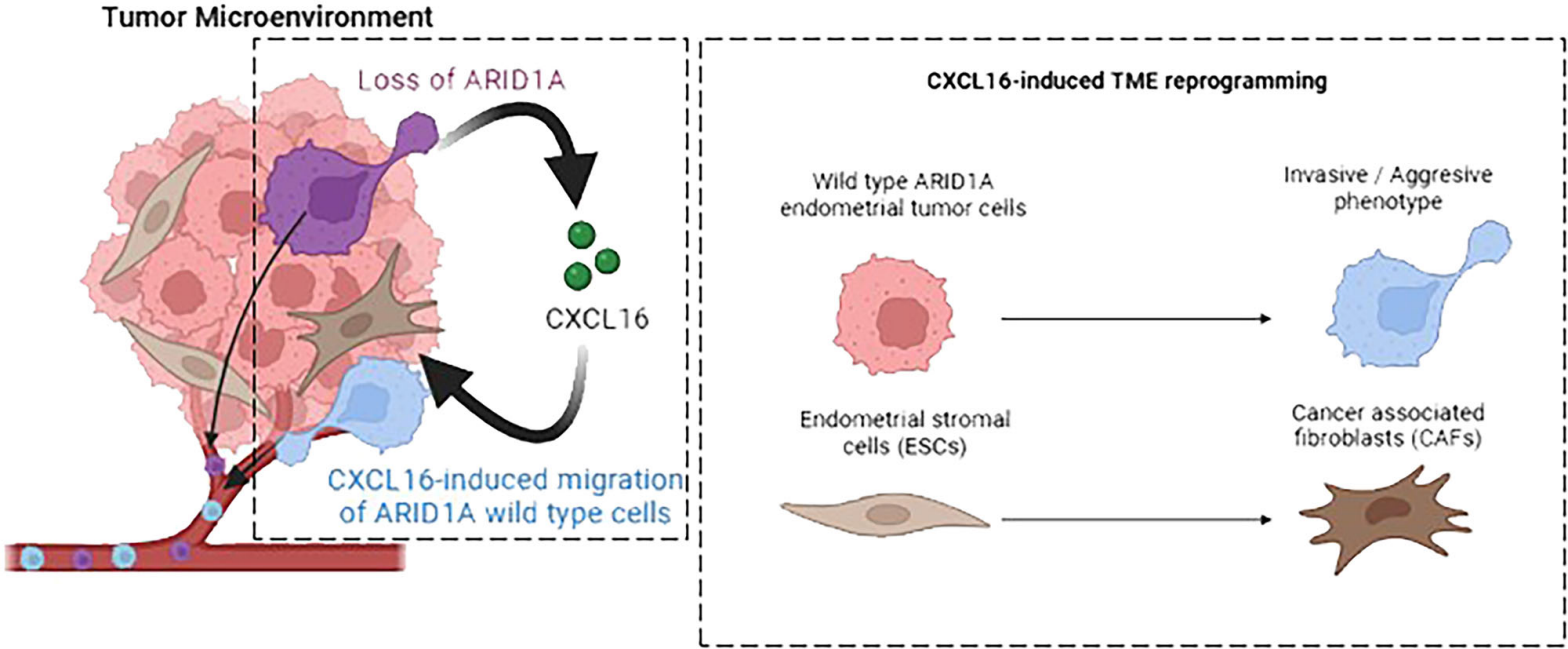
Proposed schematic model. ARID1A deficiency alters the secretome, increasing CXCL16 chemokine release, which induces changes in adjacent ARID1A-wildtype EC cells, promoting an invasive/aggressive phenotype. Furthermore, elevated CXCL16 levels activate endometrial stromal cells and drive their conversion into CAFs. These TME alterations enhance the ability of EC cells to disseminate to distant organs. Created with BioRender.com.

Our findings align with these studies, providing compelling evidence that CM from ARID1A-deficient cells and plasma from *Cre:ER*^*T*^*;Pten*^*f/f*^*; Arid1a*^*f/f*^ mice induce CAF conversion, displaying markers, such as p-PDGFRα, FAP, α-SMA, and COL1A, along with increased transcription of VEGF, IL-6, and TGF-β. This was further corroborated by FAP and α-SMA upregulation in stromal tissue from *Cre:ER*^*T*^*; Pten*^*f/f*^*; Arid1a*^*f/f*^ mice and in human EC samples with ARID1A loss. We identified CXCL16 as the main driver of CAF activation in ARID1A-deficient EC. This extensive TME remodeling likely contributes to the enhanced tumor invasiveness and metastatic potential observed in our models (Fig. 8).

CXCL16 also plays a pivotal role in CAF activation in prostate cancer, where CAFs secrete elevated CXCL12 and induce EMT in tumor cells, facilitating metastasis [29]. Moreover, in breast cancer models, the CXCL16/CXCR6 axis regulates angiogenesis in the TME, promoting chronic hypoxia and supporting capillary formation and metastasis in BRAF V600E mutant colorectal cancer [63, 64]. In the context of brain metastasis, elevated CXCL16 expression in the brain metastatic niche promotes the migration of patient-derived breast cancer cells, driving brain metastatic foci formation [65]. Given the increased CXCL16 levels observed in the uterine aspirates of EC patients with ARID1A loss of expression and in plasma from ARID1A-deficient EC mouse model, along with its role in stromal remodeling at the primary site, it is plausible that CXCL16 also shapes the microenvironment of EC metastatic sites.

Considering the central role of CXCL16 in tumor progression, TME remodeling, and therapy resistance, the CXCL16-CXCR6 axis represents a promising therapeutic target. Its activation not only drives tumor progression and reshapes the TME but has also been linked to resistance to cancer therapies. In prostate cancer [66], its activation is associated with therapy resistance, while in ovarian cancer, it has been linked to reduced chemotherapy efficacy [67]. In non-small cell lung cancer, lower CXCL16-CXCR6 axis activation correlates with improved overall survival following bevacizumab-containing chemotherapy [68]. Likewise, in triple-negative breast cancer, CXCL16 neutralization reduces PD-1 high and PD-L1 high immune cell infiltration, enhancing response to anti-PD-1 therapy [69]. In our model, blockade of this axis, through neutralizing antibodies, CXCR6 antagonists, or genetic ablation, effectively disrupted migratory and invasive programs and significantly reduced lung metastases in vivo, supporting its therapeutic relevance in EC.

Beyond its direct effects on tumor cells, CXCL16 promotes a pro-tumorigenic microenvironment by inducing CAF activation and enabling a metastasis-permissive niche. Targeting CXCL16 may serve as an adjuvant strategy to modulate the TME and enhance current therapies, particularly in metastatic EC, where outcomes remain poor. Although stromal-targeted therapies show promise, clinical implementation requires well-designed trials, including neoadjuvant approaches, to optimize efficacy. In this context, CXCL16-CXCR6 inhibitors provide a rationale to improve patient outcomes by disrupting tumor-stroma interactions and limiting metastatic progression.

In conclusion, deciphering secretome alterations within the TME and their role in mediating communication between aggressive ARID1A-deficient EC tumors and surrounding stromal cells identifies CXCL16 as a key regulator of tumor behavior and CAF activation. Targeting the CXCL16-CXCR6 axis may represent a novel therapeutic strategy in EC, warranting further clinical validation and exploration of combinatorial approaches to enhance CXCL16-CXCR6 blockade efficacy.

## Supplementary information

CDDIS-25-4135_final_version_Supplementary_text

Raw Data Western Blot

## Data Availability

The datasets generated and/or analyzed in this study can be obtained from the corresponding author upon reasonable request.
