# Supplementary material for: ARID1A deficiency reprograms the tumor secretome, enhancing microenvironmental remodeling and metastatic dissemination in endometrial carcinoma
Source: Cell Death Dis. 2026 Apr 10;17(1):488. doi: 10.1038/s41419-026-08723-z (PMC13186946; doi:10.1038/s41419-026-08723-z)
Supplement: Supplementary file 1 — CDDIS-25-4135_final_version_Supplementary_text [file 41419_2026_8723_MOESM1_ESM.docx]

***ARID1A* deficiency reprograms the tumor secretome, enhancing microenvironmental remodeling and metastatic dissemination in endometrial carcinoma.**

Cristina Megino-Luque^1,2,3^*, Manel Albertí-Valls^1^, Sara Olave^1,3^, Pol Sisó^4^, Núria Bonifaci^3,5^, Anna Macià^4^, Xavier Matias-Guiu^3,6,7^, Sònia Gatius^3,6,7^, David Llobet-Navas^3,5^ and Núria Eritja^1,3,^*

SUPPLEMENTARY FIGURE LEGENDS

**Figure Supp 1.**

**A**) Wound-healing assay in MFE-296 cells treated with conditioned media from control (CM-sg EV) or ARID1A-deficient (CM-sg ARID1A) EC cells. Representative images at 0, 4 and 8 hours are shown, along with quantification of wound closure area. Scale bars: 50μm. **B)** Transwell invasion assay performed in MFE-296 cells treated for 48 hours with conditioned media from CM-sg EV or CM-sg ARID1A. Representative Hoechst-stained images (top) and corresponding quantification of Matrigel®-invading cells (bottom) are shown. Scale bars: 50μm. **C)** Western blot analysis of protein expression levels of ARID1A, E-cadherin, β-catenin, N-cadherin, vimentin, SNAIL, and ZEB1 in MFE-296 cells treated with CM-sg EV or CM-sg ARID1A. GAPDH was used as loading control. **D**) Immunohistochemistry for PTEN and ARID1A in endometrial tumors from Cre^+/-^; Pten^f/f^; Arid1a^+/+^ and Cre^+/-^; Pten^f/f^; Arid1a^f/f^ mice, confirming loss of expression in the double-deletion model. Scale bars: 250μm. **E**) Wound-healing assay in MFE-296 cells treated with plasma from Cre^+/-^; Pten^f/f^; Arid1a^+/+^ or Cre^+/-^; Pten^f/f^; Arid1a^f/f^ mice. Representative images at 0 and 48 hours are shown, together with quantification of wound closure area. **F**) Transwell invasion assay in MFE-296 cells treated with plasma from Cre^+/-^; Pten^f/f^; Arid1a^+/+^ or Cre^+/-^; Pten^f/f^; Arid1a^f/f^ mice. Representative images of Hoechst-stained nuclei (left) and corresponding quantification of invading cells (right) are shown. Scale bars: 50 μm. **G**) Western blot analysis of protein expression levels of E-cadherin, β-catenin, N-cadherin, Vimentin, SNAIL, and ZEB1 in MFE-296 cells treated with plasma from Cre^+/-^; Pten^f/f^; Arid1a^+/+^ or Cre^+/-^; Pten^f/f^; Arid1a^f/f^ mice. GAPDH was used as loading control. Statistical significance in *in vitro* experiments involving endometrial cancer cell lines under the indicated conditions was assessed using unpaired two-tailed Student’s t-test.

All graphs represent mean ± S.E.M. Statistical significance is indicated as * p < 0.05; ** p < 0.01. Student’s t-test. Results are representative of three independent experiments with three technical replicates per experiment.

**Figure Supp 2.**

**A)** Chemoarray semiquantification results of CXCL16 levels in conditioned media from IK cells with preserved (sgRNA EV) or disrupted (sgRNA ARID1A) ARID1A expression. Statistical significance was assessed using one-way ANOVA followed by Bonferroni post-hoc test. **B)** CXCL16 protein levels measured by ELISA in MFE-296 cells treated with CM-sg EV or CM-sg ARID1A. Statistical significance was calculated using an unpaired two-tailed Student’s t-test. **C**) RT-qPCR analysis of CXCL16, CXCR6, and ADAM10 mRNA expression in IK and MFE-296 cells with preserved or disrupted ARID1A expression, and in wild-type cells treated with CM-sg ARID1A for 48 hours. Statistical significance in EC cell lines under the indicated treatment conditions was assessed using One-way ANOVA followed by Bonferroni post-hoc test. **D)** Western blot analysis in IK cells treated with plasma from Cre^+/-^; Pten^f/f^; Arid1a^+/+^ or Cre^+/-^; Ptenf^/f^; Arid1a^f/f^ mice, showing protein expression levels of key components of the CXCL16/CXCR6 signaling axis and its downstream pathways, including CXCL16, CXCR6, ADAM10, MMP2, MMP9, p-ERK1/2, pan-ERK1/2, p-FAK, p-Paxillin and YAP. GAPDH was used as loading control. **E**) Immunofluorescence analysis in IK cells with disrupted ARID1A expression or treated with CM-sgARID1A, showing nuclear localization of YAP, increased p-Paxillin accumulation at the plasma membrane, and enhanced association of p-MLY2 with actin stress fibers (phalloidin). Scale bars: 250μm.

All graphs represent mean ± S.E.M. Statistical significance is indicated as * p < 0.05; ** p < 0.01. Student’s t-test Results are representative of three independent experiments with three technical replicates per experiment.

**Figure Supp 3.**

**A**) Wound-healing assay in IK cells treated with CM-sg EV, CM-sg ARID1A, or CM-sg ARID1A combined with the mAb-CXCL16 (8 μg/ml). Representative images and quantification of wound closure area are shown. Scale bars: 200μm. **B**) Transwell invasion assay in IK cells treated with CM-sg EV, CM-sg ARID1A, or CM-sg ARID1A combined with mAb-CXCL16 (8 μg/ml). Shown are Hoechst-stained nuclei (top) and quantification of Matrigel®-invading cells (bottom). Scale bars: 50μm. **C**) Representative wound-healing images and quantification in IK cells treated with CM-sg EV, CM-sg ARID1A, or CM-sg ARID1A combined with the CXCR6 antagonist ML-339 (50μM). Scale bars: 200μm. **D**) Representative images and quantification of transwell invasion assay in IK cells exposed to CM-sg EV, CM-sg ARID1A, or CM-sg ARID1A plus the CXCR6 antagonist ML-339 (50μM). Hoechst staining highlights invading nuclei (top), with quantification shown below. Scale bars: 50μm. **E**) Western blot analysis in IK cells treated with CM-sg EV, CM-sg ARID1A, or CM-sg ARID1A combined with the CXCR6 antagonist ML-339 (50μM). Protein expression of key components of the CXCL16/CXCR6 axis and downstream MAPK, FAK/Paxillin, YAP signaling and EMT pathways was evaluated, including E-Cadherin, β-Catenin, N-cadherin, Vimentin, ADAM10, MMP2, MMP9, p-ERK1/2, pan-ERK1/2, p-FAK, p-Paxillin, and YAP. GAPDH was used as loading control. **F**) Wound-healing assay in MFE-296 cells treated with CM-sg EV, or with conditioned media from ARID1A-deficient cells co-infected with shRNA targeting CXCL16 (shRNA CXCL16). Representative images and quantification of wound closure area are shown. Scale bars: 200μm. **G**) Bilateral subcutaneous (SC) tumors were established using ARID1A–wild-type or –deficient MFE-296 cells. After two weeks, mice bearing ARID1A-deficient tumors (sgARID1A) began receiving a daily oral dose of the CXCR6 antagonist ML339 (30 mg/kg/day) for one week to prime the tumor microenvironment. Subsequently, luciferase-labelled wild-type MFE-296 cells were injected via the retro-orbital (RO) route while ML339 treatment continued (30 mg/kg/day). Two weeks later, metastatic dissemination was evaluated by in vivo bioluminescence imaging and validated by macroscopic lung inspection and H&E histopathology. Representative bioluminescent images, gross lung photographs, and H&E-stained lung sections illustrating metastatic foci are shown. The number of mice presenting lung metastases is reported (bottom right). Statistical analysis was performed using the Chi-square test for overall group comparison, followed by pairwise comparisons with Bonferroni correction, n=5 per group. Scale bars, 250 μm. Statistical significance in *in vitro* experiments involving endometrial cancer cell lines under the indicated conditions was assessed using One-way ANOVA followed by Bonferroni post-hoc test. All graphs represent mean ± S.E.M. Statistical significance is indicated as *p < 0.05; ** p < 0.01. Results are representative of three independent experiments with three technical replicates per experiment.

**Figure Supp 4.**

**A**) Western blot showing expression of epithelial markers (β-catenin and cytokeratin-7) and stromal markers (vimentin and CD10) in primary epithelial (EP) and stromal cell (ESC) populations isolated from mouse endometrium. **B**) Phase-contrast images of ESCs treated with CM-sg EV or CM-sg ARID1A. Treatment with CM-sg ARID1A induced a more elongated stromal morphology, indicative of activation. Scale bars: 50μm. **C**) Representative images and quantification of collagen gel contraction assay over 6–7 days in ESCs isolated from Cre^+/-^; Pten^f/f^; Arid1a^+/+^ or Cre^+/-^; Pten^f/f^; Arid1af/f mice. **D**) Transcriptional analysis by RT-qPCR of IL6, VEGFA, and TGFβ1 in ESCs isolated from Cre^+/-^; Pten^f/f^; Arid1a^+/+^ or Cre^+/-^; Pten^f/f^; Arid1a^f/f^ mice, evaluating markers associated with CAFs activation. Statistical significance in *in vitro* experiments involving ESC cells under the indicated treatment conditions was assessed using an unpaired two-tailed Student’s t-test.

All graphs represent mean ± S.E.M., Student’s t-test and statistical significance is indicated as *p < 0.05; **p < 0.01. Results are representative of three independent experiments with three technical replicates per experiment.

**Figure Supp 5.**

**A**) Representative images and quantification of collagen gel contraction over 6–7 days in ESCs treated with rCXCL16 (100ng/ml) or vehicle control. **B**) Collagen gel contraction in ESCs treated with CM-sg EV, CM-sg ARID1A, or CM-sg ARID1A plus the CXCR6 antagonist ML-339 (50μM), monitored over 6–7 days. Representative images and quantification are shown. **C**) Expression of CAF activation markers (α-SMA, FAP, p-PDGFRα) assessed by western blot in ESCs exposed to CM-sg EV, CM-sg ARID1A, or CM-sg ARID1A combined with the CXCR6 antagonist ML-339 (50μM). GAPDH was used as loading control. **D**) Collagen gel contraction in ESCs treated with plasma from Cre^+/-^; Pten^f/f^; Arid1a^+/+^ or Cre^+/-^; Pten^f/f^; Arid1af/f mice, with ML-339 (50μM) added to the ARID1A-deficient condition. Representative images and quantification are shown. **E**) Expression of α-SMA, FAP, and p-PDGFRα in ESCs treated with plasma from Cre^+/-^; Pten^f/f^; Arid1a^+/+^ or Cre^+/-^; Pten^f/f^; Arid1a^f/f^ mice, assessed by western blot. ML-339 (50μM) was included in the ARID1A-deficient condition. GAPDH served as a loading control. Statistical significance in *in vitro* experiments involving ESC cells under the indicated treatment conditions was assessed using an unpaired two-tailed Student’s t-test for panel A, and using One-way ANOVA followed by Bonferroni post-hoc test for panels B and D.

All graphs represent mean ± S.E.M. Statistical significance is indicated as *p < 0.05; *p < 0.01*.* Results are representative of three independent experiments with three technical replicates per experiment.

SUPPLEMENTARY INFORMATION

MATERIAL AND METHODS

**Reagents and antibodies for western blot**

The following reagents were used: anti-GAPDH (Abcam 8245); anti-ARID1A (Cell Signaling,12354); anti-E-cadherin (BD Biosciences, 610181); anti-β-catenin (BD Biosciences, 610153); anti-N-cadherin (Santa Cruz, sc-7939); anti-Vimentin (BD bioscience, 550513); anti-SNAIL (Cell Signaling, 3879); anti-ZEB1 (Santa Cruz, sc-515797); anti-α-SMA (Santa Cruz, sc-53015); anti-FAP (Santa Cruz, sc-100528); anti-p-PDGFRa (Tyr754) (Santa Cruz, sc-12911); anti-CXCL16 (Santa Cruz, sc-514363); anti-CXCR6 (Abcam, ab125115); anti-ADAM10 (Merkel, AB19026); anti-p-paxillin (Tyr118) (Cell signaling, 2541); phosphor-myosin light chain 2 (Ser19) (Cell signaling, 3671); anti-MMP9 (Santa Cruz, sc-21733); anti-MMP2 (Santa Cruz, sc-13594); anti-p-ERK1/2 (Biolegend, 675502); anti-pan-ERK1/2 (Sigma-Aldrich, 610623); anti-p-FAK (Thermo Fisher, 700255); anti-YAP (Santa Cruz, sc-101199); anti-CD10 (Thermo Fisher, PA5-47075); anti-cytokeratin-7 (Thermo Fisher, MA1-06315); recombinant CXCL16 (PeproTech, #300-55); ML-339 (R&D Systems, HY-5943); mAb-CXCL16 (Thermo Fisher, 256213).

**Cell culture**

Ishikawa 3-H-12 (IK) were purchased from Sigma-Aldrich (Sigma-Aldrich, 99040201), as well as MFE-296 cell line (Sigma-Aldrich, 98031101). Cells were grown in Dulbecco’s modified Eagle’s medium (DMEM; Sigma-Aldrich, 12007559) supplemented with 10% fetal bovine serum (FBS; Invitrogen, 10270106), 1 mmol/L HEPES (Sigma-Aldrich, H0887), 1 mmol/L sodium pyruvate (ThermoFisher, 11360039), 2 mmol/L L-glutamine (Sigma-Aldrich, C59202), 1% penicillin/streptomycin (Sigma-Aldrich, P4333) at 37C with saturating humidity and 5% CO2.

To generate Ishikawa 3-H-12 (IK) and MFE-296 sgRNA *ARID1A-*deficient cell lines, cells were infected whit the lentiviral plasmid encoding Cas9 and the sgRNA against *ARID1A*(1). Cells infected with viruses encoding the puromycin resistance gene were selected in 2 μg/ml puromycin. All cell lines were regularly tested for mycoplasma contamination to ensure culture integrity.

**Humans Samples**

Uterine Aspirates and formalin-fixed paraffin-embedded samples were obtained from 34 patients diagnosed with endometrioid EC who underwent primary surgical resection at Hospital Universitari Arnau de Vilanova de Lleida (HUAV). Clinical information was retrieved for each patient (**Table 1**). The study was approved by the clinical research ethics committee of HUAV (study registry 03/2021), and all participants provided written informed consent in accordance with the Declaration of Helsinki.

**Isolation of mouse endometrial epithelial cells and ESC**

Isolation of mouse endometrial epithelial and stromal cells (ESC) was performed as described previously with minor modifications^19^. Briefly, after mice sacrifice uterus was dissected and washed with Hanks’ balanced salt solution (Invitrogen/14175-046) and chopped in 3- to 4-mm-length fragments. Uterine fragments were digested with 1% trypsin-EDTA solution (Sigma-Aldrich, T4049) in Hanks’ balanced salt solution for 1 hour at 4°C and 45 minutes at room temperature. Trypsin digestion was stopped by addition of Dulbecco’s modified Eagle’s medium (DMEM) containing 10% fetal bovine serum (Invitrogen). After trypsin digestion, epithelial sheets were squeezed out of the uterine pieces and separated from the stroma by applying gentle pressure with the edge of a razor blade. Epithelial sheets were washed twice with PBS and resuspended in 1 ml of DMEM/F12 (Sigma-Aldrich, 11580376) supplemented with 1 mmol/L HEPES (Sigma-Aldrich), 1% penicillin/ streptomycin (Sigma-Aldrich), and Fungizone (Invitrogen/15290026). On the other hand, for endometrial stromal cells isolation, the stromal uterine pieces were cut with a scalpel blade into smaller fragments, washed with 1X PBS and centrifuged at 1000 rpm for 3 min. These fragments were then disrupted with a 1% collagenase IA solution (200mg/ml, Worthington Biochemical Corporation, CLS-1) in DMEM culture medium supplemented with FBS, and incubated for 2h at 37ºC with agitation at 700rpm. After this time the solution was filtered with a 40µM Steril Cell Strainer (Fisher scientific, 11587522) and the isolated stromal cells were seeded in a p100 plate with stromal cell medium composed of DMEM/F12 supplemented with 4% FBS, 2 mmol/L sodium pyruvate, 1% penicillin/streptomycin and 0.5% Insulin-Transferrin-Sodium Selenite (ThermoFisher scientific, 41400045). After 24 h, the cells were washed with 1X PBS and the medium was changed to fresh medium. The cells were incubated at 37°C with saturated humidity and 5% CO2.

**Conditioned media (CM) collection**

For the CMs collection, cells were seeded under standard conditions until reaching 70% confluence. At this point, they were washed with 1X PBS and incubated with fresh serum-starved medium for further collection. After 48h the CMs were collected and centrifuged at 10000 rpm for 5 min. These CMs were recovered and passed through a 0.22µM filter and centrifuged at 120,000 x g, 18 hours, 4˚C in a Beckman Coulter Optima XPN to eliminate extracellular vesicles(2). Finally, the CMs were aliquoted and stored at -80ºC until use.

**3D spheroids co-cultures system of EC cell lines**

Growth of human endometrial epithelial cell lines in cultures was performed as described previously with minor modifications(3). Briefly, cells were washed with Hanks Balanced Salt Solution HBSS and incubated with trypsin-EDTA solution for 3 min at 37Cº. Trypsin activity was stopped by adding DMEM containing 10% FBS. Cells were centrifuged at 18 x g for 3 min. First, about 1,000 endometrial epithelial cells or endometrial stromal cells (those desired for each condition) were resuspended in 10 µl of Matrigel (BD Biosciences, 354234) per well of M96, generating a base that was allowed to polymerize at 37°C for 30 min. After this time, 1,500 endometrial GFP-labelled epithelial cells/well were resuspended in 100µl of DMEM/F12 medium supplemented with 2% FBS, 1 mmol/L sodium pyruvate, 1% penicillin/streptomycin and 0.1% amphotericin B were seeded and incubated for 5 to 8 days at 37°C. Images were acquired using confocal microscopy (model FV1000; Olympus, Tokyo, Japan). Analysis was performed using ImageJ software and invasion index was calculated measuring the total area over which cancer cells had dispersed (including invading and non-invading cells) and the area of non- invading cells (1 – [non-invading area/total area]).

**Generation of organotypic cultures of EC cells and ESC**

EC orthotopic culture system was set up as previously described(4) with some adaptations. Briefly, 2.5x10^6^ mice ESC or CAFs were resuspended in 100µl of stromal cell medium (composed of DMEM/F12 supplemented with 4% FBS, 2 mmol/L sodium pyruvate, 1% penicillin/streptomycin and 0.5% ITS). This suspension was in turn dissolved in a mixture of 50% Collagen type I (4mg/ml; Thermo Fisher Scientific, 354249), 25% Matrigel, 6.25% FBS and stromal cell medium, generating a gel that was deposited in an M24 well and incubated for 1h at 37ºC with humidity saturation and 5% CO2. Next, 1 ml of stromal cell medium was carefully added to the well and incubated for 16h again at 37ºC with humidity saturation and 5% CO2. Then, the medium was aspirated carefully, and a suspension of 5.5 x 10^5^ tumor cells of interest resuspended in DMEM tumor cell medium was added to a final volume of 500µl, incubation was repeated for 16h at 37ºC with humidity saturation and 5% CO2. Finally, the graft where the culture was mounted was prepared. For this, first 1ml of the mixture of 50% Collagen type I (4mg/ml; Thermo Fisher Scientific, 354249), 25% Matrigel, 6.25% FBS and stromal cell medium was prepared for each graft. This mixture was deposited on the top of the graft and then incubated for 1h at 37ºC. After the hour of incubation, the gels were fixed with sterile PFA 4% for 2h at room temperature; washed three times with PBS 1X for 10min; and neutralized by adding tumor cell medium and incubating for 30min at 37ºC. Finally, these were carefully transferred to M6 wells. Once the grafts were prepared, the gels were placed on the grafts and tumor cell medium was added until reaching the base of the graft and 100µl of the mixture of 50% Collagen type I, 25% Matrigel, 6.25% FBS and stromal cell medium was added to each gel (on top of the tumor cell portion) for better preservation of the structure and orientation of the culture components. Finally, it was incubated for 6-7 days at 37ºC with humidity saturation and 5% CO2. After this period, for the analysis of organotypic cultures, the cultures were fixed with formalin for 16h at 4ºC and H&E was performed. Images were acquired using a Leica DMD170 microscopy. Analysis was performed using ImageJ and the invasion index was calculated by measuring the total area over which each EC cell had dispersed (including invading and non-invading cells) and the area of non- invading cells (1 – [non-invading area/total area]).

**Viral production, infection and *in vitro* transfection conditions**

Oligonucleotides to produce plasmid-based sgRNA were cloned into the lentiviral lentiCRISPRv2 vector using BsmBI restriction sites. sgRNA targets sequence were: ARID1A.1 5’- CACCGATGTTGTTGGTGGAAGACGG; ARID1A.2 5’- CACCGGCTTTCTTCAGCTCCGAGGG; ARID1A.3 5’- CACCGTATGGCCAATATGCCACCTC. Target sequences were functional against both human *ARID1A* and mice *Arid1a* genes.

Production of viral particles was achieved by transfecting HEK-293 packaging cells with linear PEI (40 µM) in combination with lentiviral plasmids and helper plasmids (psPAX2 packaging and pMD2G envelope) at 1:1:1 ratio, respectively. Four hours after transfection, packaging cells were cultured with DMEM supplemented with 10% FBS, 1 mmol/l HEPES (Sigma-Aldrich), 1 mmol/l sodium pyruvate (Sigma-Aldrich), 2 mmol/l L-glutamine (Sigma-Aldrich) and 1% of penicillin/streptomycin (Sigma-Aldrich) for 3-4 days; afterwards the medium containing the viral particles was collected, centrifuged for 10 min at 200 x g and filtered thought a 0.45 µM filter (Millipore, SLHV033RS) and concentrated using Vivaspin concentrators (Sartorius Stedim Biotech GmbH, VS2042). The concentrated medium containing lentiviral particles was added to the medium of the pre-plated cells. Cells were incubated for 24 h. After this period, the medium was replaced with fresh medium, and cells were grown regularly to allow phenotypic expression.

**Genetically modified mouse models**

The in vivo studies complied with Law 5/1995 and Act 214/1997 of the Regional Government (Generalitat de Catalunya) and EU Directive EEC 63/2010 and were approved by the Ethics Committee on Animal Experiments of the University of Lleida and the Ethics Commission in Animal Experimentation of the Generalitat de Catalunya. *Cre-ER^T^* (B6. Cg-Tg(CAG-Cre/Esr1*5Amc/J) and *Pten* ^f/f^ (C;129S4-Ptentm1Hwu/J*)* mice were obtained from the Jackson Laboratory (Bar Harbor, ME, USA). *Arid1a* ^f/f^ mice were a kind gift from Dr. I. Lei. Mice bearing floxed *Arid1a* allele in which exon 9 of *Arid1a* gene is flanked by loxP sites have been described before^21^. *Cre:ERT^+/−^; Pten^f/f^* mice were generated as described previously^22^. *Arid1a^f/f^* mice were backcrossed for five generations with C57BL/6 mice before being crossed with *Cre:ERT^+/−^; Pten^f/f^* transgenic strains to generate epithelial cell-specific deletion of Arid1a. Mice were genotyped by earmarking and DNA was isolated from tail tissue in proteinase K lysis buffer. PCR was carried out with GoTap polymerase (Promega, Madison, WI, USA) using different pairs of primers for each gene. *Cre-ER^+/−^* forward primer 5′-ACGAACCTGGTCGAAATCGTGCG-3′ and reverse primer 5′-CGGTCGATGCAACGAGTGATGAG-3′; *Pten^f/f^* forward primer 5′-CAAGCACTCTGCGAACTGAG-3′ and reverse primer 5′-AAGTTTTTGAAGGCAAGATGC; *Arid1a^f/f^* forward primer 5′- GGCTCTGCCATAAAGCGATCC-3′ and reverse primer 5′-CTCACAAATCTAACCGAGGCCAC-3′. No randomization was applied in the allocation of animals to experimental groups, and no blinding was performed during the experiment or outcome assessment. Group assignment was predetermined based on genotype or treatment conditions, and all animals were handled and processed consistently across groups.

**Tamoxifen administration**

As previously described^22^, tamoxifen (Sigma-Aldrich, T5648) was dissolved in 100% ethanol at 100 mg/ml. Tamoxifen solution was emulsified in corn oil (Sigma-Aldrich C8267) at 10 mg/ml by vortexing. To induce PTEN and ARID1A deletion, adult mice (8 weeks old) were given a single intraperitoneal injection of 0.5 mg of tamoxifen emulsion (30-35 μg per mg body weight).

**Subcutaneous tumor and retro-orbital metastasis xenograft model**

Immunodeficient- SCID­ (Sho-Prkdc^scid^HR^hr^ mouse model) 12-week-old female mice (Charles River) were maintained in specific pathogen-free (SPF) conditions and manipulated in accordance with institutional guidelines of the Committee on Animal Experiments of the University of Lleida and the Ethics Commission in Animal Experimentation of the Generalitat de Catalunya. To establish subcutaneous tumors, 1.5 x 10^4^ MFE 296 EC cells (unlabeled with EGFP-Luciferase reporter gene) were resuspended in 100 µ of 1X PBS and Matrigel (1:1) and injected subcutaneously. Tumors were allowed to grow for 14 days. After this time, and once the subcutaneous tumors were established, 5 x 10^5^ EC MFE-296 cells labeled with the EGFP-Luciferase reporter gene were injected retro-orbitally into the ocular sinus of the same females. Successful retro orbital injection was confirmed on day 0, by systemic bioluminescence imaging; females with evidence of successful injection continued the experiment. Afterwards, metastatic lesions were let to grow for 2 to 3 weeks post retro-orbital injection. Tumor lesions (EGFP-Luciferase positive) were monitored via bioluminescence imaging by Photon Imager (Biospace Measures) coupled with live imaging software M3 Vision Viewer. For bioluminescence tumor imaging, luciferin (Caliper Life Science, #119222) was used as the substrate for the luciferase expressing tumor cells and injected intraperitoneally at 150 mg/kg in PBS. No randomization was applied for the allocation of animals to experimental groups, and no blinding was performed during the experiment or outcome assessment. Groups were assigned based on experimental conditions, and the investigator was aware of the group assignments throughout the study. All animals were handled and processed consistently across groups.

**Immunohistochemical study**

After sacrifice, mice uteri were excised, flushed with PBS, fixed in 10% neutral-buffered formalin and embedded in paraffin. Mice uterus blocks were sectioned at a thickness of 3μm, dried for 1 hour at 65ºC before pre-treatment procedure of deparaffinization, rehydration and epitope retrieval in the Pre-Treatment Module, PT-LINK (Agilent Technologies-DAKO) at 95 °C for 20 min in 50 × Tris/EDTA buffer, pH 9. Before staining the sections, endogenous peroxidase was blocked. The antibodies used were anti-ARID1A (1:500 dilution, Abcam, ab182561), Ki-67 (, Abcam, ab16667, 1:100), Anti-CXCL16 (Sigma-Aldrich, HPA066315,1:100) Anti-CXCR6 (Abcam, ab8023, 1:100), Anti-ADAM10 (Merck, #AB19026,1:100), Anti- αSMA (sc-53015, Santa Cruz Antibodies, 1:100), Anti-FAP. After incubation, the reaction was visualized with the EnVisionTM FLEX+ rabbit (Linker) Detection Kit (Agilent Technologies-DAKO) for ARID1A and other antibodies, secondary antibody polyclonal goat anti rabbit IgG/Biotin (1:200 dilution, Jackson Immunoresearch, 111-065-144) plus Streptavidin/HRP (1:400 dilution, Agilent Technologies-DAKO, P0397)). For Ki67, diaminobenzidine chromogen was used as a substrate. Sections were counterstained with hematoxylin. Appropriate negative controls including no primary antibody were also tested.

Immunohistochemical results were evaluated following uniform pre-established criteria. Immunostaining was graded semi-quantitatively considering the percentage and intensity of the staining. A histological score was obtained from each sample with values ranging from 0 (no immunoreaction) to 300 (maximum immunoreactivity). The score was obtained by applying the following formula: Histoscore=1 × (% light staining) +2 × (% moderate staining) +3 × (% strong staining). To support the scoring of immunohistochemistry, a digital slide scanner [Nuclear Quant Module, Pannoramic 250 FLASH II 2.0 (3D HISTEC) was used and the percentage of positive cells was determined.

**Transwell assay**

1x10^4^ cells in serum-free medium were plated in the upper chamber of a Matrigel coated transwell insert transwell (8µm pore, Falcon, 353097). CM was used as a chemoattractant, alternatively 10^6^cells of interest were previously planted at the well base during 16h. After 48 h, cells were fixed with paraformaldehyde 4% and stained with Hoechst 33342 (5µg/ml; Sigma-Aldrich, B2261). Finally, cells were pictured with an epifluorescence microscope (Leica), before and after a cotton swap. Results were analyzed to obtain the percentage of invasive cells using the software Image J.

**Wound healing assay**

To assess cell migration by wound healing assays, 6x10^4^ cells of interest were seeded per well in a 24-well plate and triplicates of each condition were performed. When the cells had reached 70% confluence, a wound was made with the end of a 200µl tip, and a wash was performed with 1X PBS to remove any remaining cells floating in the medium and fresh medium or the medium of interest to be analyzed was added. For each well, three photographs at different heights were taken at time 0, which were replicated after 48h of incubation at 37°C with humidity saturation and 5% CO2. Finally, the analysis of the % of closed wound was calculated using Image J software.

**Contractibility assay**

To assess contractibility, 250,000 stromal cells of interest per gel were resuspended in 100µl of a mixture of 2mg/ml collagen and Matrigel in a 1:1 ratio. These gels were placed in wells of M24 plates and incubated for 1h at 37°C with humidity saturation and 5% CO2. After this time 500µl of stromal cell medium was added and incubated for 5-6 days. Shrinkage of the collagen gels was monitored by scanning the plates. For analysis of the results, the relative well and gel area was measured using Image J software. Percentage of contractibility was calculated employing the formula 100 × (well area − gel area) / well area.

**Total RNA extraction, reverse transcriptase-PCR and quantitative real-time**

For RT-qPCR, total RNA was extracted from the 3D or 2D cultures using the RNeasy Total RNA kit (Qiagen, Valencia, QIA74104). The cDNA synthesis and RT-qPCR steps were performed simultaneously using the qPCRBIO Probe 1-Step Go kit (PCR Biosystems, K7PB25.44-03). The program used for the process starts by heating the samples at 45°C for 10min, then temperature is raised to 95°C for 2min and finally 40 cycles are repeated at 95°C for 5sec and 60°C for 20sec, using a CFX96TM thermal cycler (BioRad). Relative mRNA expression levels were calculated using the 2^ΔΔCt^ method and are presented as ratios to the housekeeping gene *GAPDH.* Taqman technology from Applied Biosystems was used for real-time RT-qPCR analyses. Probes: mouse *VEGF* (Mm00437304_m1); mouse *IL6* (Mm00446190_m1); mouse *TGF-β1* (Mm00441724_m1); mouse *Cxcl16* (Mm00469712_m1); human *CXCL16* (Hs00222859_m1); mouse *Cxcr6* (Mm02620517_s1); human *CXCR6* (Hs01890898_s1); mouse *Adam10* (Mm00545742_m1); human *ADAM10* (Hs00153853_m1); mouse *Gapdh* (Mm99999915_g1); human *GAPDH* (Hs99999905_m1).

**Immunofluorescence assay**

2D or 3D cultures were fixed with paraformaldehyde 4% for 15 min at room temperature and washed twice with PBS. Depending on primary antibody, cells were permeabilized with 0.2% Triton X-100 in PBS for 10 min or with 100% methanol for 5 min. Next, cultures were incubated overnight at 4ºC with the indicated diluted primary antibodies: anti-α-SMA (1:200, Triton X-100; Santa Cruz, sc-53015); anti-FAP (1:200, Triton X-100; Santa Cruz, sc-100528); anti-COL1A (1:200, Triton X-100; Cell signaling 39952); anti-p-MLY2 (Ser19) (1:200, Triton X-100; Cell signaling, 3671); Rhodamine-conjugated phalloidin (1:500, Triton X-100 Sigma-Aldrich, P1951; anti-CXCL16 (1:200, Triton X-100; Santa Cruz, sc-514363); anti-p- paxillin (Tyr118) (Cell signaling, 2541); anti-GFP (1:1000, Triton X-100; Sigma-Aldrich, A-11122); anti-YAP (Santa Cruz, sc-101199). Then, cells were washed twice with PBS and incubated with PBS containing 5μg/mL of Hoechst 33342 and a 1:500 dilution of secondary anti-mouse Alexa Fluor 546 (Invitrogen, A11005) and Alexa Fluor 488 (Invitrogen, A11029) or anti-rabbit antibodies Alexa Fluor 594 (Invitrogen, R37119) and Alexa Fluor 488 (Invitrogen, A11034) for 4 h at room temperature. Immunofluorescence stains were visualized and analyzed using confocal microscopy (model FV1000; Olympus, Tokyo, Japan) with the 10x, 20x and the oil-immersion 60x magnification objectives. Analysis of obtained images was performed with Fluoview FV100 software (Olympus, Tokyo, Japan) and ImageJ.

**Western blotting**

Western blotting analysis were performed as described previously^23^. Briefly, cells were washed with cold PBS and lysed with lysis buffer (Tris HCl 50mM, NaCl 150mM, 1% Triton X-100, 0,1% de SDS, EDTA 1mM) and sonicated. Protein was quantified by Lowry (BioRad) and equal amounts of protein were subjected to SDS-polyacrylamide gel electrophoresis and transferred to polyvinylidene difluoride membranes. Then membranes were blocked by incubation with TBST (20 mM Tris-HCl (pH 7.4), 150 mM NaCl and 0.1% Tween 20) plus 5% of non-fat milk. Membranes were incubated with the primary antibodies overnight at 4 °C and for 1h at room temperature with secondary horseradish peroxidase (1:10 000 in TBST). Signal was detected with SuperSignal West Femto Trial Kit (Thermo Scientific). Band analysis and densities were determined by using Image Lab 4.0.1 software (Bio-Rad laboratories, Richmond, CA, USA). Full and uncropped Western blot images for all figures are available in the Supplemental material.

**Chemokine array**

For the study of the chemokine secretion profile, the Human Chemokine Array G1 (BioNova, RayBio®; AAH-CHE-G1-4) was used. The first step was to place the chip containing the chemokine probes to be detected under a laminar flow hood for 2h to achieve complete drying. Subsequently, 100µl of blocking buffer was added and incubated for 30min at room temperature. After this time the buffer was aspirated, 100µl of the indicated samples were added to each sub-array and left to incubate for 16h at 4ºC under agitation. Then, a process of consecutive washes with different wash buffers (provided by the kit) was carried out to ensure the elimination of possible non-specific signals. Finally, for detection, 70µl per well of a mixture of biotin-conjugated primary antibodies was added and incubated for 16h at 4ºC with constant agitation. Subsequently, after a sequence of washes, 70µl per well of a fluorophore-labeled streptavidin solution was added (so it was always protected from light) and incubated for 2h at room temperature. Finally, several consecutive washes with different washing buffers were performed and finally it was left to dry under a laminar flow cabinet. Finally, analysis was performed using a laser scanner (Innopsys 'InnoScan®) using the cy3 or "green" channel with an excitation frequency of 532 nm. The fluorescence intensity is proportional to the amount of cytokine in the test sample. Finally, for the heatmap and principal component analysis (PCA), the data were analyzed and represented using ClustVis123 software. For the clustering of the heatmap, the Euclidean distance was used for hierarchical grouping and the data grouping method was based on the average. For the analysis of the relative amount of each cytokine in the different samples, the data were analyzed and represented using GraphPad Prism v8 software.

**CXCL16 Elisa assay**

For the detection and quantification of the CXCL16 chemokine we used two different kits: , the ab187397 (abcam) Elisa kit for human samples and the ab100677 Elisa kit (Abcam) for murine samples. In both cases, 50µl of standard (to calculate the standard line) or sample of interest was first added to each well. Next, 50µl of an antibody solution was added and incubated for 16h at 4ºC at constant agitation. At the end of the incubation, washes were performed with the wash buffer supported by the kit and 100µl of the solution containing the TMB substrates of the HRP to which the antibodies were conjugated was added to set up a colorimetric reaction. After 10min at room temperature under stirring, the reaction was stopped by adding 100µl of the stop solution and the absorbance of the colorimetric signal was analyzed by spectrophotometer (Biotek) at 450nm wavelength. For data analysis, a curve was created with the values of the standards and the data obtained from the samples were extrapolated from this curve, obtaining the concentration of CXCL16 present in each sample.

**TCGA_UCEC Analyses**

RNAseq Data for endometrioid samples from the TCGA_UCEC cohort(5) were downloaded using the TCGAbiolinks R package(6). TPM values were used to stratify samples based on ARID1A expression levels. Specifically, samples were ranked by ARID1A TPM expression, and those in the top 20% (highest expression, ARID1A-preserved) and bottom 20% (lowest expression, ARID1A-loss) were selected for comparative analyses. Next, differential expression analysis was performed on the raw count data using the DESeq2 package(7) with a comparison of ARID1A-loss vs ARID1A-preserved groups. Log2 fold change shrinkage was applied using the lfcShrink function with the apeglm method(8) to improve the accuracy of fold change estimates. Genes were then ranked according to the shrunken log2 fold changes. The resulting ranked gene list from the differential expression analysis was used as input for Gene Set Enrichment Analysis (GSEA) performed with the GSEA Desktop tool from the Broad Institute(9). Custom gene sets were generated from the top 30 genes defining each Cancer-Associated Fibroblast (CAF) subtype, as reported by Gao et al (10).

**Analysis Methylation and Mutational Data**

DNA methylation and mutation data for the *CXCR6* and *CXCL16* genes in endometrial carcinoma were retrieved from The Cancer Genome Atlas Uterine Corpus Endometrial Carcinoma (TCGA-UCEC) cohort(5) using the *TCGAbiolinks* R package(6). Tumors were stratified according to *ARID1A* mutational status, and methylation β-values were compared using the Wilcoxon rank-sum test implemented in R (version 4.4.3).

**Statistical analysis**

All in vitro experiments were carried out with at least three independent biological replicates, each with at least three technical replicates. Results are presented as mean ± standard error of the mean (S.E.M.), with individual data points shown in some figures for human or mouse samples to illustrate data distribution. Statistical significance is indicated as p < 0.05 (*) and p < 0.01 (**). Data normality was assessed using the Kolmogorov-Smirnov test. For parametric data, comparisons between two groups were performed using unpaired two-tailed Student’s t-test, and comparisons among more than two groups were performed using One-way ANOVA followed by Bonferroni post-hoc test. For non-parametric data, comparisons between two groups were performed using Wilcoxon rank-sum test, and comparisons among more than two groups were performed using Kruskal-Wallis test. For categorical outcomes in vivo, Chi-square test was applied. For in vivo studies, no formal statistical power analysis was conducted; sample sizes were determined based on previous experience and ethical considerations. All statistical tests were applied according to the assumptions of the data, and the specific test used for each analysis is indicated in the figure legends.

REFERENCES

1. Megino-Luque C, Sisó P, Mota-Martorell N, Navaridas R, de la Rosa I, Urdanibia I, et al. ARID1A-deficient cells require HDAC6 for progression of endometrial carcinoma. Mol Oncol. 2022;16(11):2235-59.

2. Liangsupree T, Multia E, Riekkola ML. Modern isolation and separation techniques for extracellular vesicles. J Chromatogr A. 2021;1636:461773.

3. Eritja N, Llobet D, Domingo M, Santacana M, Yeramian A, Matias-Guiu X, et al. A novel three-dimensional culture system of polarized epithelial cells to study endometrial carcinogenesis. Am J Pathol. 2010;176(6):2722-31.

4. Ranftl RE, Calvo F. Analysis of Breast Cancer Cell Invasion Using an Organotypic Culture System. Methods Mol Biol. 2017;1612:199-212.

5. Kandoth C, Schultz N, Cherniack AD, Akbani R, Liu Y, Shen H, et al. Integrated genomic characterization of endometrial carcinoma. Nature. 2013;497(7447):67-73.

6. Colaprico A, Silva TC, Olsen C, Garofano L, Cava C, Garolini D, et al. TCGAbiolinks: an R/Bioconductor package for integrative analysis of TCGA data. Nucleic Acids Res. 2016;44(8):e71.

7. Love MI, Huber W, Anders S. Moderated estimation of fold change and dispersion for RNA-seq data with DESeq2. Genome Biol. 2014;15(12):550.

8. Chen GK, Marjoram P, Wall JD. Fast and flexible simulation of DNA sequence data. Genome Res. 2009;19(1):136-42.

9. Subramanian A, Tamayo P, Mootha VK, Mukherjee S, Ebert BL, Gillette MA, et al. Gene set enrichment analysis: a knowledge-based approach for interpreting genome-wide expression profiles. Proc Natl Acad Sci U S A. 2005;102(43):15545-50.

10. Gao Z, Zhang N, An B, Li D, Fang Z, Xu D. Comprehensive analyses of the cancer-associated fibroblast subtypes and their score system for prediction of outcomes and immunosuppressive microenvironment in prostate cancer. Cancer Cell Int. 2024;24(1):127.

**
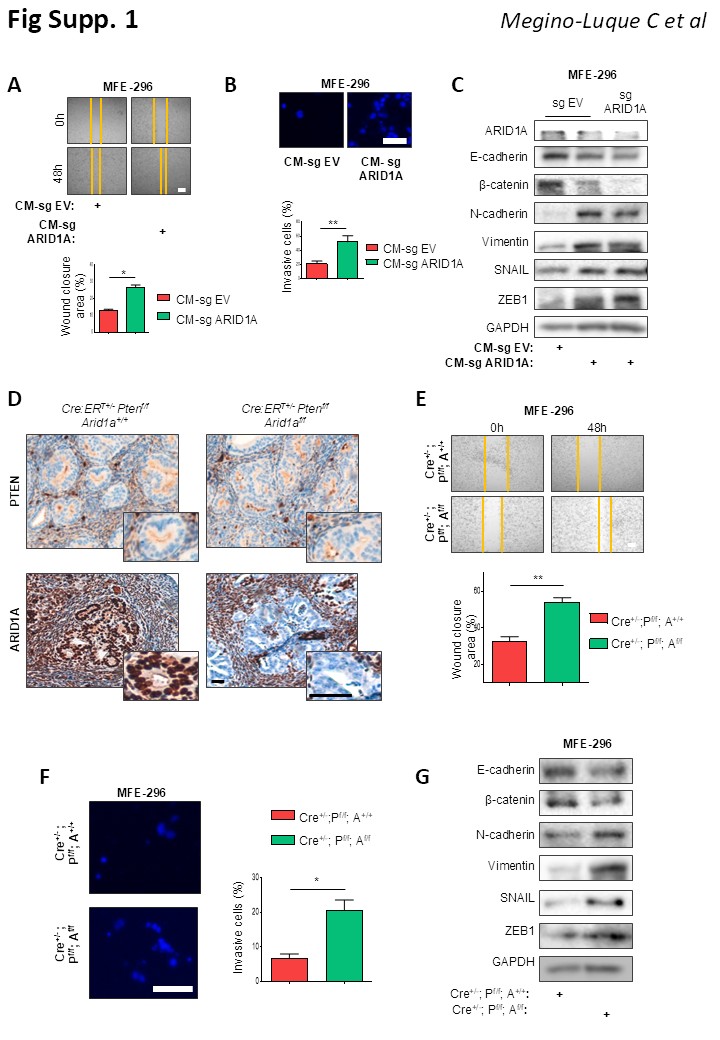

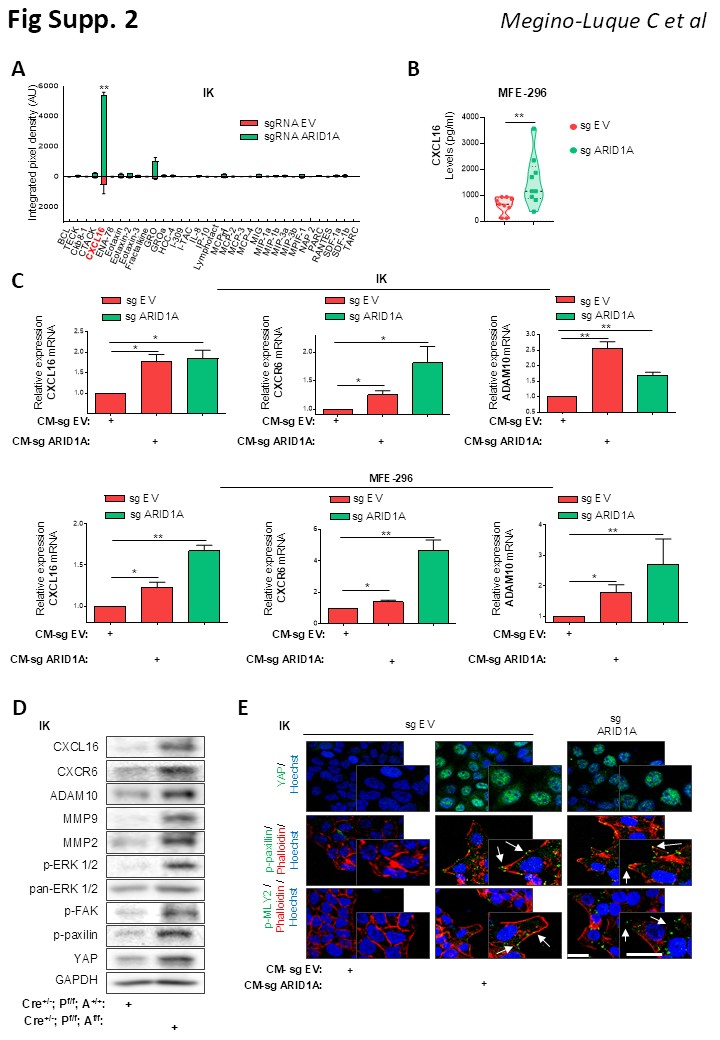

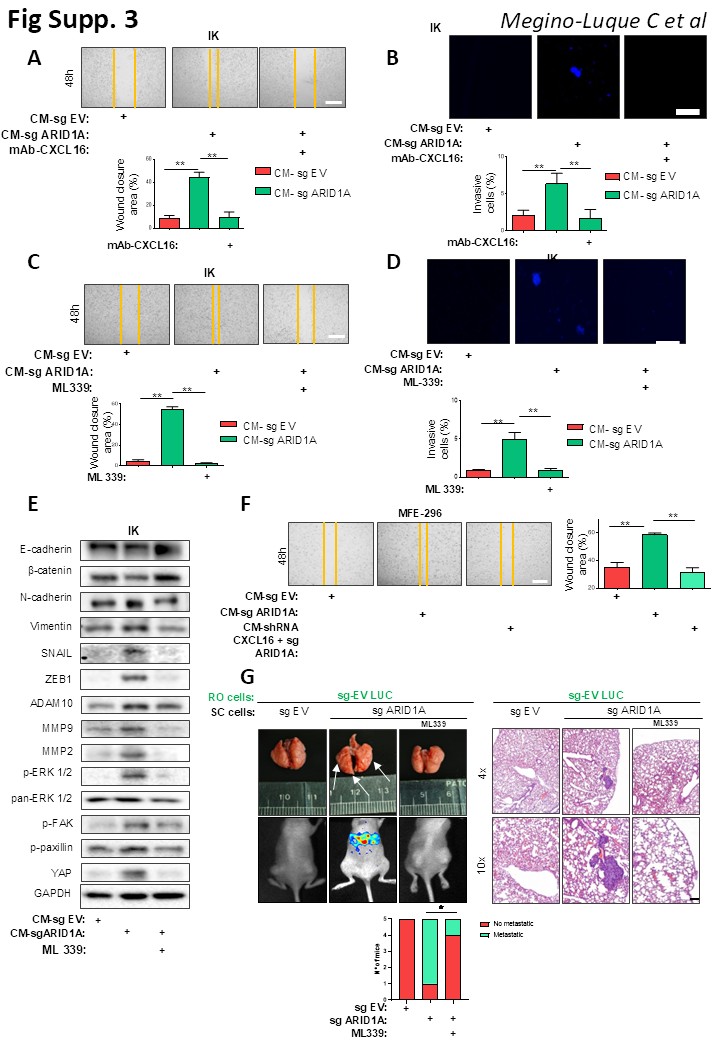

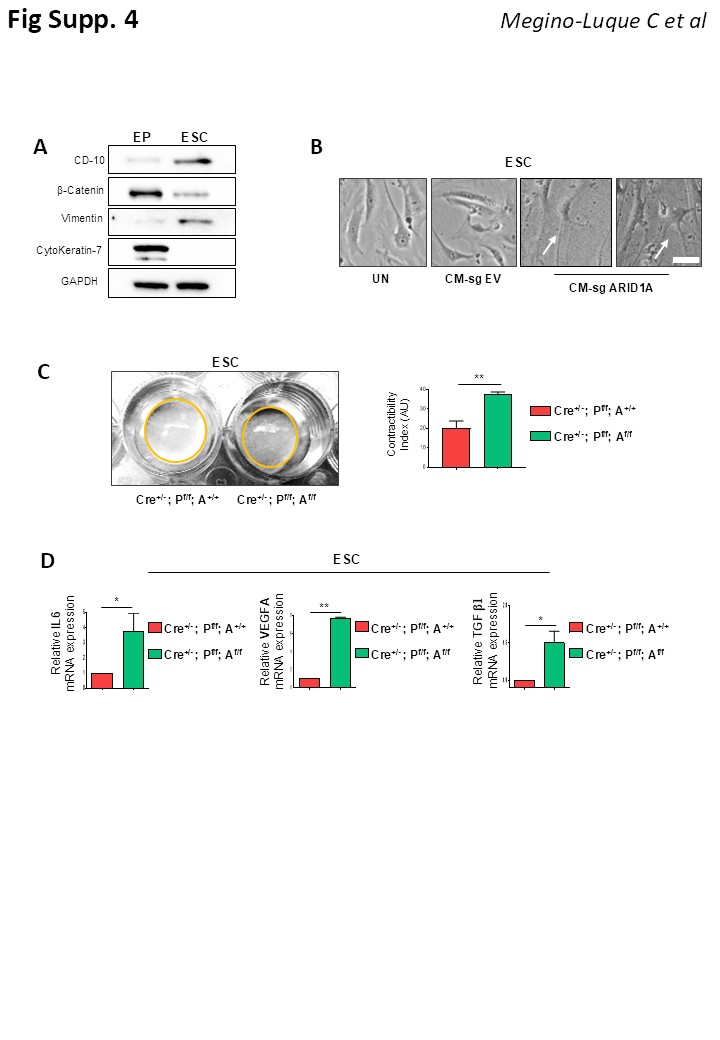

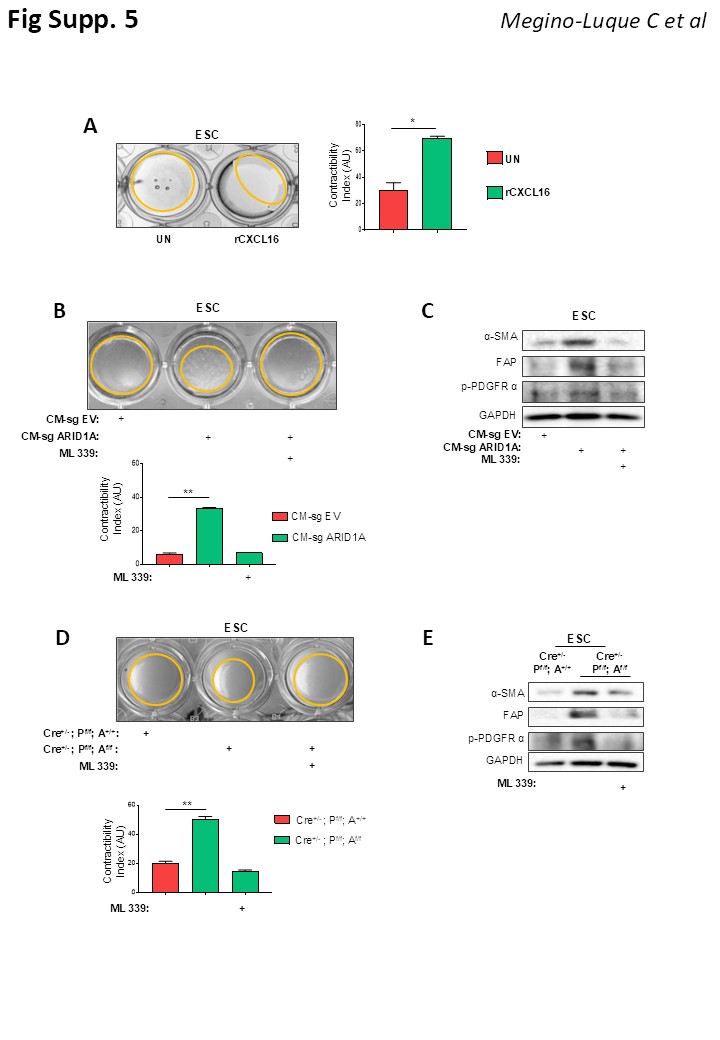
**
