# Supplementary material for: ARID1A deficiency reprograms the tumor secretome, enhancing microenvironmental remodeling and metastatic dissemination in endometrial carcinoma
Source: Cell Death Dis. 2026 Apr 10;17(1):488. doi: 10.1038/s41419-026-08723-z (PMC13186946; doi:10.1038/s41419-026-08723-z)

Figure 1D

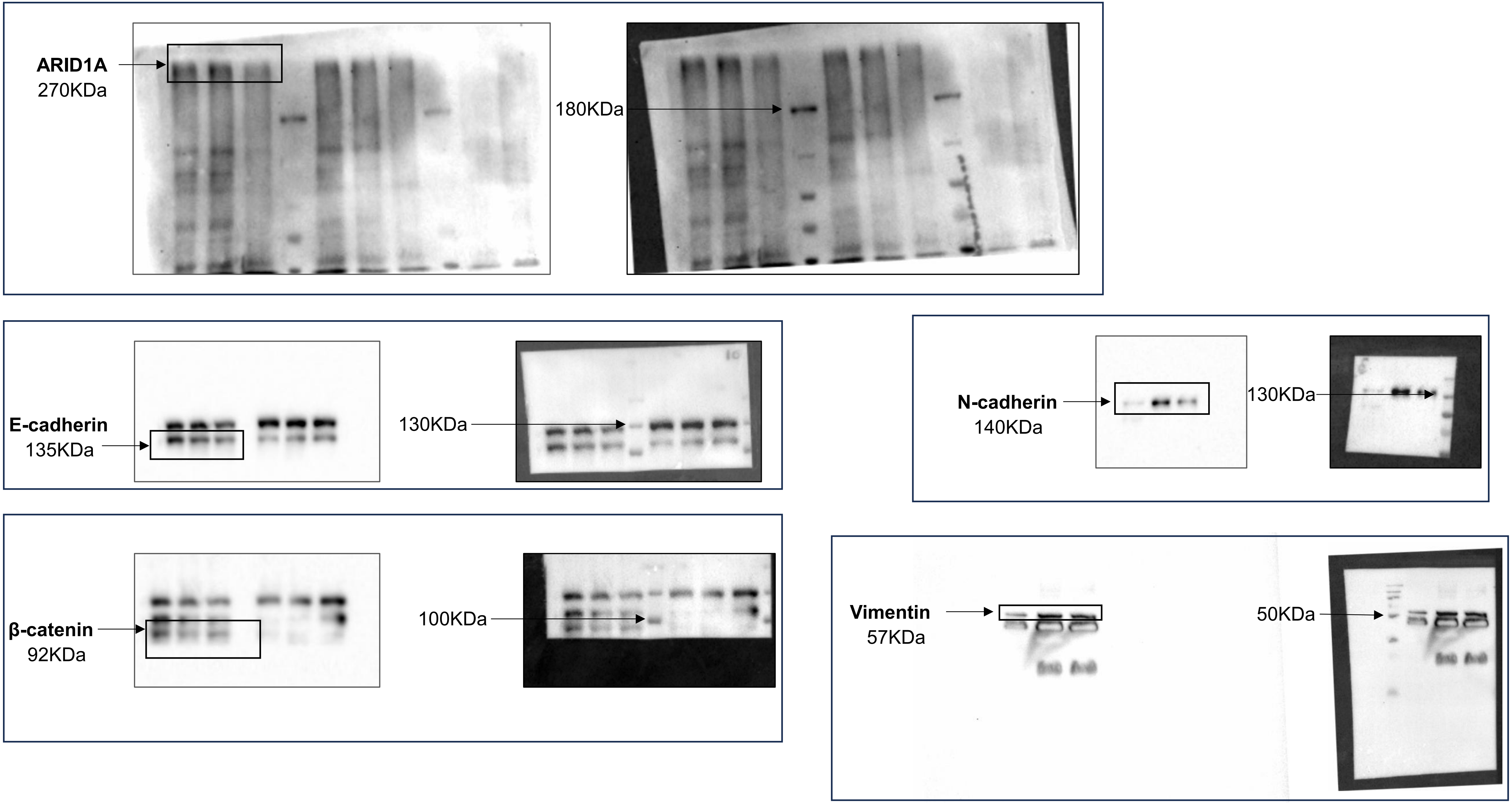
ARID1A
270KDa
180KDa
E-cadherin
135KDa
130KDa
N-cadherin
140KDa
130KDa
β-catenin
92KDa
100KDa
Vimentin
57KDa
50KDa

Figure 1D

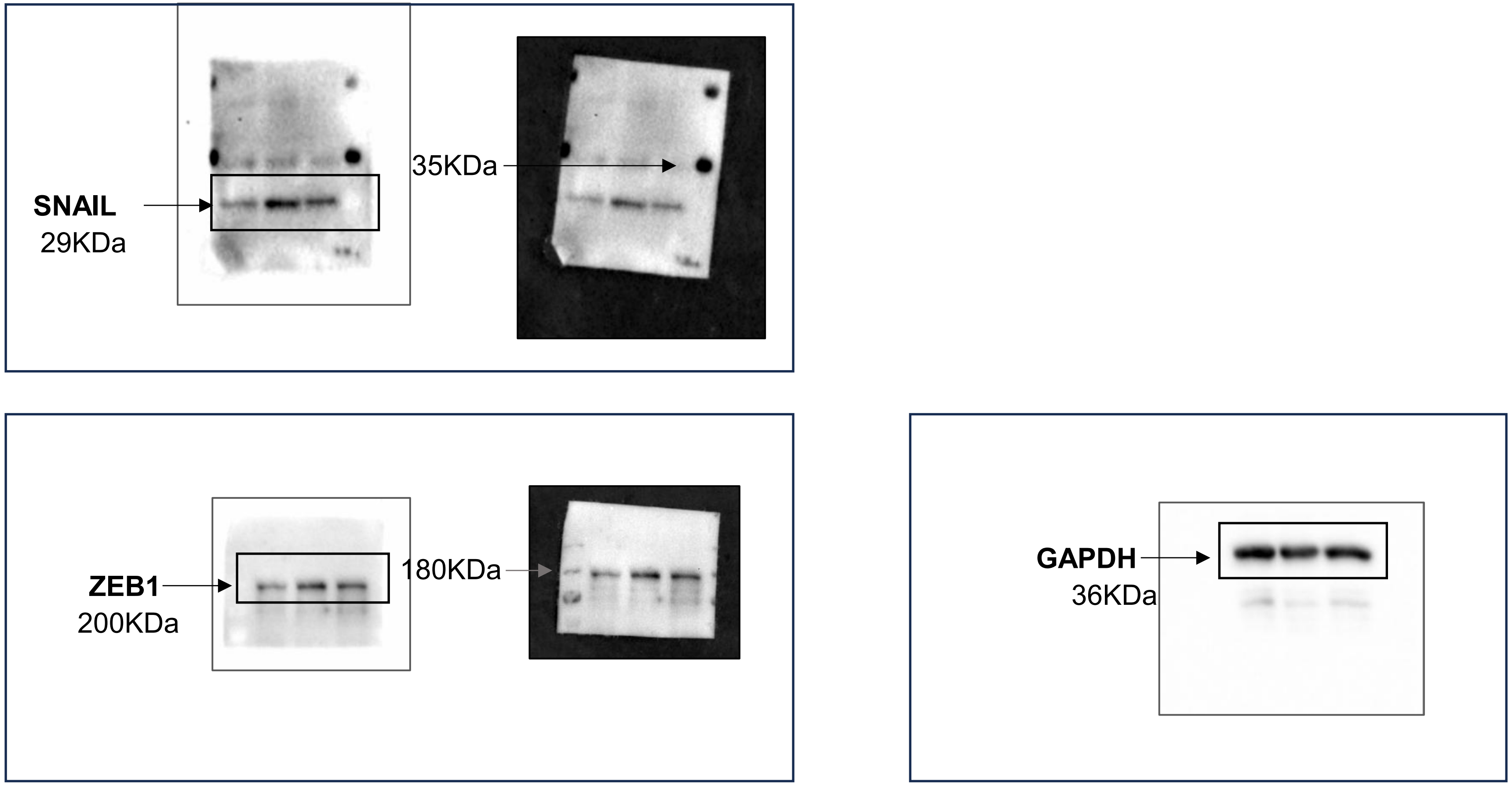

Figure 1H

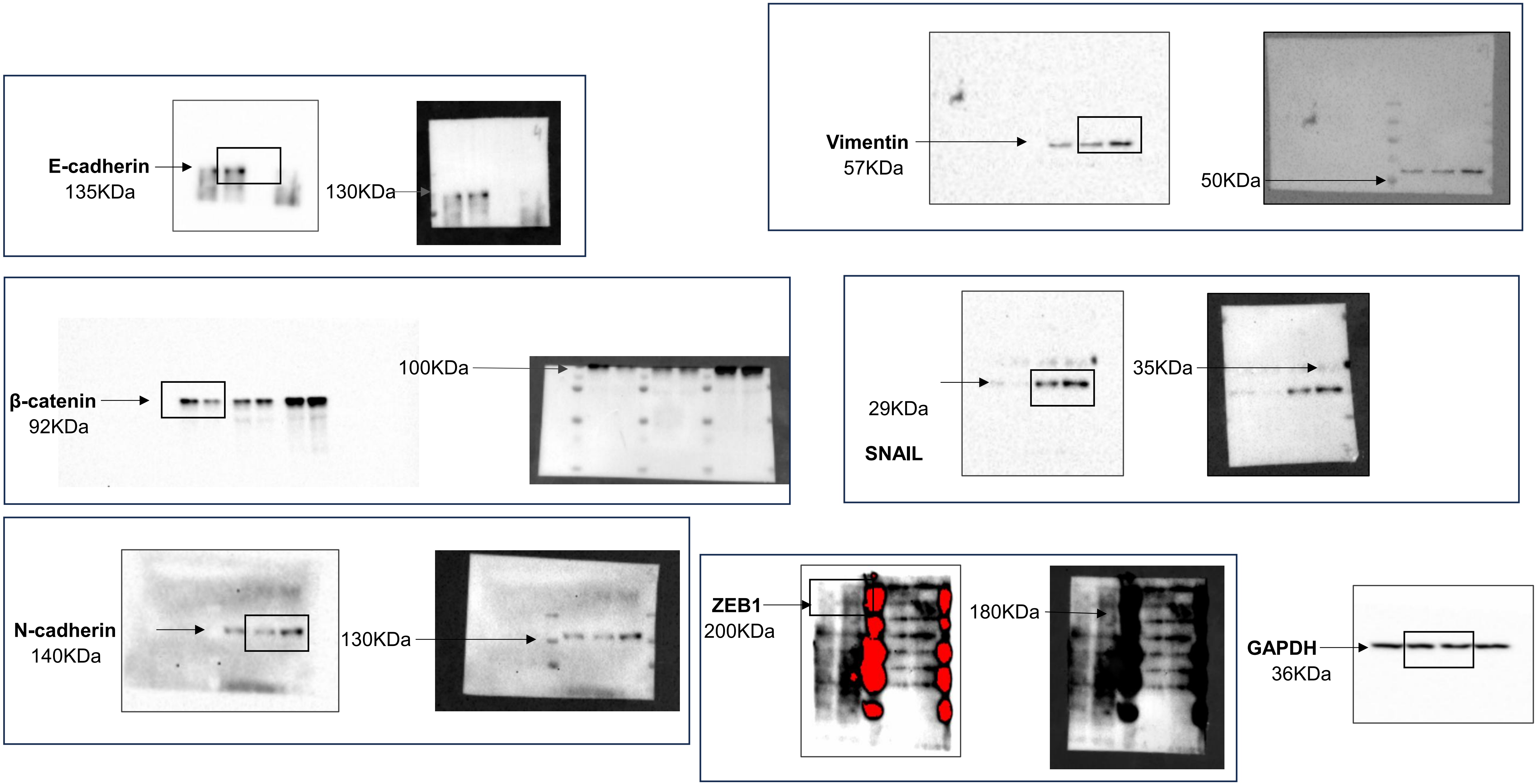
E-cadherin
135KDa
130KDa
Vimentin
57KDa
50KDa
β-catenin
92KDa
100KDa
29KDa
SNAIL
35KDa
N-cadherin
140KDa
130KDa
ZEB1
200KDa
180KDa
GAPDH
36KDa

Figure 2D

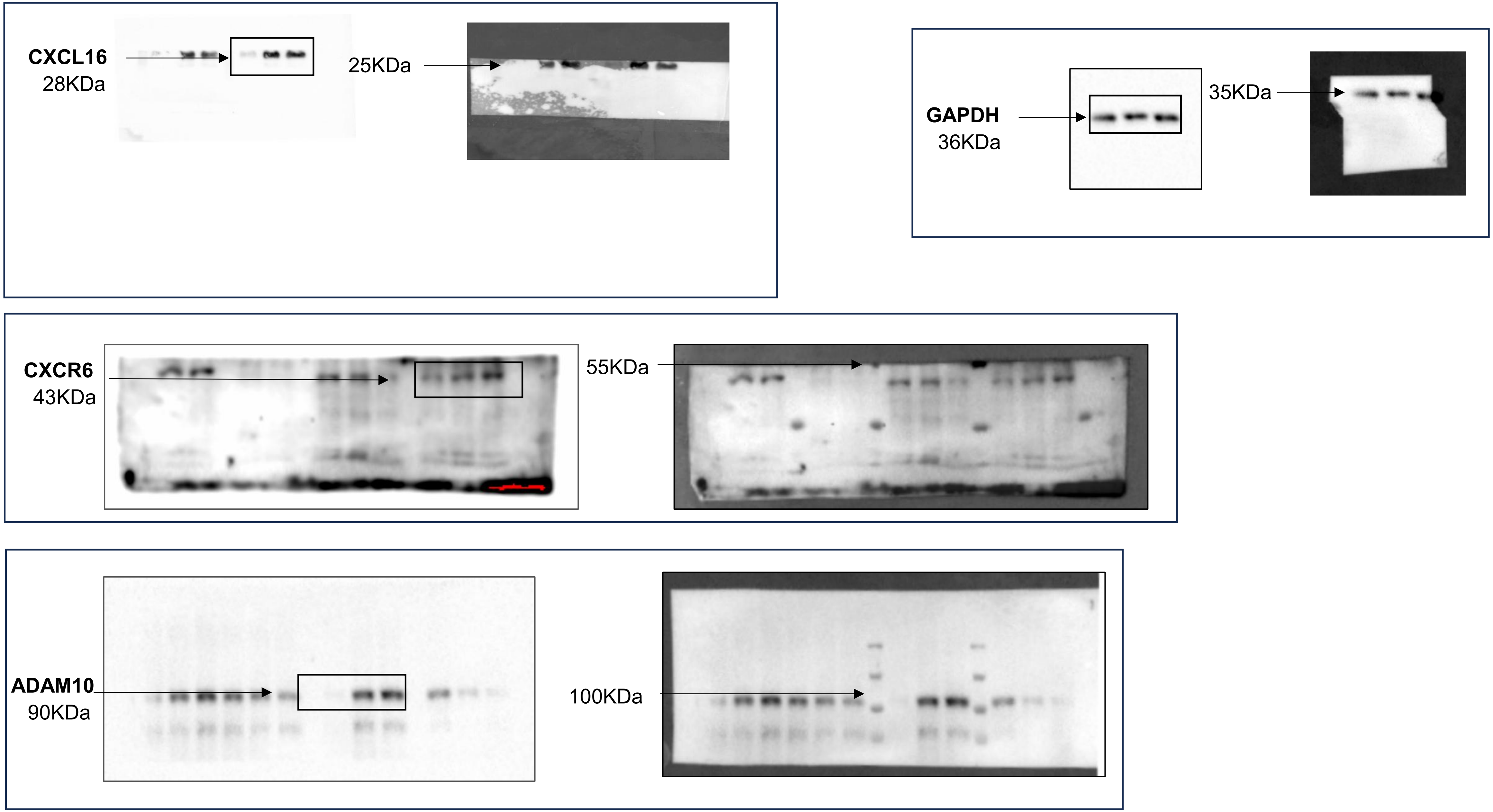
CXCL16
28KDa
25KDa
GAPDH
36KDa
35KDa
CXCR6
43KDa
55KDa
ADAM10
90KDa
100KDa

Figure 2F

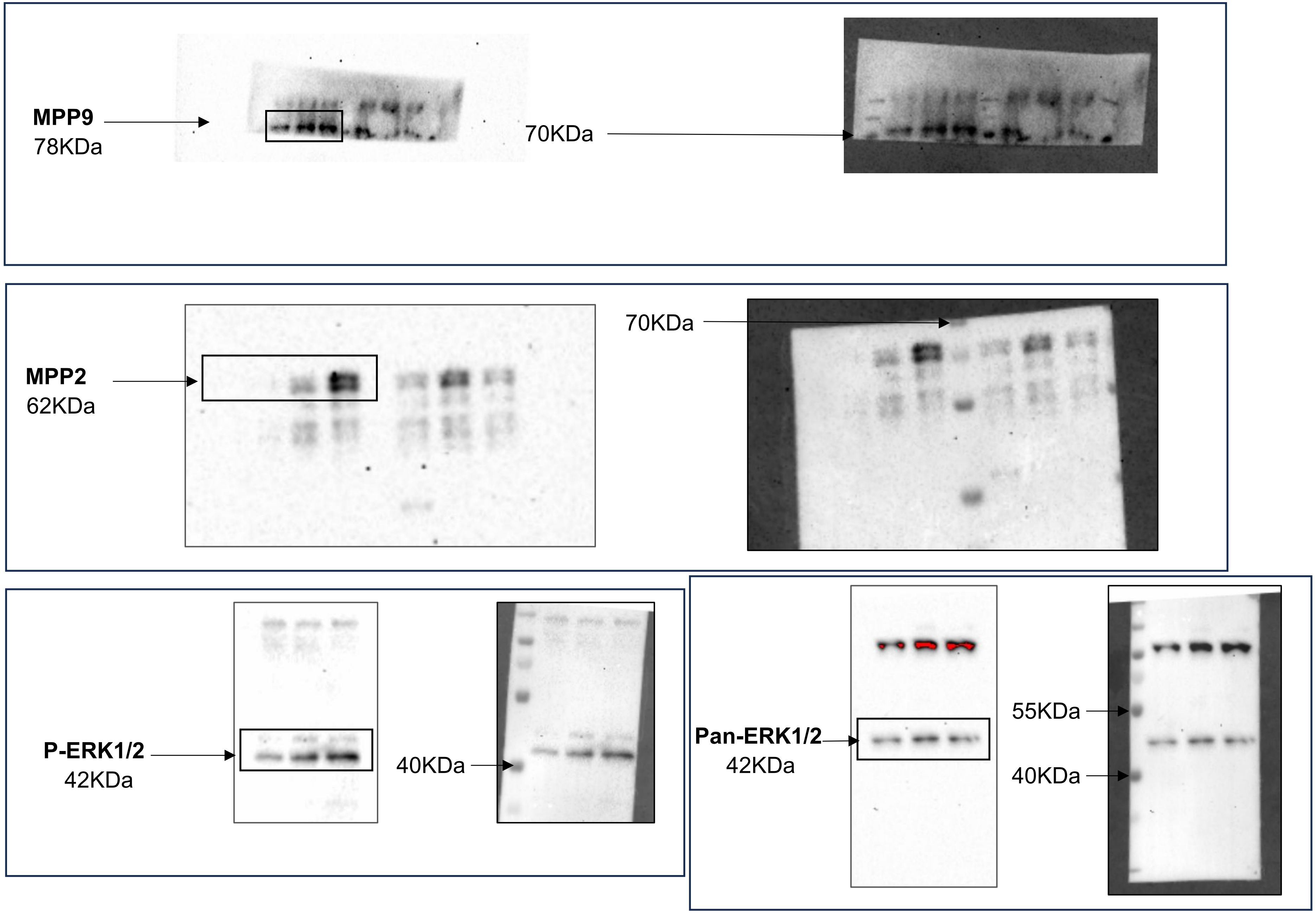
MPP9
78KDa
70KDa
MPP2
62KDa
70KDa
P-ERK1/2
42KDa
40KDa
Pan-ERK1/2
42KDa
55KDa
40KDa

Figure 2F

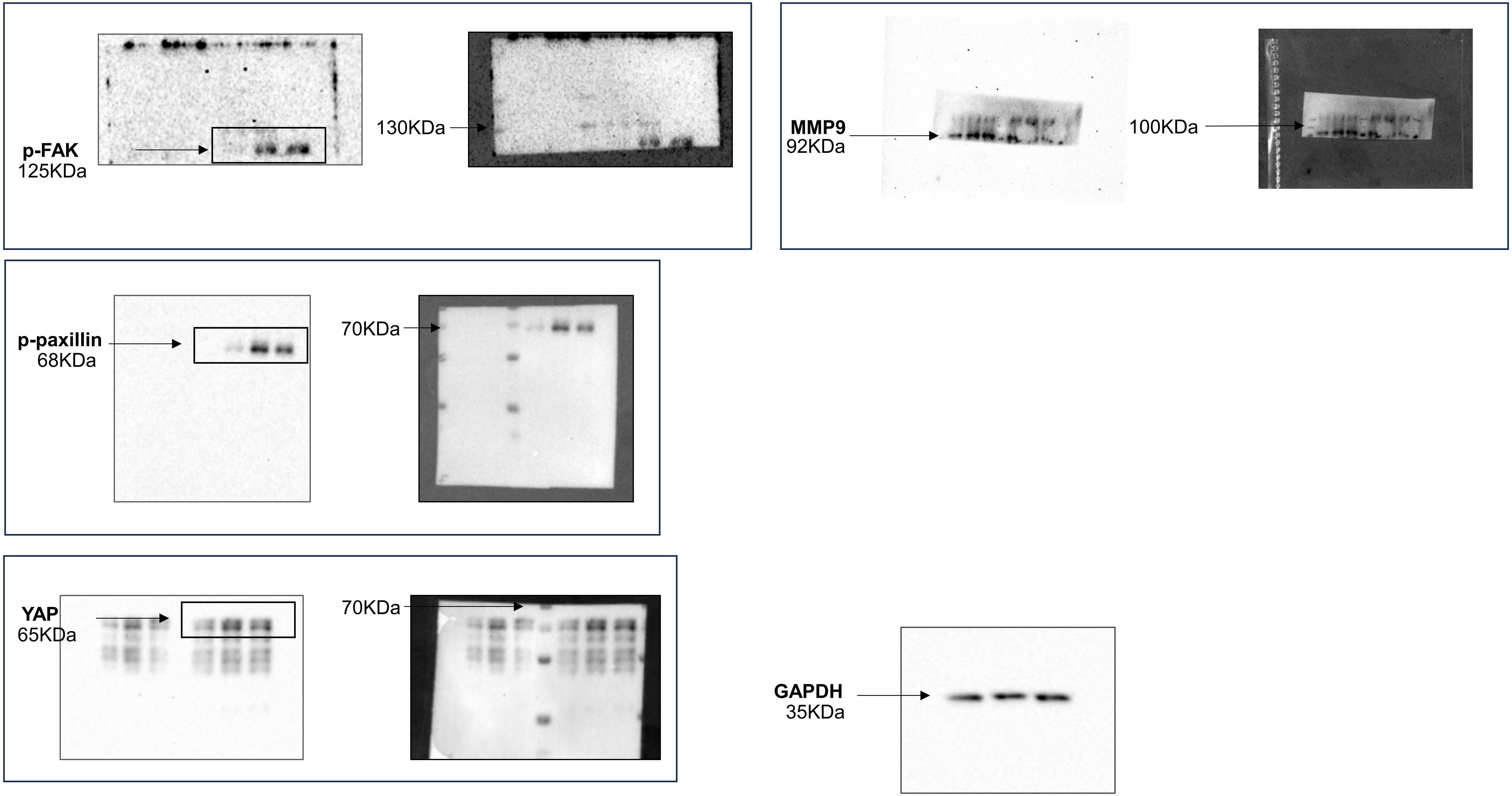
p-FAK
125KDa
130KDa
MMP9
92KDa
100KDa
p-paxillin
68KDa
70KDa
YAP
65KDa
70KDa
GAPDH
35KDa

Figure 3A

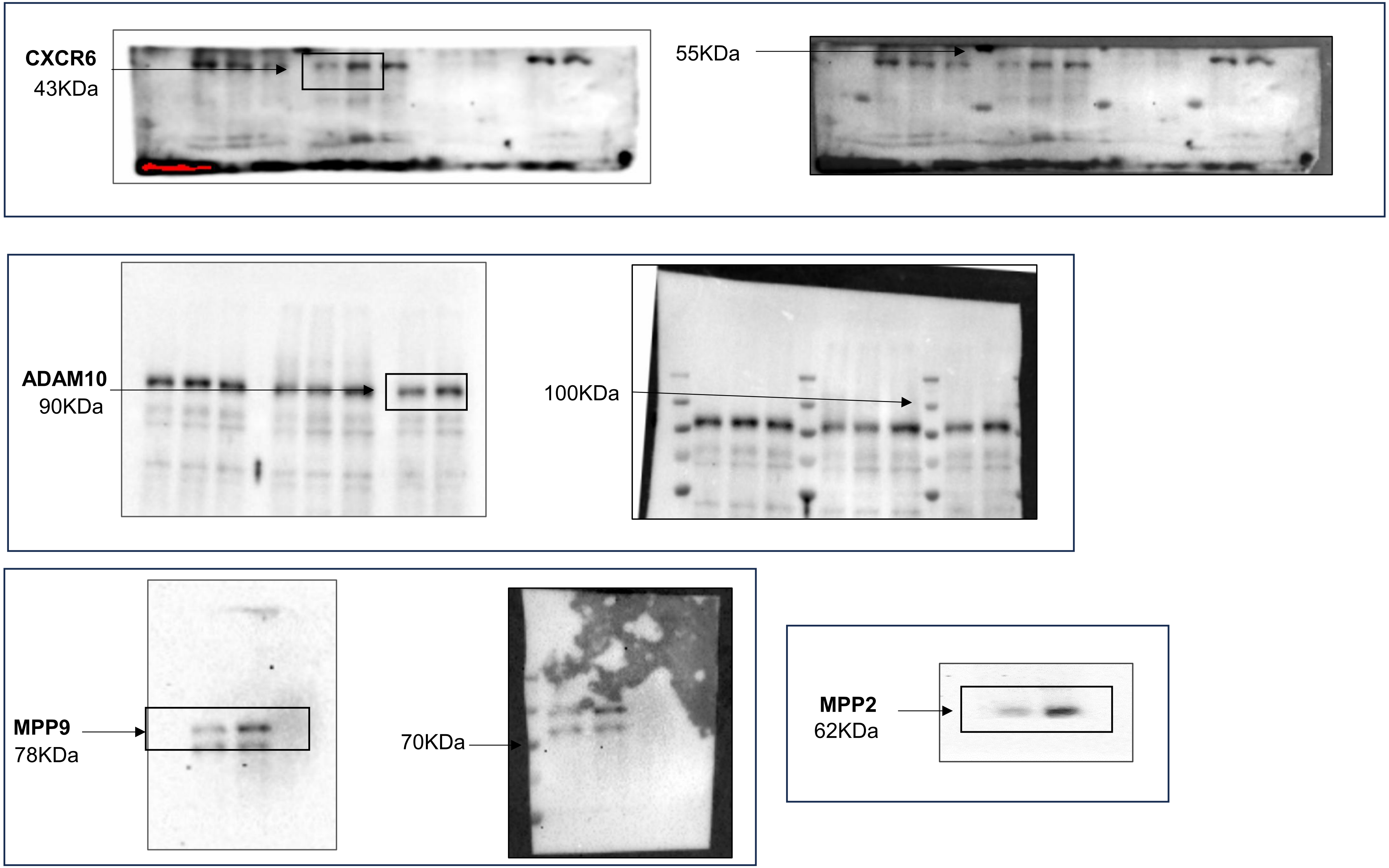
CXCR6
43KDa
55KDa
ADAM10
90KDa
100KDa
MPP9
78KDa
70KDa
MPP2
62KDa

Figure 3A

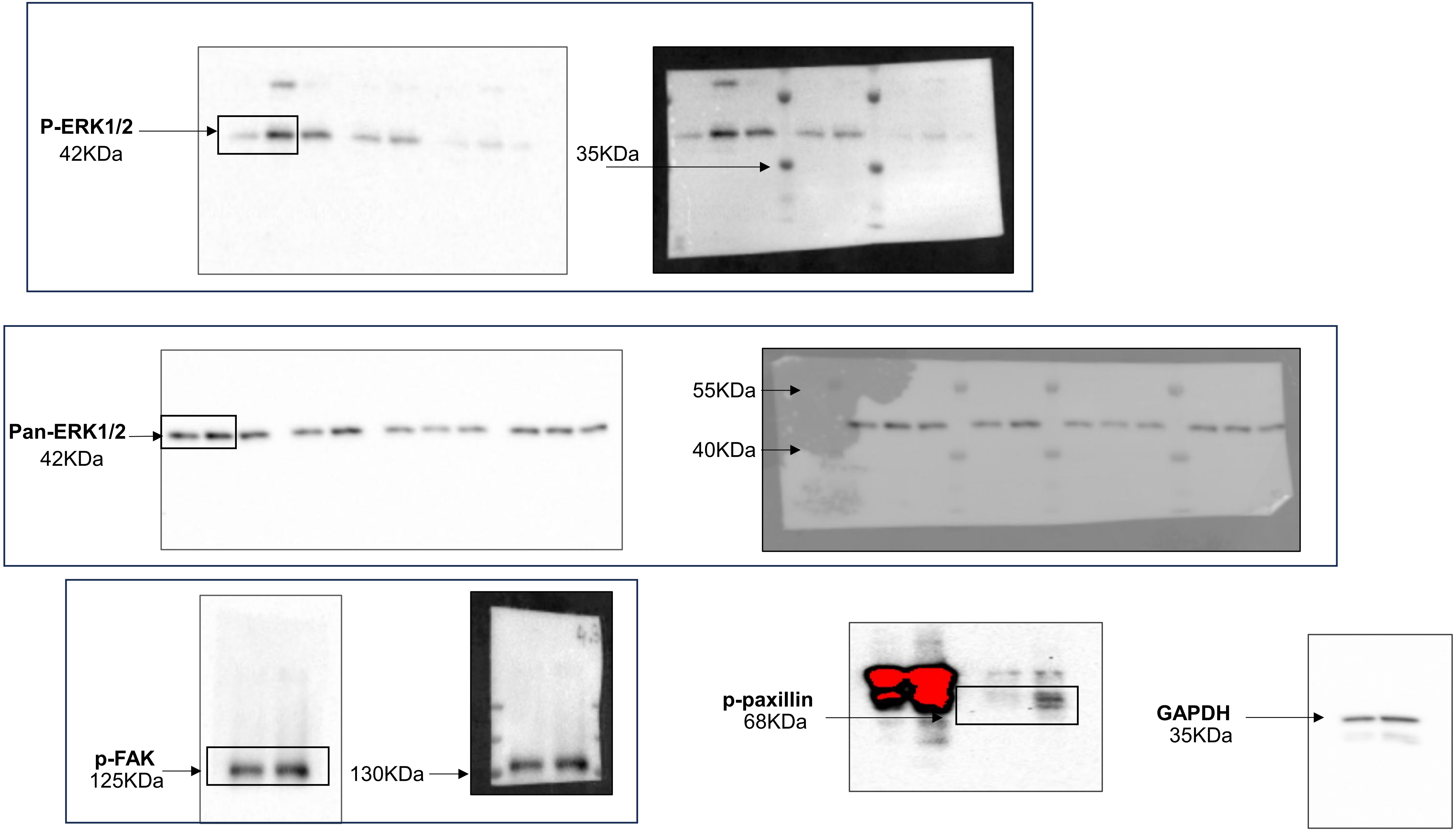
P-ERK1/2
42KDa
35KDa
Pan-ERK1/2
42KDa
55KDa
40KDa
p-FAK
125KDa
130KDa
p-paxillin
68KDa
GAPDH
35KDa

Figure 3D

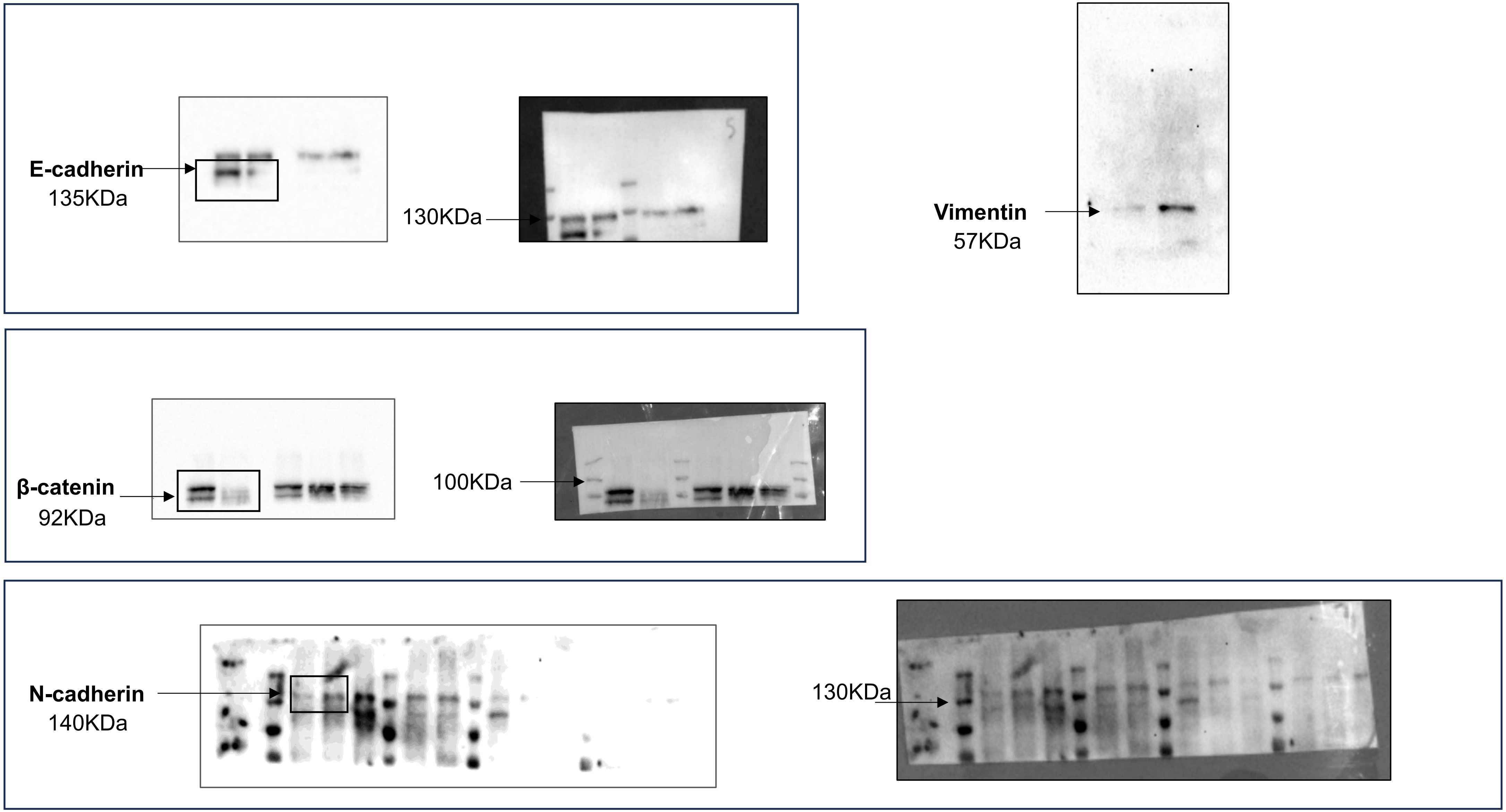
E-cadherin
135KDa
130KDa
Vimentin
57KDa
β-catenin
92KDa
100KDa
N-cadherin
140KDa
130KDa

Figure 3D

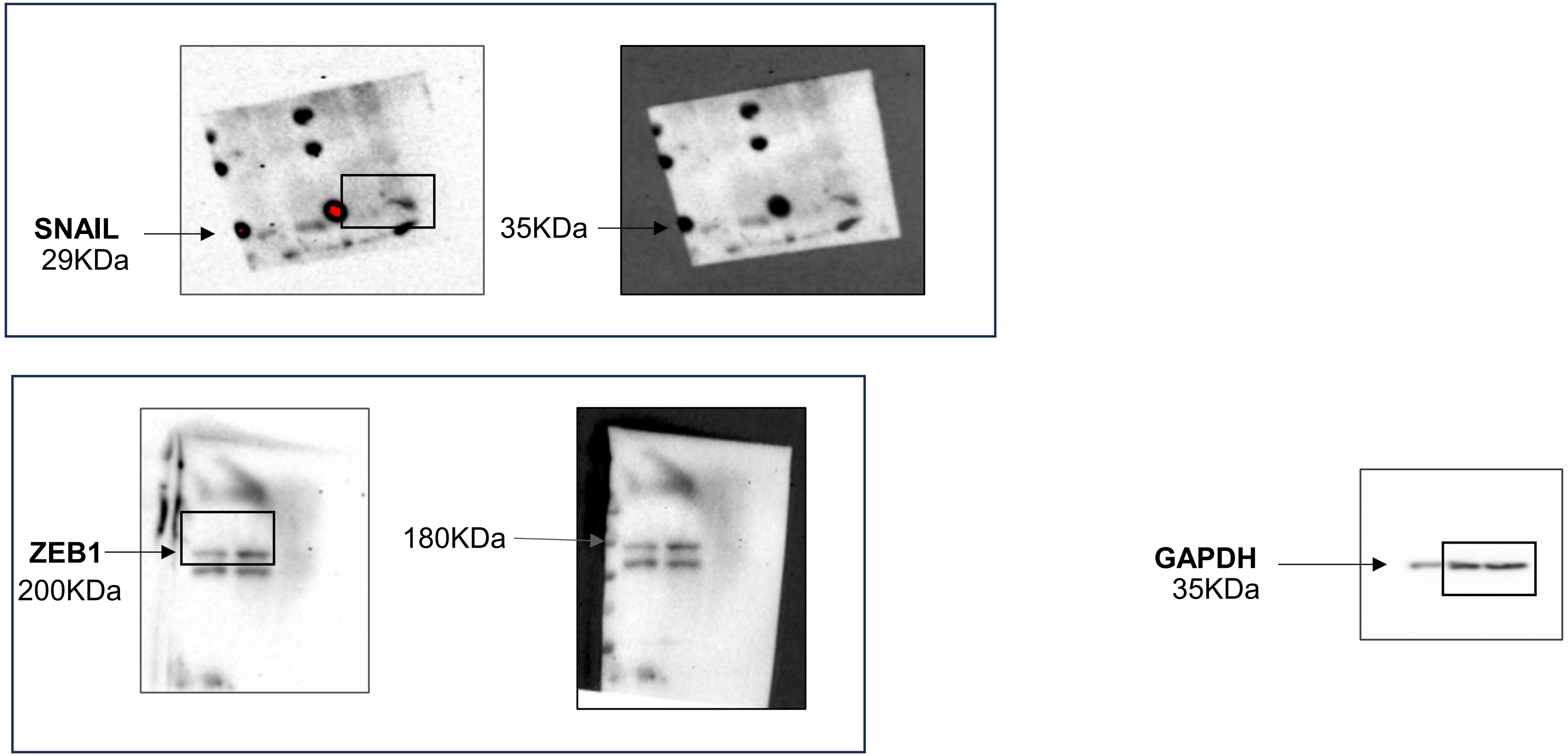

Figure 3E

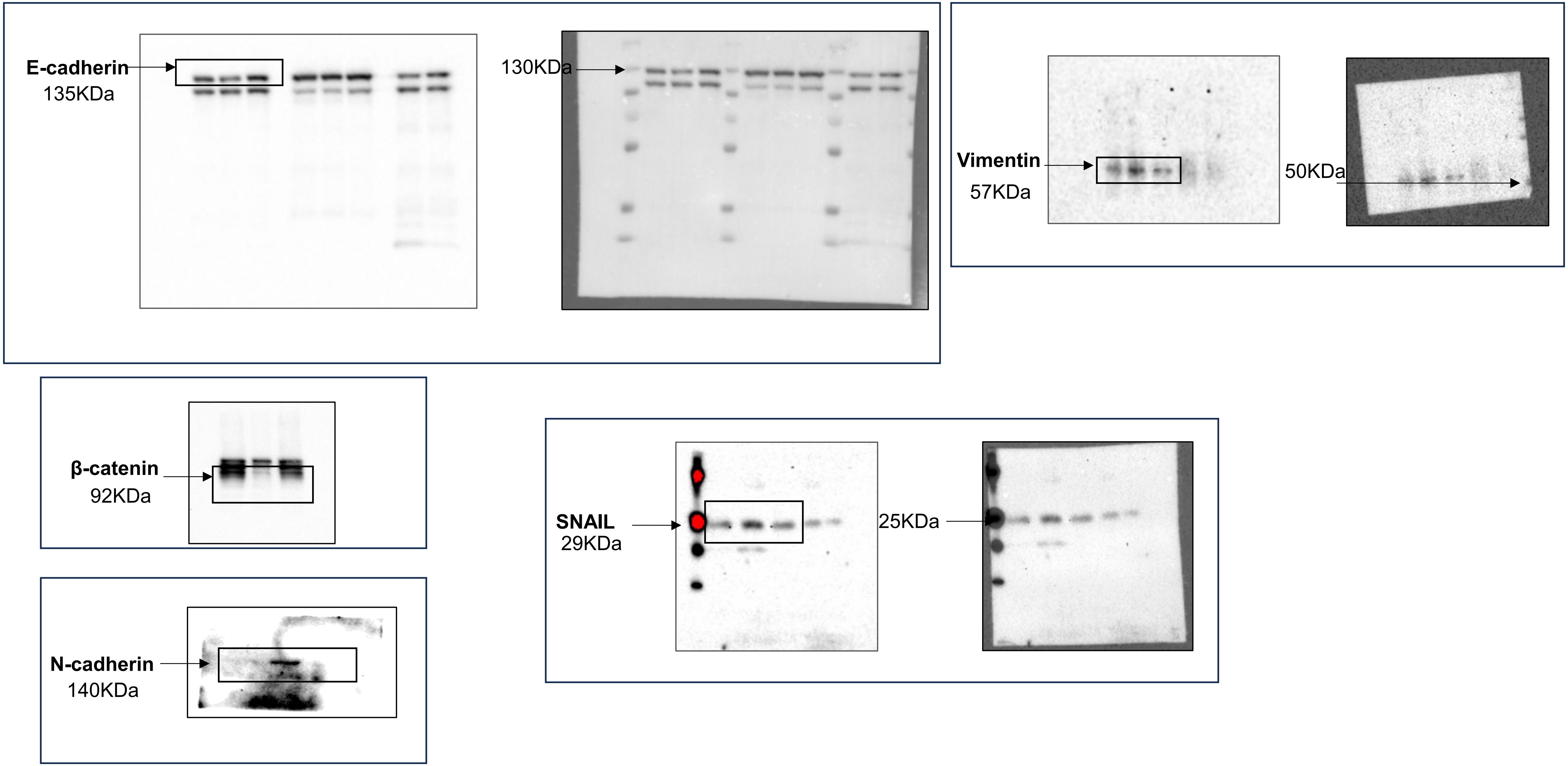
E-cadherin
135KDa
130KDa
Vimentin
57KDa
50KDa
β-catenin
92KDa
N-cadherin
140KDa
SNAIL
29KDa
25KDa

Figure 3E

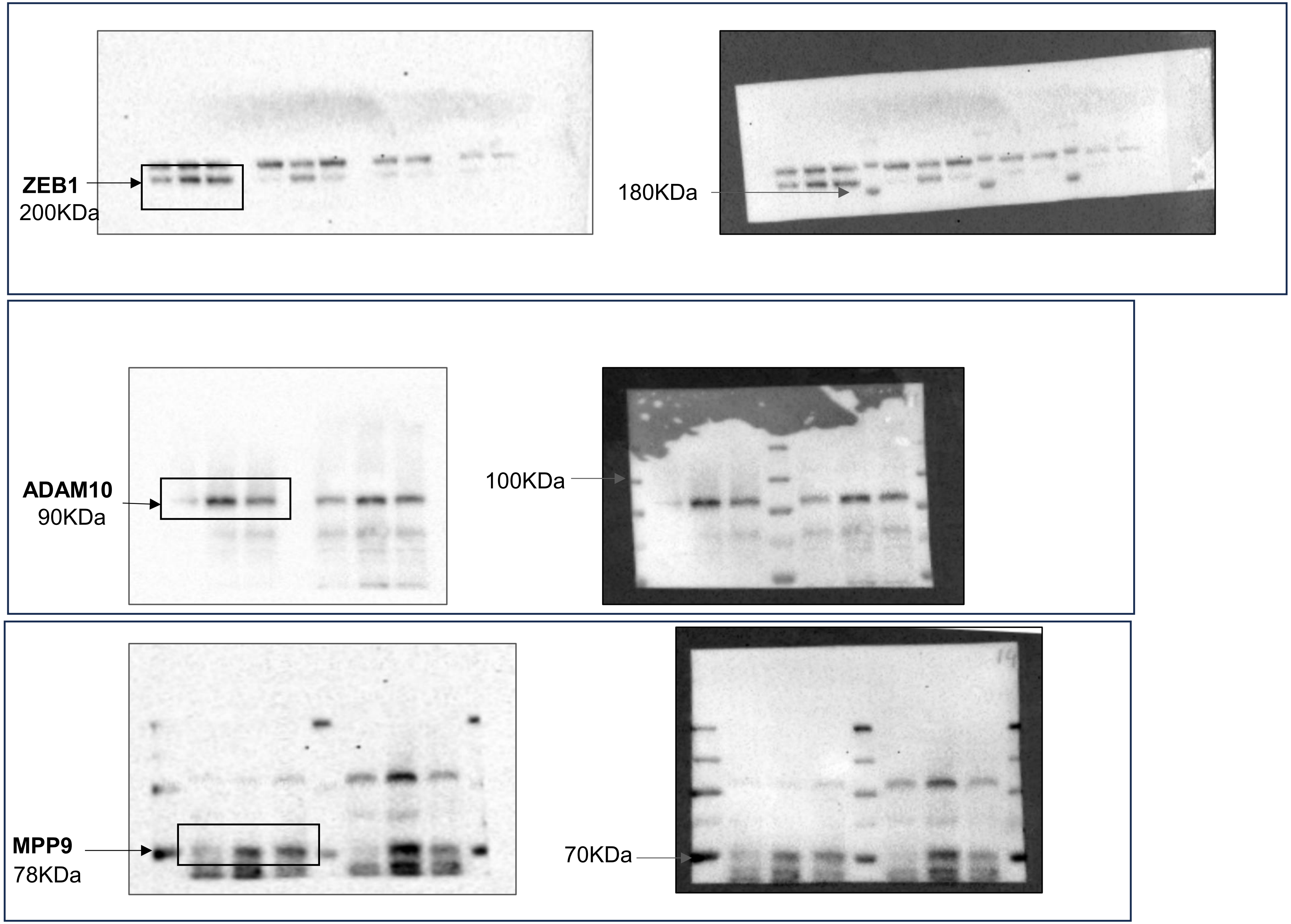

Figure 3E

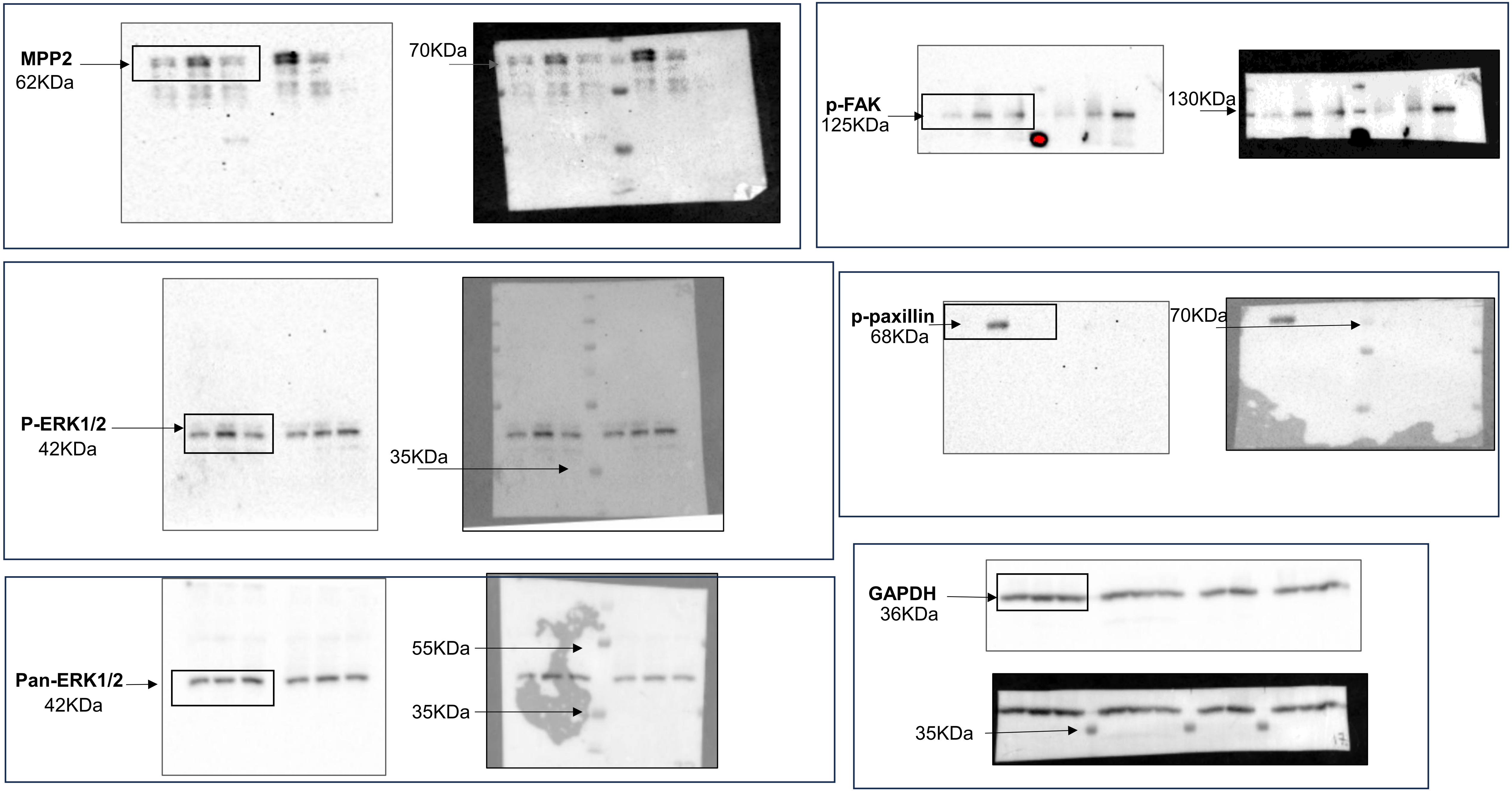
MPP2
62KDa
70KDa
p-FAK
125KDa
130KDa
P-ERK1/2
42KDa
35KDa
p-paxillin
68KDa
70KDa
Pan-ERK1/2
42KDa
55KDa
35KDa
GAPDH
36KDa
35KDa

Figure 3F

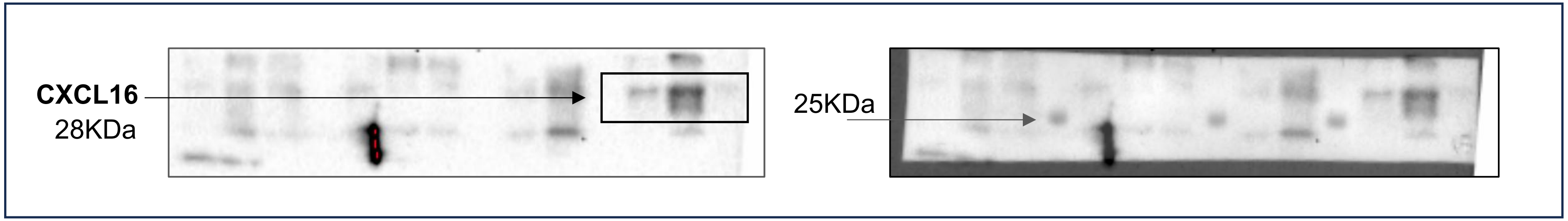

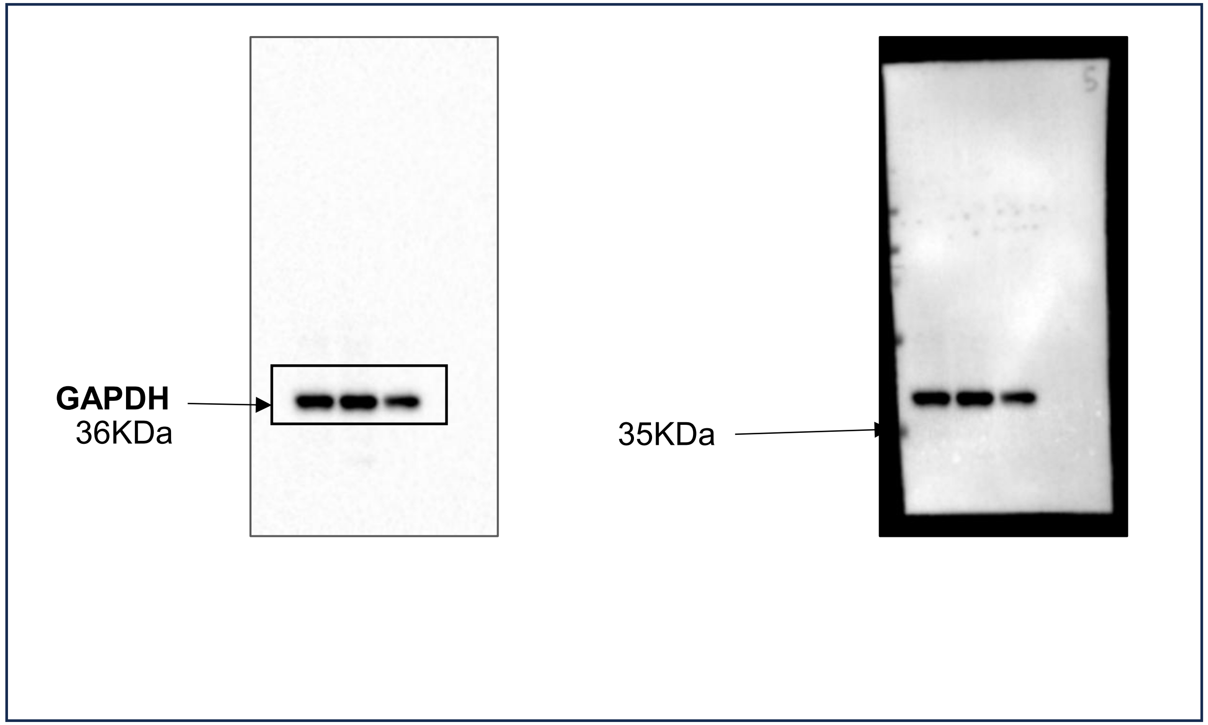

Figure 5D

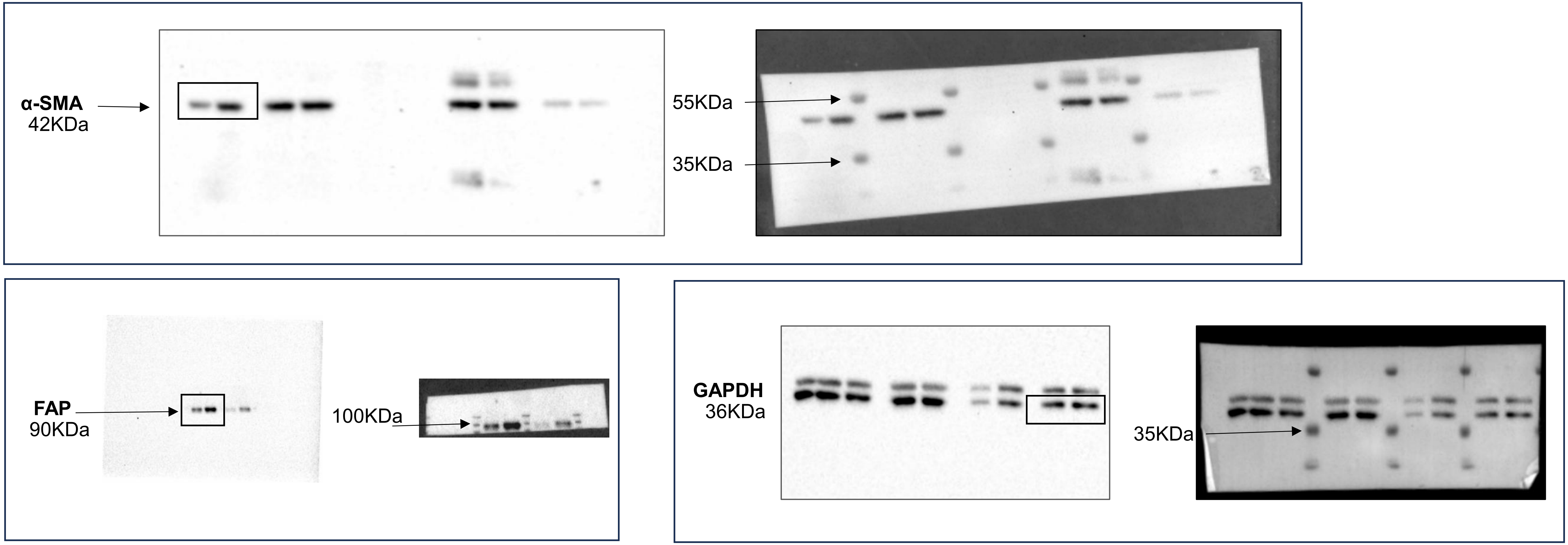

Figure 5D

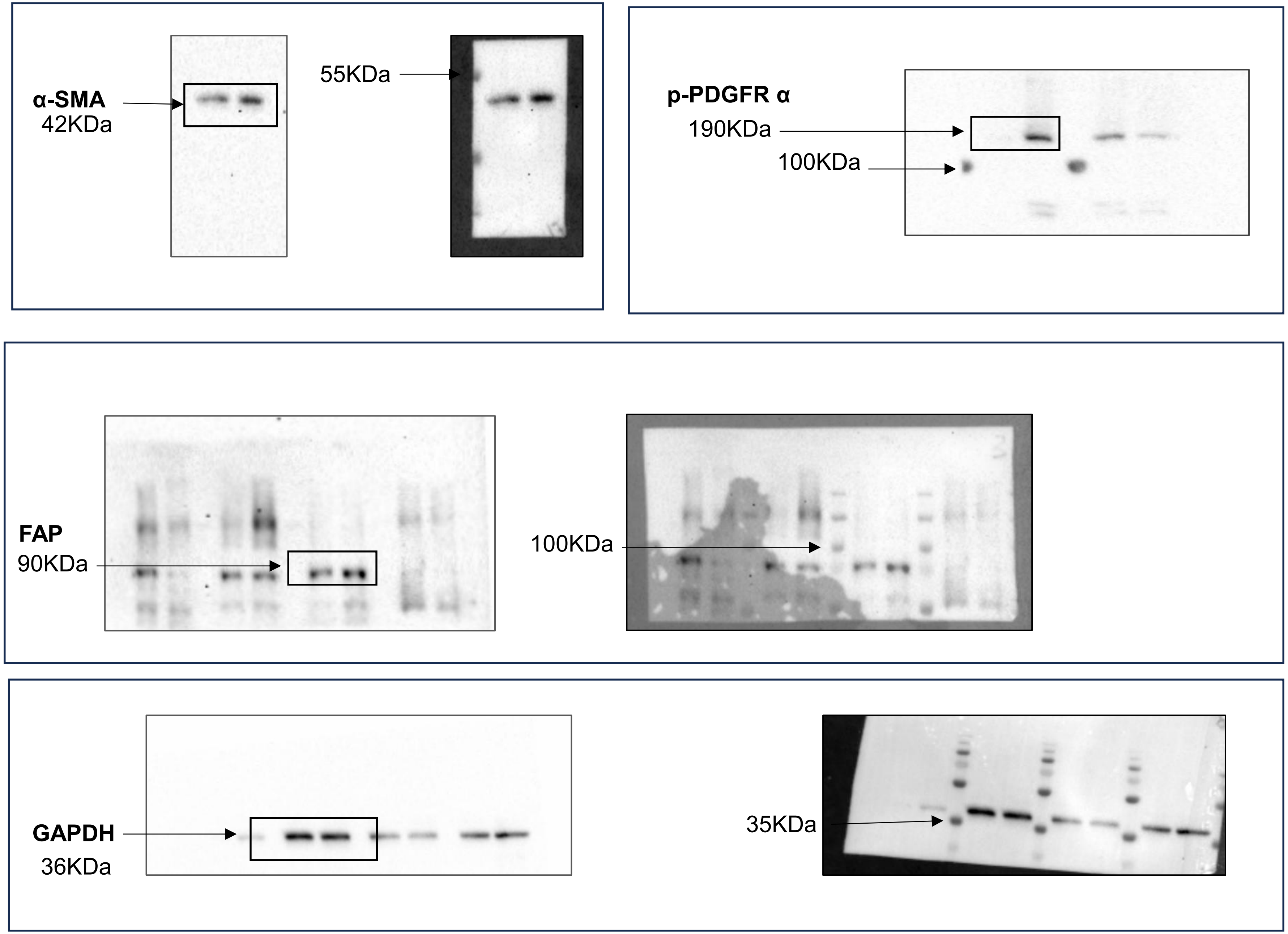

α-SMA
42KDa
55KDa
p-PDGFR α
190KDa
100KDa
FAP
90KDa
100KDa
GAPDH
36KDa
35KDa

Figure 7A

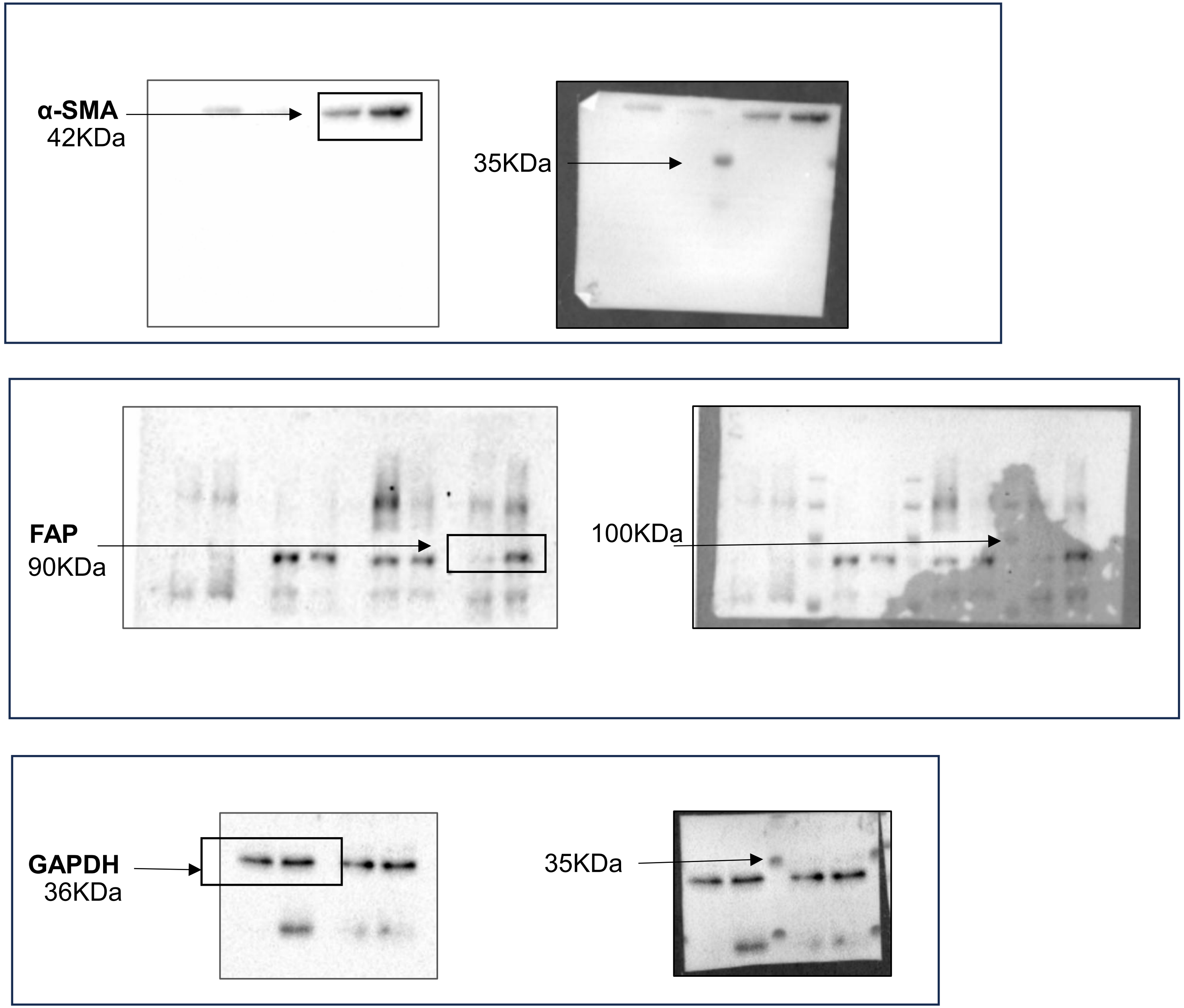

Supp Figure 1C

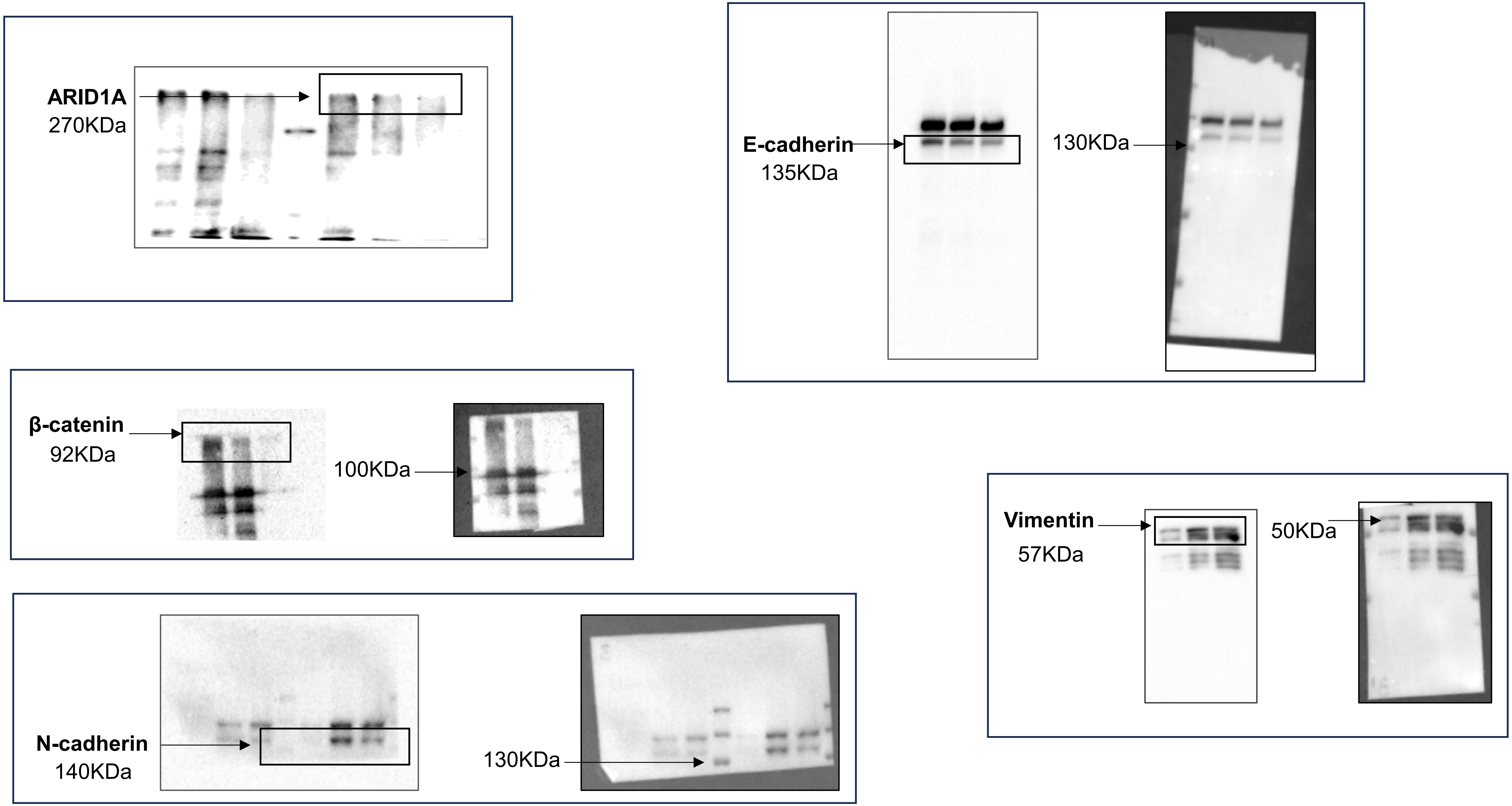

Supp Figure 1C

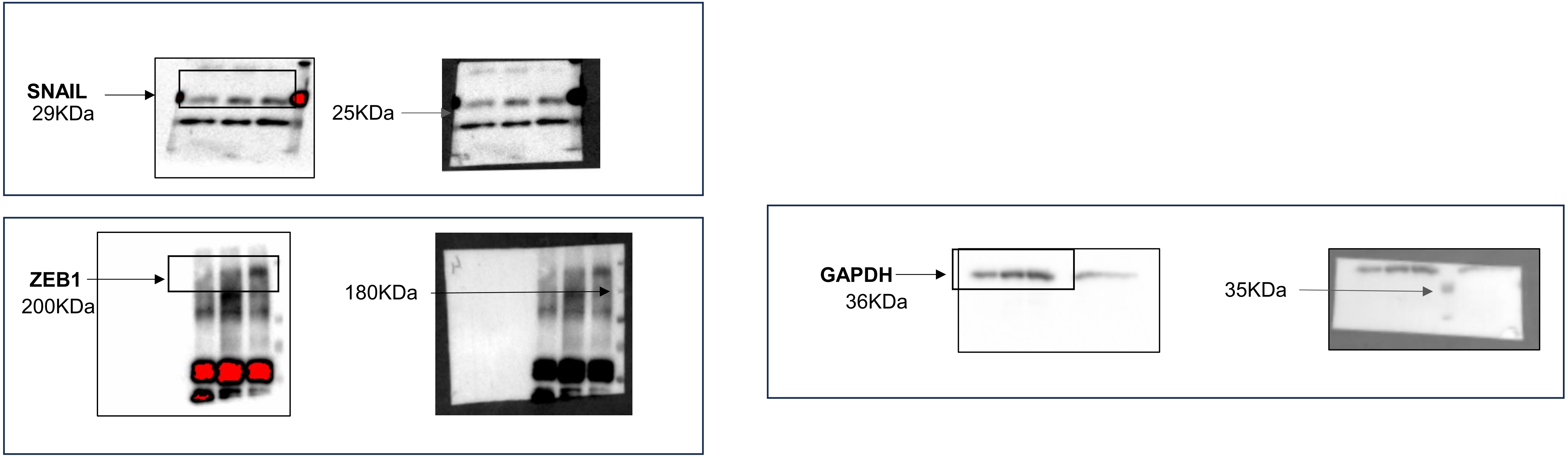

Supp Figure 1G

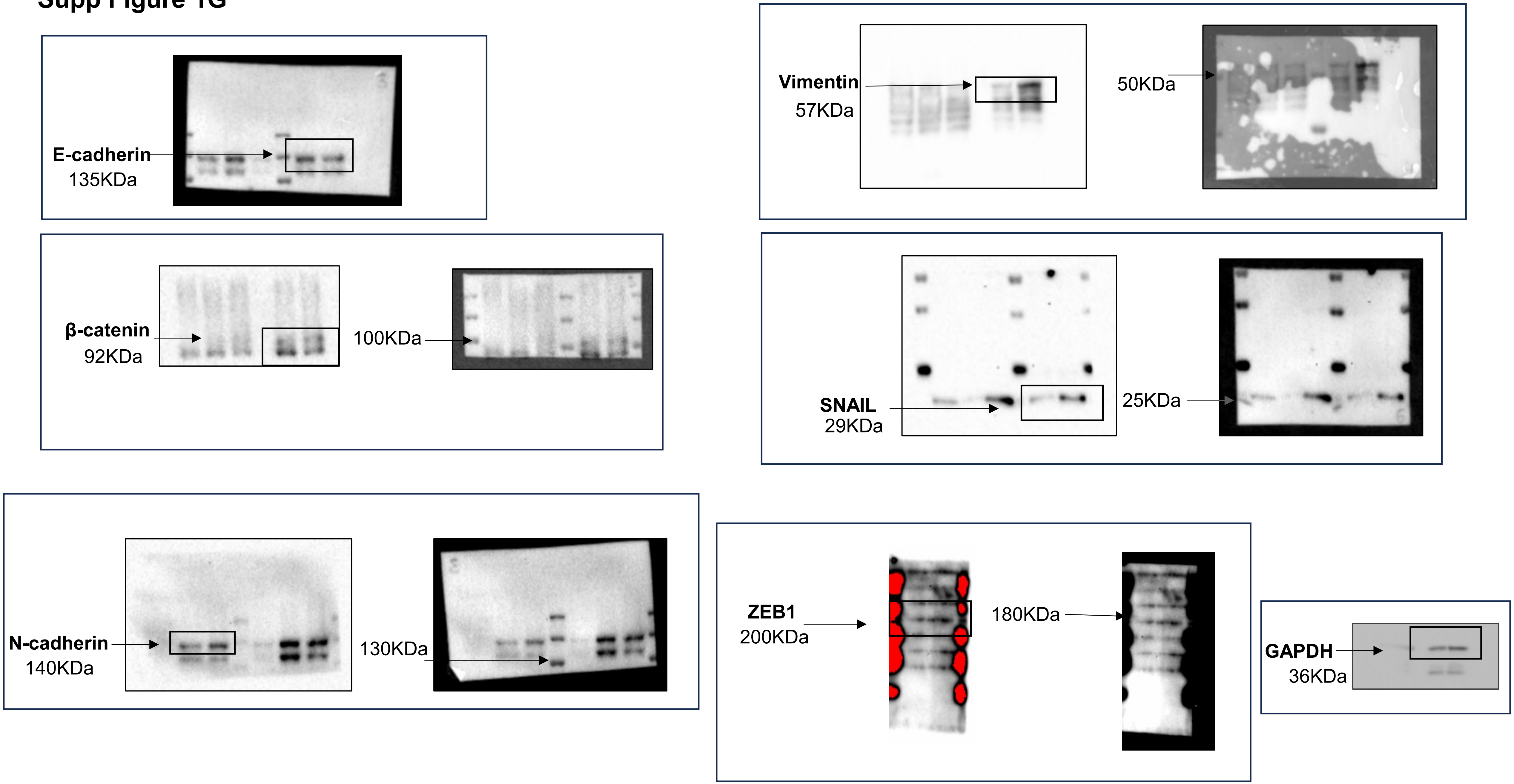

Supp Figure 2D

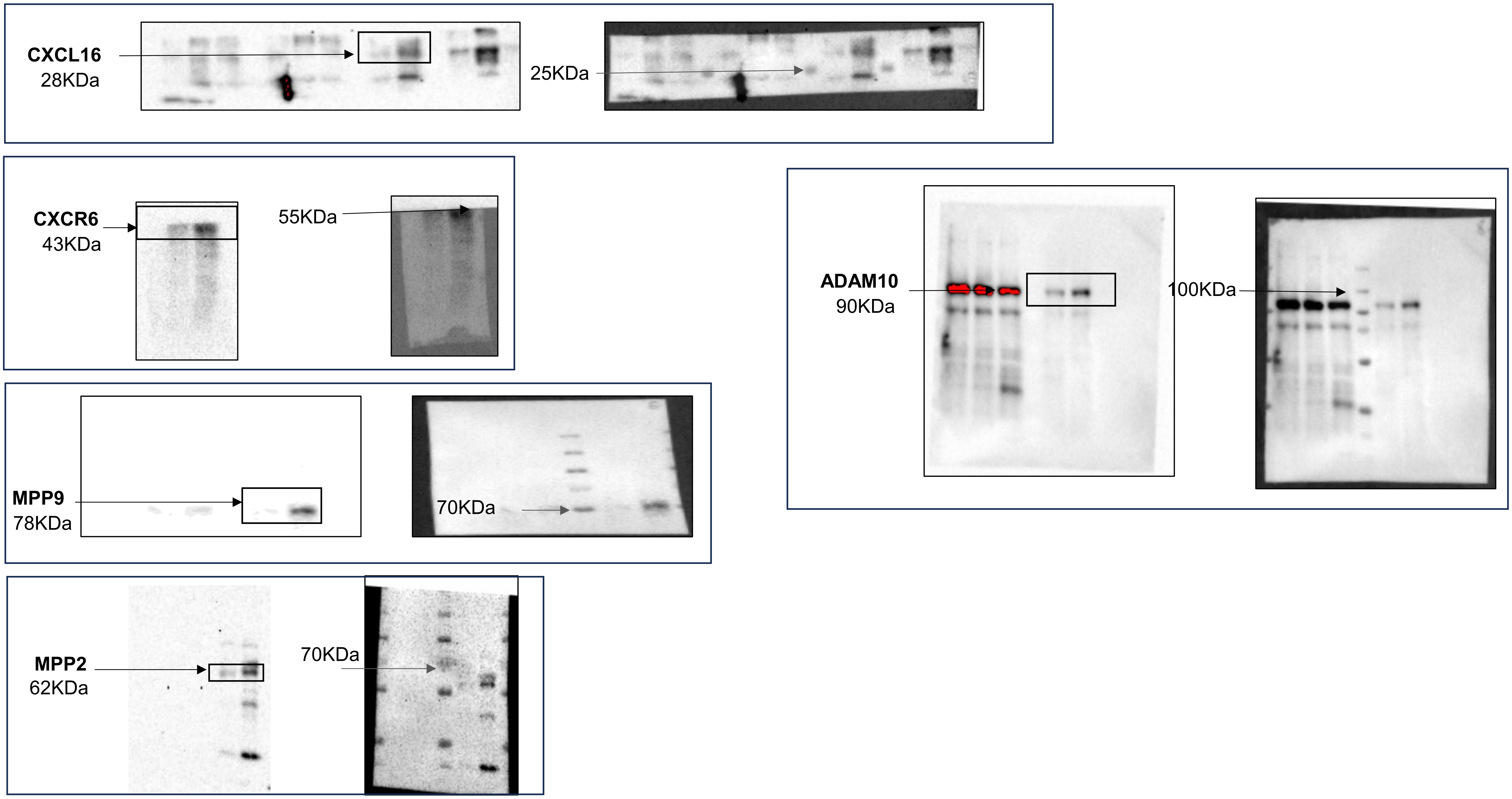

Supp Figure 2D

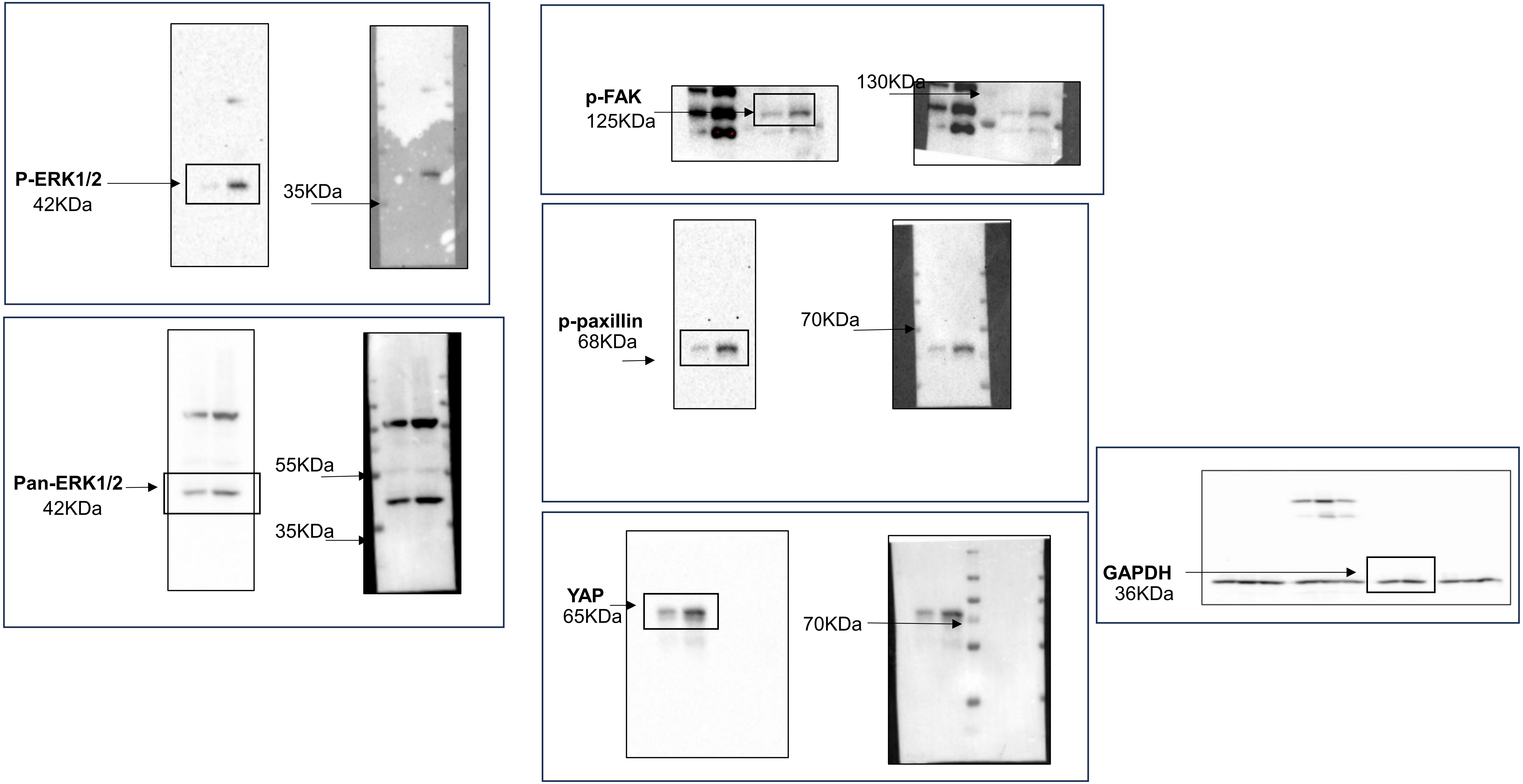

Supp Figure 3E

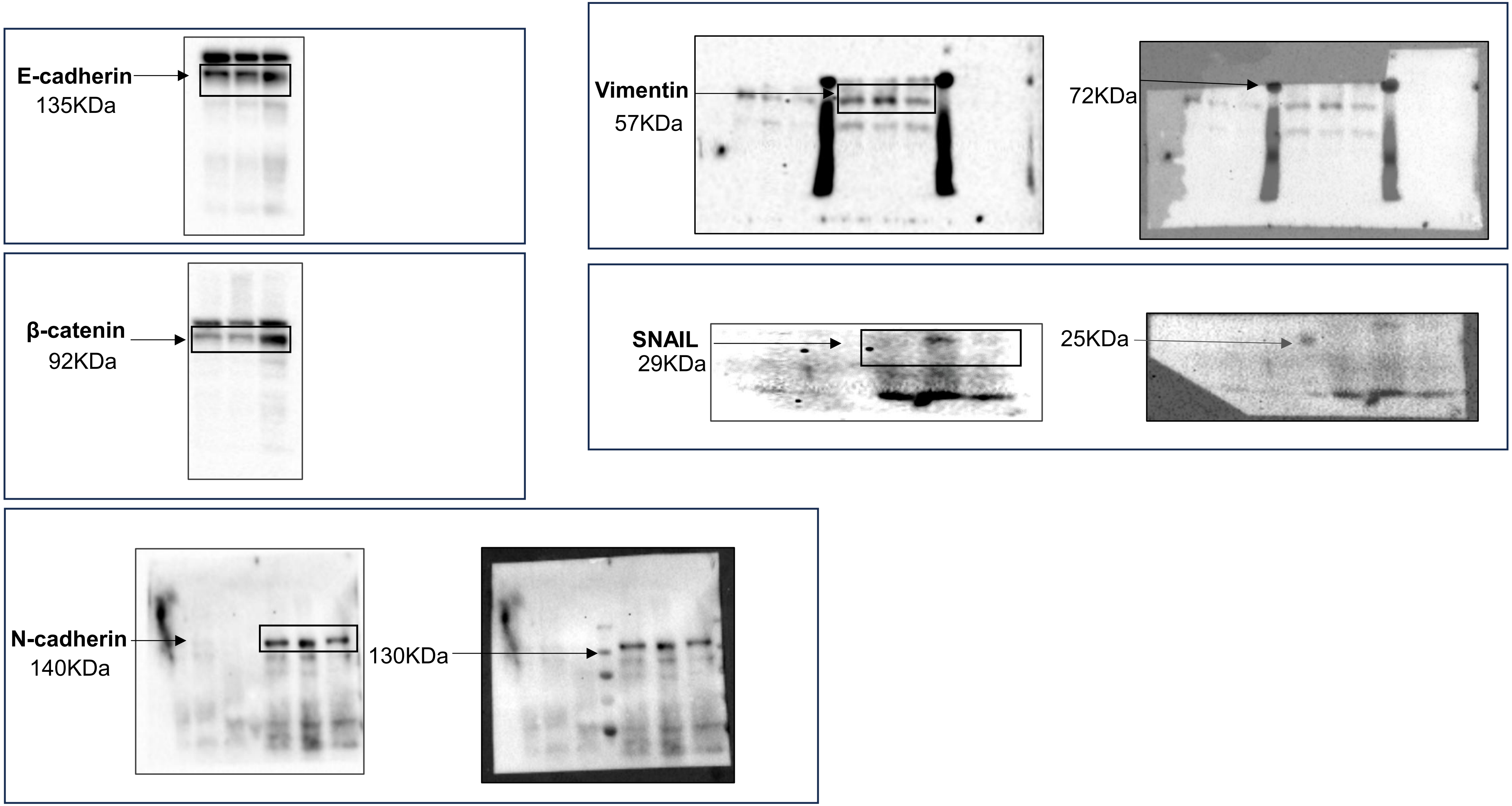

Supp Figure 3E

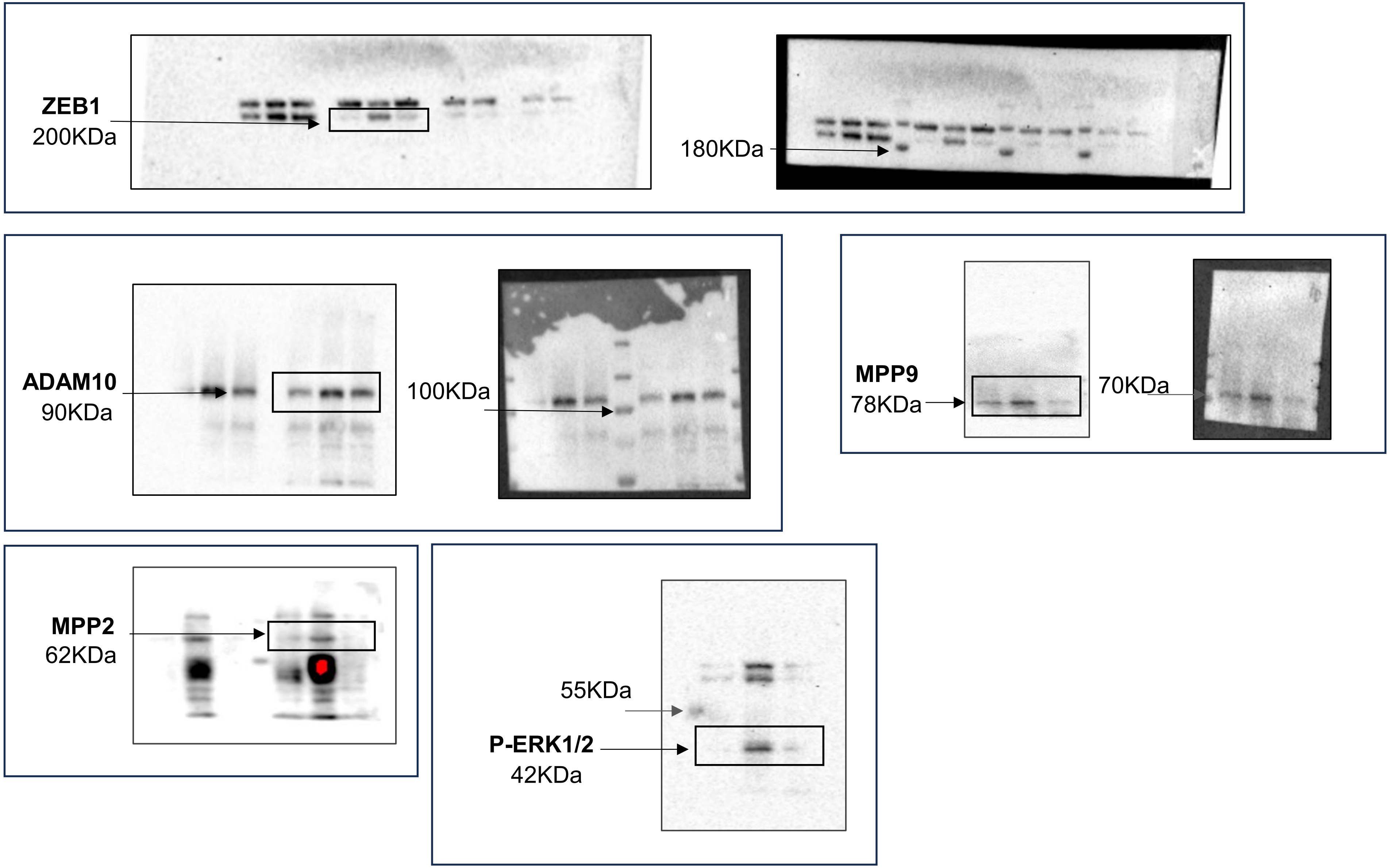

Supp Figure 3E

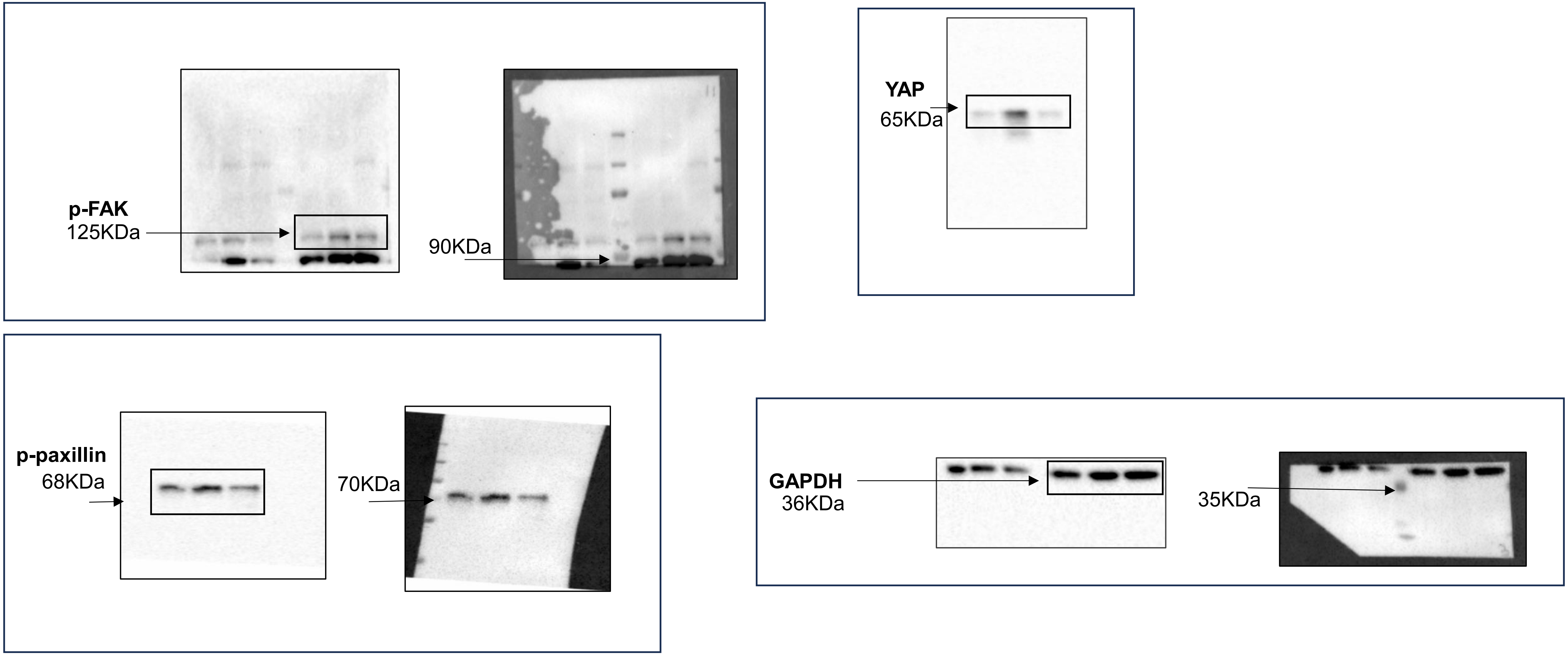

Supp Figure 4A

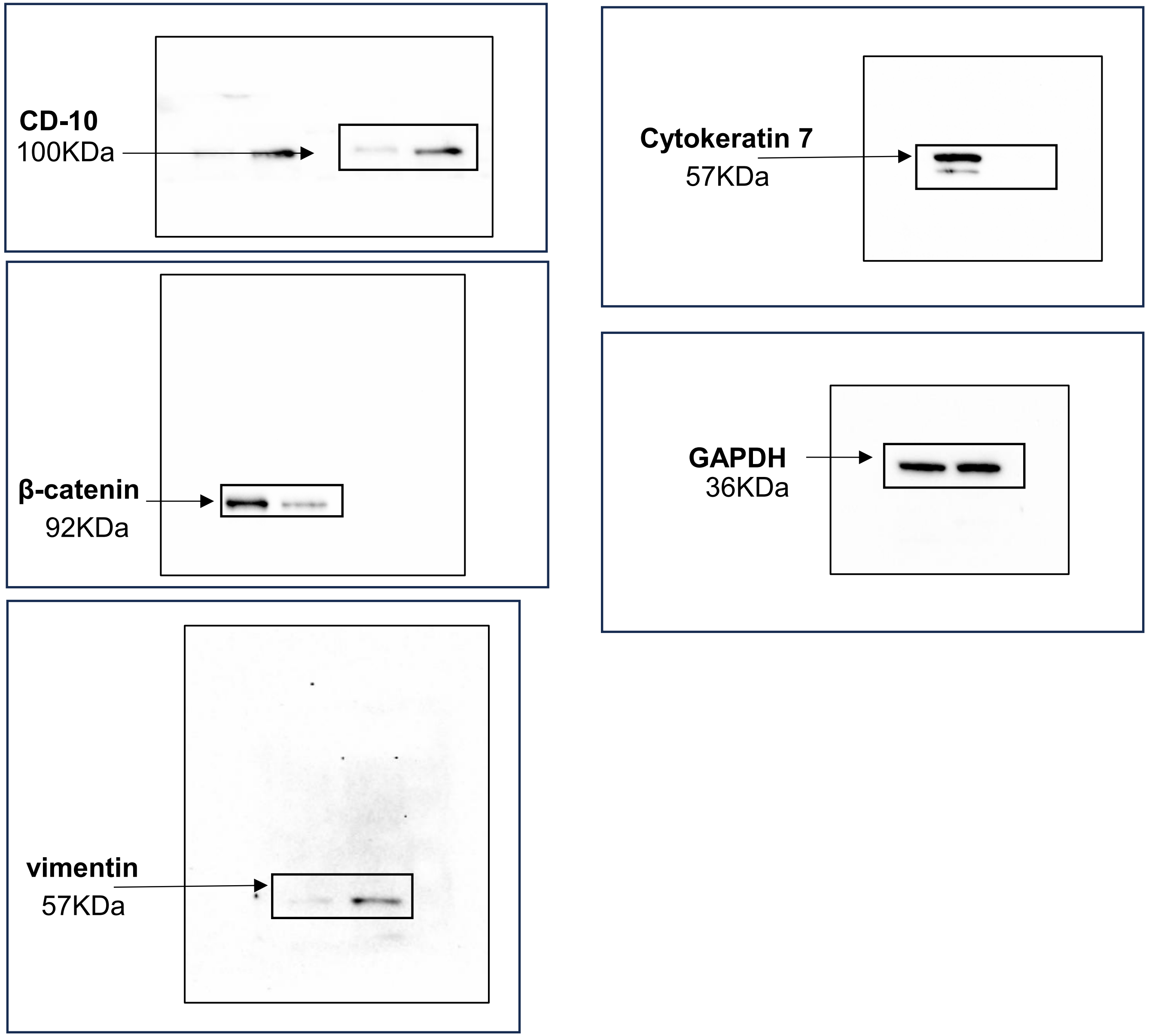

Supp Figure 5C

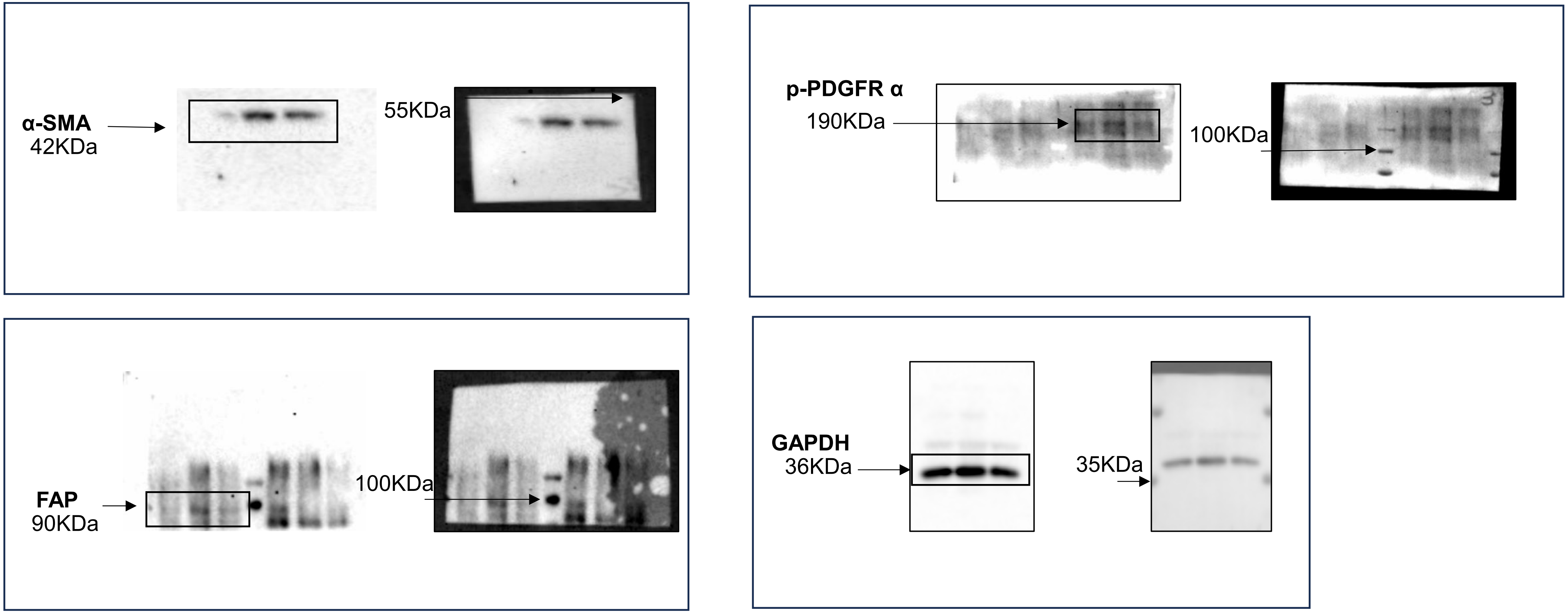

Supp Figure 5E

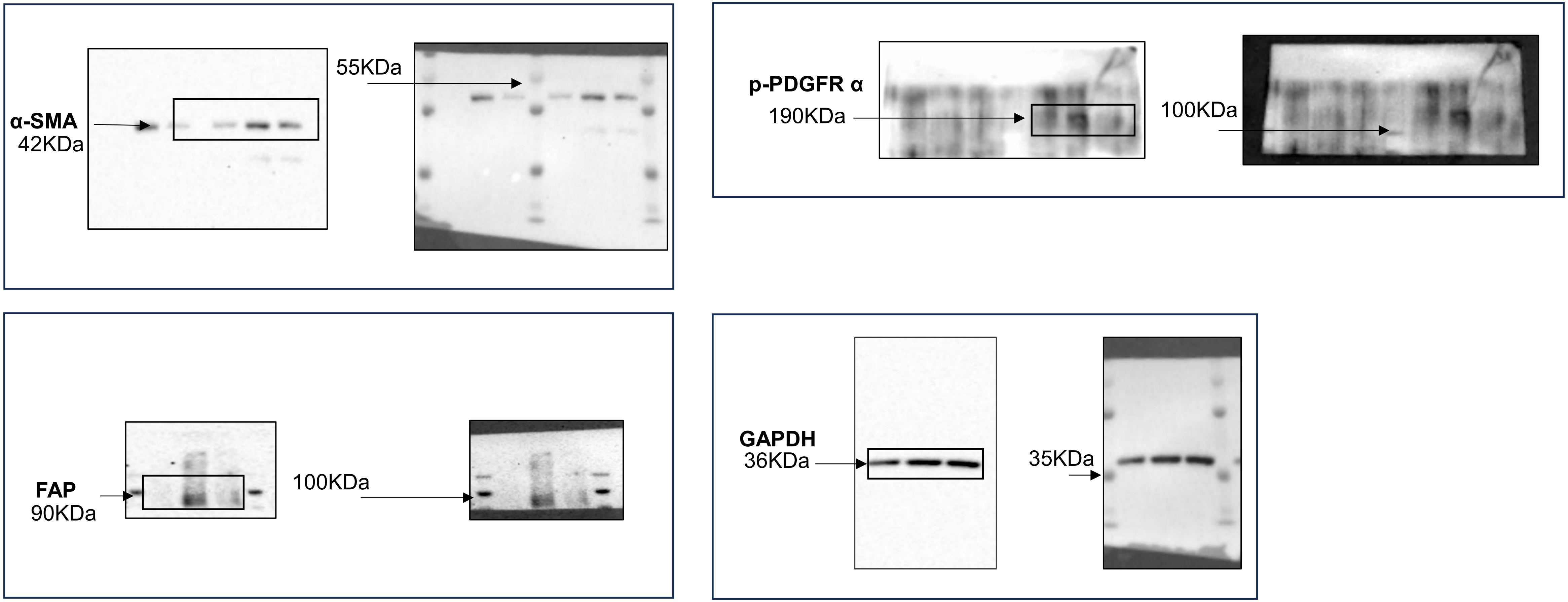

Supplement: Supplementary file 2 — Raw Data Western Blot [file 41419_2026_8723_MOESM2_ESM.pdf]
